# Optical beam shaping induced reconfigurable magnetic domains

**DOI:** 10.1038/s41467-026-77572-3

**Published:** 2026-09-15

**Authors:** Muhammad Waleed Khalid, Sheena K. K. Patel, Koffi-Emanuel Sadzi, Mohammed Salah El Hadri, Sergio A. Montoya, Stéphane Mangin, Eric E. Fullerton, Abdoulaye Ndao

**Affiliations:** 1 Department of Electrical and Computer Engineering, University of California—San Diego, La Jolla, CA 92093 USA; 2 Center for Memory and Recording Research, University of California—San Diego, La Jolla, CA 92093 USA; 3 Western Digital Corporation, 5601 Great Oaks Parkway, San Jose, CA 95119 USA; 4 IJL, CNRS, Université de Lorraine, F-54000 Nancy, France

**Keywords:** Optical data storage, Information storage

## Abstract

Ultrafast all-optical switching (AOS) in ferromagnets has emerged as a promising route toward dense and energy-efficient magnetic memory. However, its implementation has typically been limited by material-specific polarization requirements or by the need for auxiliary exchange coupling and compositional tuning. Here, we demonstrate that deterministic control of AOS can instead be achieved purely through optical engineering. By tailoring the polarization distribution and focusing of the excitation light, we shift the selectivity from intrinsic material properties to external optical beam shaping. In stand-alone [Pt/Co]_*N*_ multilayers, this enables direct control over local heating and optical torques, allowing the system to be driven either into helicity-dependent, multishot domain-wall propagation or into helicity-independent magnetization reversal, without material modification or external bias fields. Our results establish optical beam design as a universal control knob for magnetization dynamics in ferromagnetic systems, defining a scalable pathway for integrating ultrafast spintronic functionality with on-chip photonics.

## Abstract (web summary)

All-optical switching of the magnetic state offers the potential for ultrafast writing of information in the form of magnetic bits. While it has been implemented in simple and technologically relevant magnetic stacks, these have hitherto required helicity control. Here, Khalid and coauthors demonstrate how optical tailoring can overcome helicity constraints in /Pt/Co stacks.

## Introduction

Harnessing femtosecond light pulses to engineer and control magnetic order has recently emerged as a paradigm-shifting approach in spintronics, promising ultrafast information writing on sub-picosecond timescales while simultaneously enabling low-energy, nonvolatile data storage. The seminal discovery that femtosecond laser pulses can quench magnetization in ferromagnetic Ni^1^ prompted a shift from studying spin–lattice relaxation toward directly rewriting magnetic order with light. In ferrimagnetic alloys, intense optical excitation not only demagnetizes but can reverse magnetization polarity, either via all-optical helicity-dependent switching (AO-HDS) driven by circularly polarized light^2^ or helicity-independent “toggle” switching (AO-HIS) on picosecond timescales^3–7^. These processes arise from a complex interplay of ultrafast heating, angular momentum transfer, inverse Faraday effect (IFE), and magnetic circular dichroism (MCD)^8–11^.

Rare-earth ferrimagnets such as GdFeCo and TbFeCo have long served as model systems for studying ultrafast optical control of magnetization. However, their dependence on precise stoichiometry, operation near temperature-sensitive compensation points, and reliance on scarce elements limit both scalability and device stability^2–4,8,12^. Ferromagnetic [Pt/Co]_*N*_ multilayers provide a promising alternative, exhibiting robust perpendicular magnetic anisotropy (PMA) and tunable interfacial spin-orbit coupling at room temperature, which maintains high thermal stability even at nanometer-scale bit volumes^13,14^. In these stacks, AO-HDS proceeds via magnetic circular dichroism (MCD)-driven domain-wall motion over multiple pulses within a narrow optical fluence window. Initial pulses locally heat the material to the Curie temperature, nucleating reversed domains, while subsequent pulses exploit MCD-induced thermal gradients to expand domain walls until full reversal^15,16^.

Despite these advantages, [Pt/Co]_*N*_ systems face a key constraint: the intrinsic magnetic domain size often competes with the optical spot size. Increasing PMA and magnetic volume in high-*N* stacks reduces equilibrium domain dimensions, causing nucleated multidomain structures to freeze upon cooling and limiting deterministic switching. As a result, experiments have focused on low-*N* multilayers, where domain sizes exceed the illuminated area^17^. Early demonstrations of ferromagnetic AO-HIS relied on indirect mechanisms, including interlayer exchange coupling to a ferrimagnet and carefully tuned optical excitation matched to intrinsic domain structures^18–21^. While effective under specific conditions, these approaches offer limited control over the reversal pathway and often require complex multilayer engineering or precise alignment of the optical spot with magnetic microstructure.

One way to overcome the longstanding helicity constraints in technologically relevant ferromagnets requires shifting the control point from the material to the optics. High numerical aperture (*NA*) focusing and structured-light illumination compress the excitation volume and sculpt the spatial profile of energy absorption, redefining local temperature and torque landscapes on ultrafast timescales.

**Fig. 1:**
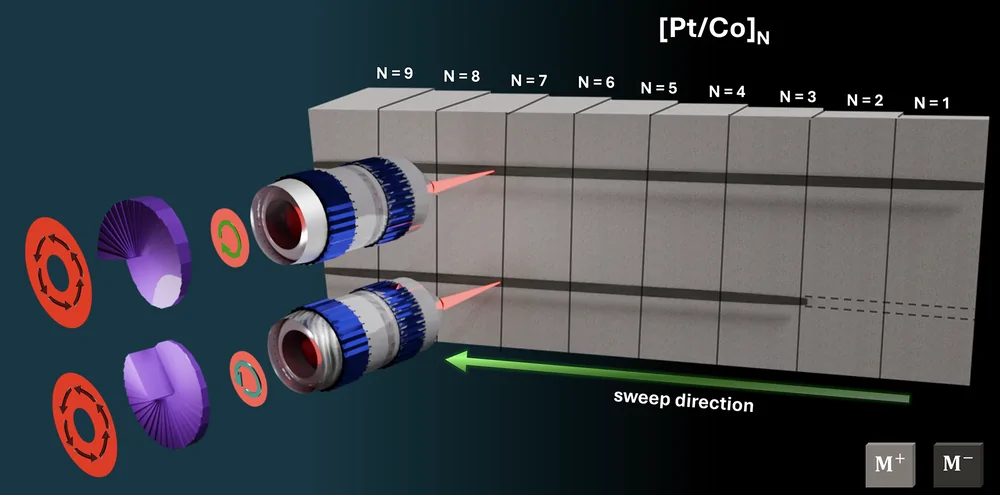
Optical beam shaping control of magnetization in [Pt/Co]_*N*_ multilayers. Conceptual illustration of circularly polarized optical beams interacting with [Pt/Co]_*N*_ stacks of different thicknesses. Varying optical focusing conditions and multilayer geometry allow transitions between helicity-dependent and helicity-independent switching. ${{{\bf{M}}}}_{{{\bf{z}}}}^{+}$ and ${{{\bf{M}}}}_{{{\bf{z}}}}^{-}$ denote the two possible magnetization states.

In this work, we will demonstrate that this subwavelength optical control tilts the balance between helicity-dependent (HD) and helicity-independent (HI) reversal pathways, enabling deterministic outcomes even in regimes previously dominated by helicity dependence. Using [Pt(0.7 nm)/Co(0.6 nm)]_*N*_ multilayers with *N* = 3–9 under high-*NA* focusing, we access a confined regime where ultrafast thermal gradients outweigh MCD bias, producing deterministic AO-HIS in stacks previously restricted to HD reversal. By directly identifying the transition at *N* = 3 and mapping its evolution across *N* = 1–9 at fixed *NA*, we uncover a universal optical-geometry threshold governing the HD-to-HI transition (see Fig. 1). All helicity-independent switching reported in this work occurs in a multi-pulse, thermally driven regime, which is fundamentally different from single-pulse toggle switching mechanisms, reported previously. This strategy achieves robust, field-free, and polarization-independent magnetization reversal in metallic ferromagnets, without altering material composition, applying external fields, or modifying pulse duration, while providing a general framework for understanding and controlling AOS in these systems^16,22^.

## Results

To control magnetization at subwavelength scales, we use circularly polarized vector beams (CPVBs)^23–27^. A CPVB is generated by preparing an azimuthally polarized field and then imprinting a vortex phase plate (VPP) of charge *ℓ* with transmittance *T*(*ϕ*) = *e*^±*iℓϕ*^, followed by tight focusing (see Materials and Methods). In the circular polarization basis $\{{\hat{{{\bf{e}}}}}_{R},{\hat{{{\bf{e}}}}}_{L}\}$, the azimuthal unit vector can be written as $\hat{{{\boldsymbol{\phi }}}}=\frac{i}{\sqrt{2}}\,\left({e}^{-i\phi }\,{\hat{{{\bf{e}}}}}_{L}-{e}^{+i\phi }\,{\hat{{{\bf{e}}}}}_{R}\right)$ so an azimuthally polarized input with radial envelope *P*(*r*) is

1 $${{{\bf{E}}}}_{\phi }(r,\phi )=P(r)\,\hat{{{\boldsymbol{\phi }}}}=P(r)\,\frac{i}{\sqrt{2}}\,\left({e}^{-i\phi }\,{\hat{{{\bf{e}}}}}_{L}-{e}^{+i\phi }\,{\hat{{{\bf{e}}}}}_{R}\right).$$

After a VPP of charge *ℓ*, one circular component loses its phase singularity, and its on-axis intensity null, yielding

2 $${{{\bf{E}}}}_{{{\rm{VB}}}}(r,\phi )=\frac{i\,P(r)}{\sqrt{2}}\left[{e}^{i(\ell -1)\phi }\,{\hat{{{\bf{e}}}}}_{L}-{e}^{i(\ell+1)\phi }\,{\hat{{{\bf{e}}}}}_{R}\right].$$

The focal field can be expressed in a simplified form as

3 $$\begin{array}{l}E({r}_{2},{\phi }_{2},z)=-\frac{iC}{\lambda }\int_{0}^{{\theta }_{{{\rm{max}}}}}\int_{0}^{2\pi }\sin \theta \,\sqrt{\cos \theta }\,M\,{E}_{t}\,\\ \times \exp \left[ik\left(z\cos \theta+{r}_{2}\sin \theta \cos (\phi -{\phi }_{2})\right)\right]\,d\phi \,d\theta,\end{array}$$

where (*r*_2_, *ϕ*_2_, *z*) denote the cylindrical coordinates in the focal region, C is a scaling constant. *θ*_max_ is set by the (*NA*), transfer matrix *M* accounts for the vectorial polarization response of the system, and *E*_*t*_ is the transmitted electric field amplitude. This expression describes the electric field distribution near the focus of a high-*NA* objective, incorporating both scalar diffraction terms and the vector nature of light. A detailed derivation of Eq. (3), together with the explicit form of *M* and the input field distribution *E*_*t*_, is provided in Supplementary Sections S2 and S3.

Two features make CPVBs advantageous for our high-*NA* opto-magnetic experiments. First, they preserve transverse polarization at focus while suppressing the longitudinal (*E*_*z*_) component, unlike tightly focused circularly polarized Gaussian beams that suffer from polarization distortion and non-negligible *E*_*z*_^28^. Second, CPVBs enable stronger spatial confinement: for the same focusing *NA*, the focal spot (intensity width) is reduced by up to ~15% compared with a Gaussian beam (Supplementary 4). A smaller spot size concentrates the light into a tighter surface area. This makes the light interact more strongly with the material and increases the energy per unit area delivered to the switched region. In our [Pt/Co]_*N*_ films, CPVBs reach the AOS threshold without inducing non-deterministic thermal demagnetization that occurs with Gaussian beams under similar conditions.

**Fig. 2:**
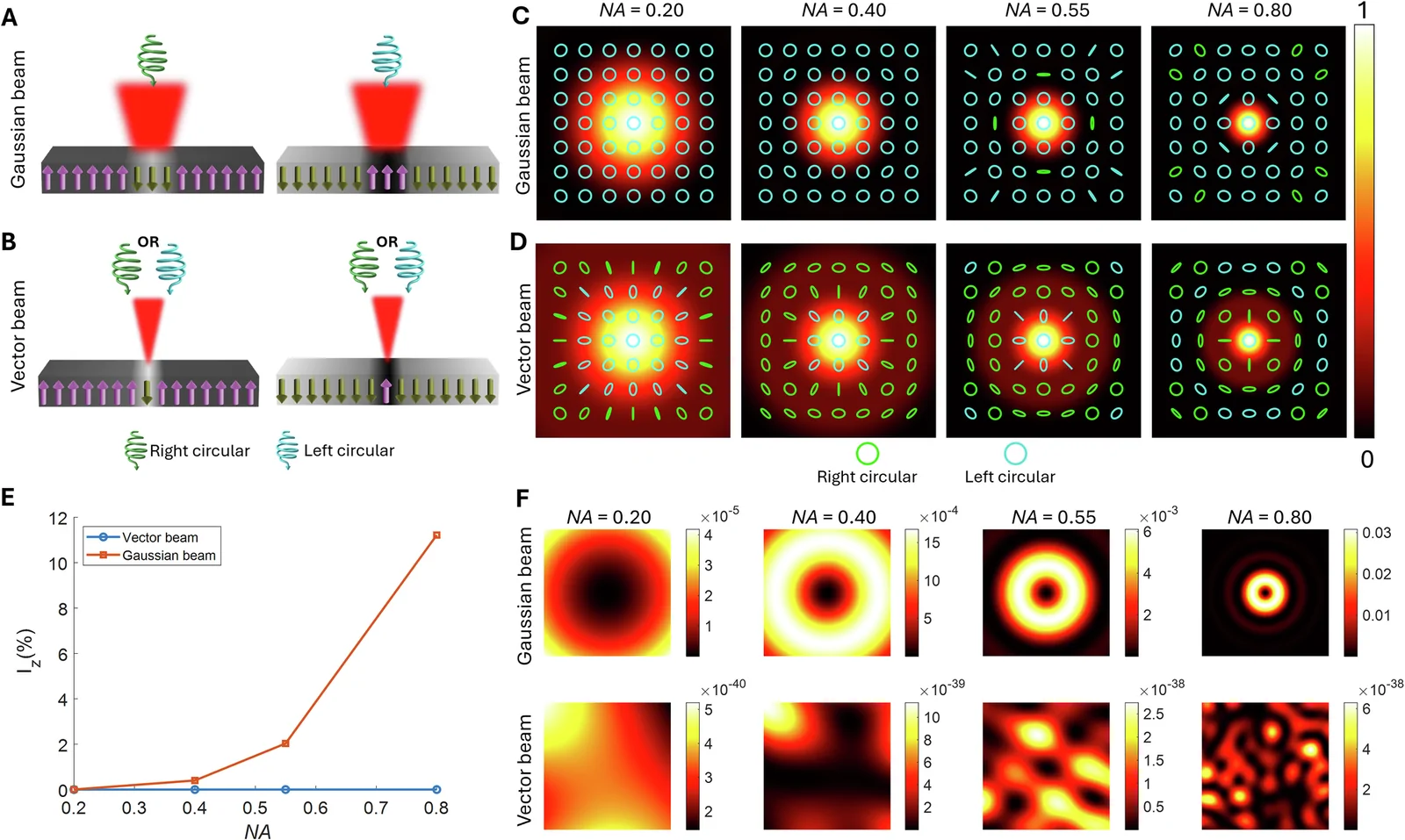
Optical control of helicity-dependent and helicity-independent switching in [Pt/Co]_*N*_ multilayers. **A**, **B** Schematic of magnetic dipole configurations in extended ferromagnetic films. Magnetic states pointing upward and downward are shown by purple and yellow arrows, respectively. The black/white contrast denotes opposite magnetic domains. Green (left-circular) and blue (right-circular) arrows denote the helicity of light at the focal plane. **A** AO-HDS under large-spot Gaussian illumination. Purple (upward) and yellow (downward) arrows indicate the orientation of magnetic dipoles. **B** AO-HIS achieved with a reduced spot size using a CPVB. In this regime, dipolar fields stabilize and hold the reversed domains. Orange looping arrows trace dipolar closures within the sample. Black/white contrast denotes opposite magnetic domains. **C**, **D** Focal-plane intensity and polarization distributions at *NA* = 0.20, 0.40, 0.55, and 0.80. Polarization ellipses are superimposed on the intensity profiles, with green (left-handed) and blue (right-handed) circles indicating the sense of polarization rotation corresponding to the light helicity. **E** Comparison of the longitudinal electric-field content at focus for a circularly polarized Gaussian beam (orange) and vector beam (blue). **F** Focal-plane intensity maps of the longitudinal field component, ∣*E*_*z*_∣^2^, for (top row) a Gaussian beam and (bottom row) vector beam, shown at *NA* = 0.20, 0.40, 0.55, and 0.80.

Figure 2 compares AO-HDS and AO-HIS under two distinct illumination schemes. In Fig. 2**A**, a circularly polarized Gaussian beam with a focal spot larger than the equilibrium domain size produces conventional helicity-dependent switching. In Fig. 2B, a circularly polarized vector beam (CPVB) reduces the excitation volume and instead enables helicity-independent reversal. The corresponding focal-plane polarization and intensity distributions are shown in Fig. 2C, D as a function of (*NA* = 0.20, 0.40, 0.55, 0.80), matching the conditions used in our experiments. The CPVB maintains a circularly polarized high-intensity core at focus while acquiring mixed polarizations away from the focal plane, whereas the Gaussian beam preserves a predominantly *z*-invariant polarization in the transverse plane (see Supplementary Figs. S3 and S4). Fig. 2E, F quantify how optical beam topology governs the longitudinal field generated by high-NA focusing. Under vectorial focusing analysis, a circularly polarized Gaussian beam acquires a nonzero *E*_*z*_ because the electric field is forced to converge along the propagation direction; the longitudinal contribution therefore grows with NA. CPVBs have an azimuthally symmetric polarization around the optical axis, so symmetry requires *E*_*z*_(0) = 0. As a result, their longitudinal content remains negligible even at large *NA*. We compute the longitudinal fraction *I*_*z*_ by integrating ∣*E*_*z*_∣^2^ over the focal plane and normalizing by the total power at focus, assuming equal incident power and a standard aplanatic pupil.

4 $${I}_{z}=\frac{\int | {E}_{z}(x,y){| }^{2}\,{{\rm{d}}}A}{\int \left(| {E}_{x}{| }^{2}+| {E}_{y}{| }^{2}+| {E}_{z}{| }^{2}\right)\,{{\rm{d}}}A},$$

While deterministic switching in the Pt/Co multilayers is governed by lateral energy confinement and thermally driven processes, the use of vector beams suppresses longitudinal field components at high NA, ensuring desired polarization at the focal plane and enabling a clearer separation of thermal and polarization-related effects.These polarization observations establish the optical-geometry conditions underlying the transition from HD to HI switching in [Pt/Co]_*N*_ multilayers *N* = 3.

**Fig. 3:**
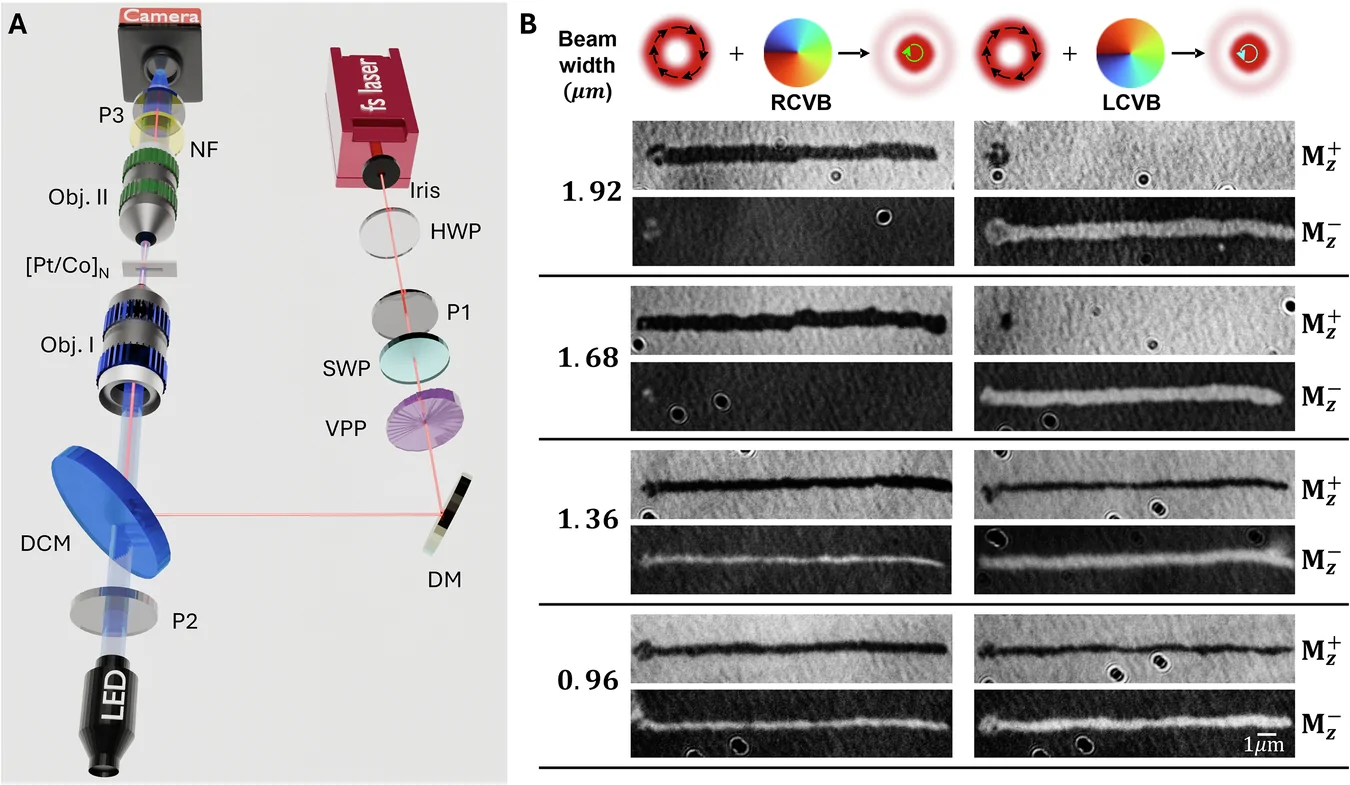
Transitioning from helicity-dependent to helicity-independent switching through optical beam shaping. **A** Optical setup used in the experiments. A linearly polarized fs laser is trimmed in size using the *iris*. HWP (half-wave plate) and P1 (polarizer) are used to control power and set the reference polarization. CPVBs are generated by SWP (S-waveplate) followed by VPP (vortex phase plate). CPVB pulses are reflected using a DM (dielectric mirror) and combined with LED illumination at a DCM (dichroic mirror). LED and fs pulses are focused onto the sample by Obj I (focusing objective) and the image is relayed to the camera by Obj II (imaging objective) through an NF (800 nm notch filter) to block fs pulses. A Faraday microscope is formed by P2 (polarizer) and P3 (analyzer), providing magnetization contrast. See Materials and Methods for details. **B** Left column: right-circular vector beam (RCVB, *ℓ* = + 1) generated using a VPP with topological charge *ℓ* = + 1; right column: left-circular vector beam (LCVB) produced by a VPP with charge *ℓ* = − 1. ${{{\bf{M}}}}_{{{\bf{z}}}}^{+}$ and ${{{\bf{M}}}}_{{{\bf{z}}}}^{-}$ denote the two stable magnetization states. Magnetization switching results are shown for decreasing effective spot sizes, from 1.92 to 0.96 μm, where the laser is scanned from left to right at a sweep speed of 1 μm/s. For larger spots, the reversal is helicity dependent, with final states determined by the optical beam handedness. As the spot size decreases, the helicity contrast diminishes, and below ~1.36 μm the reversal becomes helicity independent.

To directly probe the transition from helicity-dependent to helicity-independent switching, we examined [Pt/Co]_*N*_ multilayers with *N* = 3 under illumination by circularly polarized vector beams (CPVBs) generated using vortex phase plates (VPPs) (see Fig. 3). The right-circular (RCVB, *ℓ* = + 1) and left-circular (LCVB, *ℓ* = − 1) beams were scanned across the sample, from left to right fro all samples, while the focal spot size was systematically reduced from 1.92 to 0.96 μm at a sweep speed of 1 μm/s. For large spot sizes, the final magnetic state was helicity dependent, consistent with conventional AO–HDS behavior in which magnetic circular dichroism biases domain wall motion. However, as the effective spot size was reduced, the helicity contrast progressively weakened. Once the spot size reached ~1.36 μm and below, the reversal became HI, with both RCVB and LCVB beams producing identical magnetization outcomes. The sweep speed was chosen to ensure that the material experienced sufficient pulse overlap for the domains to converge into a deterministic state, as shown in Supplementary Section 6. Control experiments at reduced pulse counts demonstrate that insufficient number of pulses can mimic helicity independence, highlighting the need for careful evaluation of sweep conditions. Together, these observations establish spot-size confinement as a robust route to induce HI reversal in ferromagnetic multilayers.

**Fig. 4:**
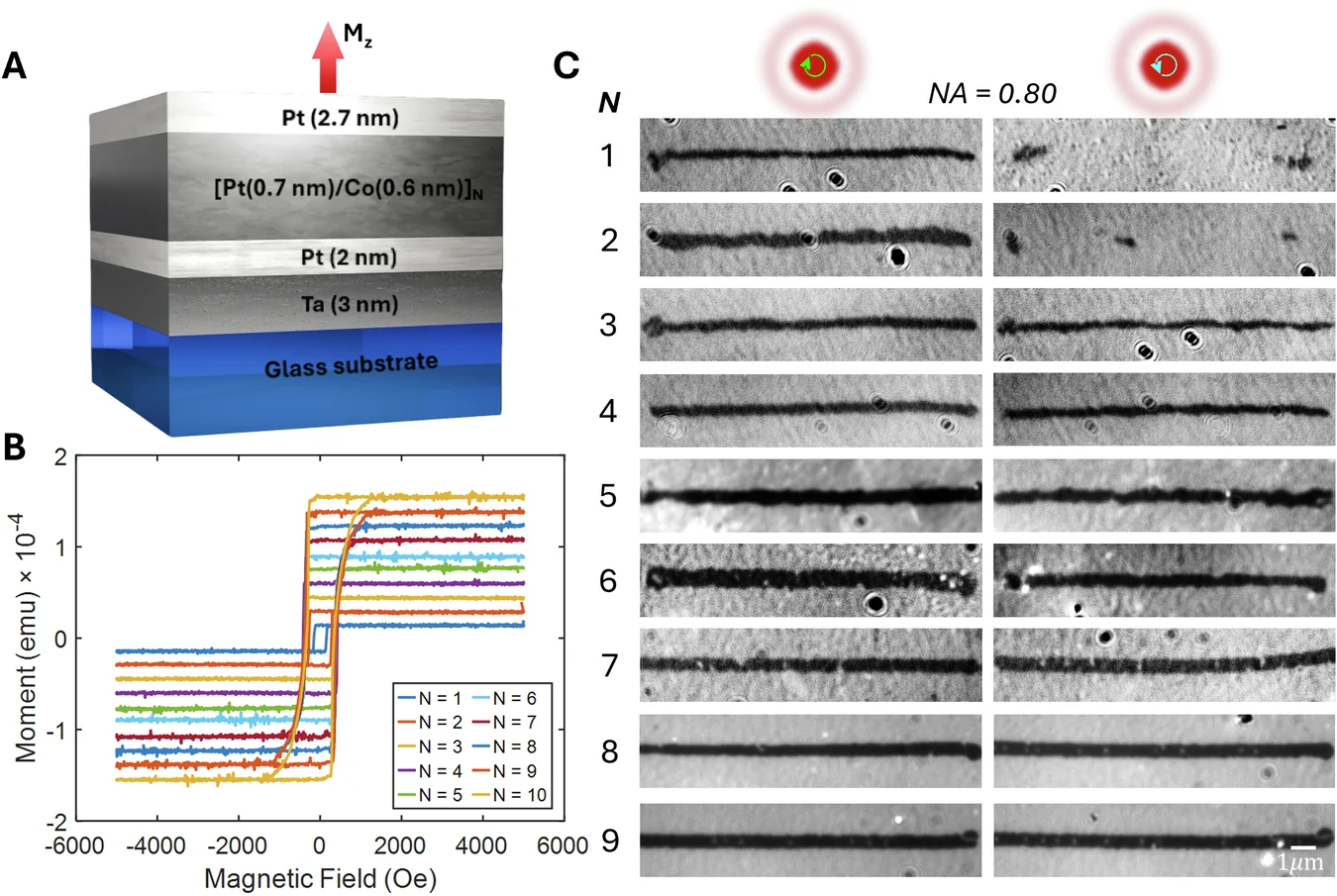
Material dynamics and magnetization control for *N*-repetition multilayers. **A** Schematic of the thin-film stack with individual layer thicknesses. Red arrow indicates the out-of-plane magnetization of the [Pt/Co]_*N*_ multilayer. **B** Out-of-plane hysteresis loops for representative *N*, showing that increasing the number of bilayer repeats leads to higher coercivity and larger saturation magnetization. **C** shows the magnetic reversal results for *N* = 1–9. All the sweeps here were performed using a lens of *NA* = 0.8 and initial magnetization state is ${{{\bf{M}}}}_{{{\bf{z}}}}^{+}$ for all the sweeps, where the laser was scanned from left to right. Left and right columns show the results for incident RCVB and LCVB, respectively. For *N* = 1, 2, RCVB and LCVB produce different final magnetization state depending on the helicity of the incident light. At *N* = 3, the two helicities yield largely similar final magnetic states with isolated differences. From *N *≥ 4, the RCVB and LCVB outcomes are indistinguishable within experimental contrast as there is no observable helicity dependence. For *N* = 5–9, the final states are predominantly identical for both helicities.

Figure 4 summarizes the switching behavior across [Pt/Co]_*N*_ multilayers (*N* = 1–9) at fixed *NA* = 0.80. For the thinnest stacks (*N* = 1, 2), reversal is strongly helicity dependent. Starting at *N* = 3, dipolar fields from the surrounding unexcited film increasingly bias the relaxation of the laser-affected region, competing with magnetic circular dichroism (MCD)-driven domain-wall motion. As a result, both HD and HI outcomes can be accessed depending on spot size (see Fig. 3). From *N* = 4 onward, AO-HIS dominates, with helicity contrast disappearing across all focusing conditions (see Fig. S9). In thicker films (*N* = 5–9), deterministic AO-HIS persists only under wavelength-scale focusing, while larger spots relax into multi-domain states. Hence, we only perform experiments for these (*N* = 5–9) multilayer using a lens with *NA* = 0.8. Beyond *N* = 9, stripe-domain textures arising from the competition between perpendicular magnetic anisotropy and dipolar interactions prevent uniform reversal, resulting in partial switching (see Fig. S10). Collectively, these results identify a criterion set by optical beam spot size and dipolar stabilization which govern the crossover from helicity-dependent to helicity-independent reversal in ferromagnetic multilayers.

## Discussion

AOS in Pt/Co ferromagnets reflects a balance between a helicity independent photo-thermal effects, where ultrafast heating drives the system into a low-coercivity state near *T*_C_ and helicity-dependent mechanisms, notably MCD, that can bias remagnetization process via domain-wall motion under repeated circularly polarized laser pulses. In thin stacks (*N* = 1–2), the demagnetizing (dipolar) fields surrounding the laser-heated region are comparatively weak, so helicity-dependent absorption gradients more readily steer domain growth, yielding the well-known AO-HDS at practical spot sizes^15^. As the number of bilayers *N* increases, the total magnetic thickness and magnetostatic energy scale up, strengthening the dipolar fields that act on the laser-affected zone and enabling an AO-HIS outcome when those fields can stabilize a single reversed circular-shaped magentic region during the quench. Optical beam-size control selects which mechanism dominates. Reducing the effective optical beam spot, using a high *NA* lens, increases the local energy density and accelerates thermal cycling while simultaneously shrinking the volume that must be stabilized on cooling. When the spot approaches the wavelength scale, the photo-thermal route dominates and the surrounding film’s dipolar field becomes the decisive bias, overwhelming helicity-selective MCD contributions and producing AO-HIS. This picture is consistent with vectorial focusing: wavelength-scale illumination minimizes longitudinal field components and concentrates energy within a compact lateral area, conditions that favor the nucleation of circular-shaped magentic region, which is followed by dipolar stabilization.

The measured trends follow directly from this framework. For *N* = 3 we observe a clear transition: at larger spots the final state tracks the light helicity, whereas in the wavelength-scale regime the helicity contrast collapses and the switching becomes HI, (see Fig. 3 and Fig. S8). For *N* = 4, AO-HIS is obtained across all tested optical beam spot sizes, indicating that dipolar stabilization is strong enough to impose a unique final state even without wavelength-scale confinement (Supplementary Section 6). For *N *≥ 5, AO-HIS appears only when the spot is on the order of *λ*; at larger spots the exposure relaxes into random multidomain patterns because dipolar stabilization is insufficient to form an isolated magnetic switched region. For *N *≥ 10, the emergence of stripe domains, which is set by the competition between perpendicular anisotropy and magnetostatics, precludes uniform reversal and only partial switching is obtained (Fig. S10). The ability to independently tailor optical beam shape and polarization thus opens a regime of field-driven dynamics previously inaccessible under standard Gaussian beam focusing.

Single-shot toggle-type AO-HIS is not expected in the present [Pt/Co]_*N*_ multilayers, which are purely ferromagnetic and lack the ferrimagnetic sublattice dynamics responsible for deterministic single-pulse switching observed in ferrimagnets, such as GdFeCo^4,8,29^. In standalone [Pt/Co]_*N*_ multilayers, single-pulse excitation leads to demagnetization and domain nucleation, with deterministic reversal emerging only through multi-pulse thermal accumulation or exchange coupling to a ferrimagnet, while lateral stabilization by surrounding domains further suppresses toggle-type reversal^16,30–32^. In our experiments, multi-pulse measurements show no deterministic single pulse reversal, but instead, magnetic switching proceeds cumulatively via nucleation and growth under repeated pulses. The dipolar field dominates the switching criterion when the wavelength-scale focusing condition is met.

In summary, we demonstrate a versatile optical beam-engineering approach that enables deterministic control of all-optical magnetization switching in technologically relevant ferromagnetic multilayer. By co-optimizing the multilayer geometry and optical excitation conditions, robust polarization-independent switching is achieved at sub-wavelength length scales, overcoming the helicity constraints, which can limit the device integration. Future studies investigating material-dependent switching thresholds and the dynamic evolution of magnetic domains will provide further insight into the underlying mechanisms and guide the rational design of next-generation opto-magnetic platforms capable of ultrafast, all optical data storage.

## Methods

### Deposition of Pt/Co thin films

Pt/Co multilayer thin films consists of Ta(3 nm)/Pt(2 nm)/[Pt(0.7 nm/Co(0.6 nm)]_*N*_/Pt(2.7 nm) where *N* = 1, 2, …, 10, were deposited at room temperature by DC magnetron sputtering. Films for optical measurements were grown on polished glass substrates (~3.9 × 4.1 mm^2^), while films for magnetic characterization were deposited on Si substrates with a 300 nm thermally grown SiO_2_ layer. The base pressure in the sputtering chamber was maintained at 5 × 10^−8^ Torr, and the Ar process pressure was held at 2.5 mTorr with a flow rate of 50 sccm. Magnetometry measurements were performed using vibrating sample magnetometry (VSM) in out-of-plane geometries to obtain hysteresis loops and evaluate magnetic anisotropy.

### Experimental generation of CPVB

To generate azimuthally polarized light, we started with a linearly polarized Gaussian beam, which was directed through an S-waveplate (LaserAND, Inc), which is a spatially variant half-wave plate with a local fast-axis orientation *θ*(*ϕ*) = *mϕ* + *δ*, where *m* = 1 for our case, *δ* = 0, and *ϕ* is the azimuthal coordinate. The S-waveplate rotates the local polarization by 2*θ*(*ϕ*), producing a spatially varying linear polarization vector $(-\sin \phi,\,\cos \phi )$ corresponding to azimuthal polarization. A subsequent VPP (Holo/Or) acts as a polarization-selective filter, enabling helicity control through its topological charge *ℓ* = ± 1. This optical beam configuration concentrates circular polarization into the high-intensity central region, which is then focused using objective lens of varying *NA*. The optical beam width is defined as the intensity diameter at which the irradiance drops to 1/*e*^2^ of its peak value.

### Optical setup

The ferromagnetic sample was excited using a Spectra–Physics Solstice Ace Ti:sapphire regenerative amplifier delivering 120fs pulses at *λ* = 800nm and 1 kHz. The output optical beam (~10 mm diameter) was trimmed with an iris to 1 mm, a compromise that reduces diffraction while maintaining smaller size. A HWP followed by linear a polarizer is used power control since the laser is linearly polarized at the source. This polarizer, *P1*, also defines the reference polarization axis. This axis was aligned to the S-waveplate to generate a pure azimuthally polarized field, followed by a VPP. We use a dielectric mirror since it preserves the vectorial polarization distribution as it has near-equal reflectance for *x* and *y* polarizations.

We focus the vector beam with interchangeable microscope objectives of different *NA*, keeping all other conditions fixed. Femtosecond laser pulses and the LED illumination are focusing through the same focusing objective. The LED was intentionally defocused to avoid imaging the source onto the sample. Vector beam focal plane was verified by maximizing the camera intensity at the sample plane and also by centering the optical beam on the objective pupil to minimize polarization aberrations at focus. The initial magnetization state of each [Pt/Co]_*N*_ stack was prepared using a permanent magnet to saturate the film uniformly either (${{{\bf{M}}}}_{{{\bf{z}}}}^{+}$) or (${{{\bf{M}}}}_{{{\bf{z}}}}^{-}$). The sample was placed on a custom made holder which was held normal to the applied field to avoid introducing any in-plane component, ensuring a well-defined out-of-plane initial magnetization state prior. All the samples were consistently moved from left to right using a Thorlabs multi-axis motorized stage (PT3Z9).

Magnetic domain imaging was performed in a Faraday microscope configuration with the sample between crossed polarizers (extinction ratio 10^4^:1) to maximize contrast. Uniform back–illumination was provided by a Thorlabs M450D4 LED centered at *λ* = 450 nm to satisfy the Nyquist sampling criterion. A short–pass dichroic (cutoff 550 nm) reflected the 800 nm Ti:sapphire optical beam while transmitting the LED light, enabling real-time visualization of switching and rapid power optimization.

## Supplementary information

Supplementary Information

Peer Review File

## Data Availability

All data needed to evaluate the conclusions in this paper are available in the main text and/or the Supplementary Materials.
