# Supplementary material for: Optical beam shaping induced reconfigurable magnetic domains
Source: Nat Commun. 2026 Sep 15;17:10843. doi: 10.1038/s41467-026-77572-3 (PMC13672297; doi:10.1038/s41467-026-77572-3)
Supplement: Supplementary file 1 [file 41467_2026_77572_MOESM1_ESM.pdf]

Supplementary: Optical Beam shaping induced reconfigurable magnetic domains

Muhammad Waleed Khalid[1], Sheena K.K. Patel[2], Koffi-Emanuel Sadzi[1], Mohammed Salah El Hadri[3], Sergio A. Montoya[2], Stéphane Mangin[4], Eric E. Fullerton[2], and Abdoulaye Ndao[1, 2*]

[1*]Department of Electrical and Computer Engineering, University of California—San Diego, La Jolla, 92093, CA, USA.
[2]Center for Memory and Recording Research, University of California—San Diego, La Jolla, 92093, CA, USA.
[3]Western Digital Corporation, 5601 Great Oaks Parkway, San Jose, CA, 95119, USA.
[4]Université de Lorraine, CNRS, IJL, Université de Lorraine, Nancy, F-54000, France.

*Corresponding author(s). E-mail(s): a1ndao@ucsd.edu;

1 Electric Field Components and Polarization Analysis

Azimuthal Polarization (in Cartesian Basis)

The azimuthally polarized electric field can be expressed as:

$$\mathbf{E}_{\text{azi}}(r,\phi) = P(r)\,\hat{e}_\phi \tag{S1}$$

where

$$\hat{e}_\phi = -\sin\phi\,\hat{e}_x + \cos\phi\,\hat{e}_y \tag{S2}$$

and $P(r)$ is the radial amplitude envelope.

Rewrite in Circular Polarization Basis

The circular polarization basis vectors are defined as:

$$\hat{e}_R = \frac{1}{\sqrt{2}}(\hat{e}_x - i\hat{e}_y) \quad \text{(Right-hand circular)} \tag{S3}$$

$$\hat{e}_L = \frac{1}{\sqrt{2}}(\hat{e}_x + i\hat{e}_y) \quad \text{(Left-hand circular)} \tag{S4}$$

Inverting:

$$\hat{e}_x = \frac{1}{\sqrt{2}}(\hat{e}_R + \hat{e}_L), \quad \hat{e}_y = \frac{i}{\sqrt{2}}(\hat{e}_R - \hat{e}_L) \tag{S5}$$

Substituting into $\hat{e}_\phi$:

$$\hat{e}_\phi = \frac{1}{\sqrt{2}}\left(e^{i\phi}\hat{e}_R - e^{-i\phi}\hat{e}_L\right) \tag{S6}$$

Thus, the azimuthal polarization field becomes:

$$\mathbf{E}_{\text{azi}}(r,\phi) = P(r)\,\frac{1}{\sqrt{2}}\left(e^{i\phi}\hat{e}_R - e^{-i\phi}\hat{e}_L\right) \tag{S7}$$

Key point: Azimuthal polarization is already a coherent superposition of right- and left-circular polarized fields, each carrying opposite helical phases.

After Applying a Helical Phase Plate

Suppose we pass the beam through a helical phase plate introducing a phase factor $e^{im\phi}$.

The resulting field becomes:

$$\mathbf{E}_{\text{after phase plate}}(r,\phi) = P(r)\,\frac{1}{\sqrt{2}}\left(e^{i(m+1)\phi}\hat{e}_R - e^{i(m-1)\phi}\hat{e}_L\right) \tag{S8}$$

Effect:

- The RCP ($\hat{e}_R$) component now carries orbital angular momentum $m+1$.
- The LCP ($\hat{e}_L$) component carries orbital angular momentum $m-1$.

Focusing the Beam (High-NA Lens)

After tight focusing (modeled via Richards–Wolf integrals):

- Components with orbital charge $l = 0$ (zeroth-order Bessel functions) can produce nonzero intensity at the focus center ($r = 0$).
- Choosing m carefully allows one component (RCP or LCP) to dominate at the beam center.

Typical Focused Field Behavior:

- If $m = -1$, the RCP component dominates at the center.
- If $m = +1$, the LCP component dominates at the center.

Thus, by controlling the topological charge m, we can engineer the desired spin state at the beam center.

Amplitude Profile $P(r)$

For azimuthally polarized vortex beams (topological charge $l = \pm 1$), the radial profile is typically:

$$P(r) = E_0\left(\frac{r}{w_0}\right)^{|l|} e^{-r^2/w_0^2}$$

where:

- w_0 is the beam waist,
- E_0 is a normalization constant,
- $l = \pm 1$ for first-order azimuthal vortex beams.

Special case: For $l = 1$, the profile is:

$$P(r) = E_0 \frac{r}{w_0} e^{-r^2/w_0^2}$$

This ensures the field vanishes at the center $r = 0$, consistent with the presence of a phase singularity.

2 Vectorial Richards and Wolf diffraction integrals

These equations describe the electric field distribution in the focal region of a high numerical aperture lens (NA), considering vector diffraction effects. The following equations have been derived from Richards and Wolf (1959).

Equation 1: General Electric Field

The electric field at a point (x, y, z) is computed using the Debye diffraction integral:

$$E(x,y,z) = -\frac{iC}{\lambda} \int_0^{\theta_{\max}} \int_0^{2\pi} \sin\theta \, E_t \times \exp\left[ik(\cos\theta \, z - \sin\theta\cos\phi \, x - \sin\theta\sin\phi \, y)\right] d\phi \, d\theta \tag{S9}$$

Here:

- $\theta_{\max} = \arcsin(\text{NA})$, where NA is the numerical aperture of the optical system.
- λ is the wavelength of light.
- C is a scaling constant.
- E_t is the transmitted electric field amplitude.

Equation 2: Including the Matrix M

To account for the vectorial nature of light, we include the matrix M, which describes polarization transformations. The equation becomes:

$$E(x,y,z) = -\frac{iC}{\lambda} \int_0^{\theta_{\max}} \int_0^{2\pi} \sin\theta\sqrt{\cos\theta} \cdot M \cdot E_t \times \exp\left[ik(\cos\theta \, z - \sin\theta\cos\phi \, x - \sin\theta\sin\phi \, y)\right] d\phi \, d\theta \tag{S10}$$

Matrix M: Polarization Effects

The matrix M represents the transformation of electric field components during focusing:

$$\begin{bmatrix} 1+(\cos^2\theta-1)\cos^2\phi & (\cos^2\theta-1)\cos\phi\sin\phi & -\sin\theta\cos\phi \\ (\cos^2\theta-1)\cos\phi\sin\phi & 1+(\cos^2\theta-1)\sin^2\phi & -\sin\theta\sin\phi \\ \sin\theta\cos\phi & \sin\theta\sin\phi & \cos\theta \end{bmatrix}$$

Coordinate Transformations

In the focal plane, cylindrical coordinates are used for convenience:

$$x = r_2 \cdot \cos\phi_2 \tag{S11}$$

$$y = r_2 \cdot \sin\phi_2 \tag{S12}$$

Equation 3: Cylindrical Coordinates

Rewriting the electric field in cylindrical coordinates (r_2, ϕ_2, z):

$$\begin{aligned} \vec{E}(r_2,\phi_2,z) = -\frac{iC}{\lambda}\int_0^{\theta_{\max}}\int_0^{2\pi} \sin\theta\sqrt{\cos\theta}\cdot M\cdot \vec{E}_t \\ \times \exp\big[ik(\cos\theta\, z - r_2\sin\theta\cos(\phi-\phi_2))\big]\,d\phi\,d\theta \end{aligned} \tag{S13}$$

Equation 4: Simplified Expression

Finally, the simplified expression for practical use:

$$\begin{aligned} E(r_2,\phi_2,z) = -\frac{iC}{\lambda}\int_0^{\theta_{\max}}\int_0^{2\pi} \sin\theta\sqrt{\cos\theta}\cdot M\cdot E_t \\ \times \exp\big[ik\big(z\cos\theta + r_2\sin\theta\cos(\phi-\phi_2)\big)\big]\,d\phi\,d\theta \end{aligned} \tag{S14}$$

Explanation

- The integrals describe the electric field distribution in the focal region of a high numerical aperture (NA) lens, considering both scalar and vector diffraction effects.
- $\theta_{\max}$ limits the angular integration to the numerical aperture of the optical system.
- The matrix M accounts for the polarization and vectorial nature of light.
- Cylindrical coordinates (r_2, ϕ_2, z) simplify calculations in the imaging plane.

3 Two-dimensional Stokes Polarimetry of CPVBs

In Fig. S1, we employed comprehensive two-dimensional Stokes polarimetry to map the spatial polarization distribution of our cylindrical vector beams (CVBs), observing excellent correspondence with theoretical predictions. To model the theoretical focused field, we calculated the vectorial diffraction integral using the much faster Bluestein algorithm Hu et al (2020) rather than conventional direct integration or FFT-based approaches.

We use the following convention for out 2D stoke polarimetry characterization of the CPVBs:

$$I = |\hat{\mathbf{e}}_x|^2 + |\hat{\mathbf{e}}_y|^2, \tag{S15}$$

$$M = |\hat{\mathbf{e}}_x|^2 - |\hat{\mathbf{e}}_y|^2, \tag{S16}$$

$$C = |\hat{\mathbf{e}}_{45}|^2 - |\hat{\mathbf{e}}_{135}|^2, \tag{S17}$$

$$S = |\hat{\mathbf{e}}_r|^2 - |\hat{\mathbf{e}}_l|^2, \tag{S18}$$

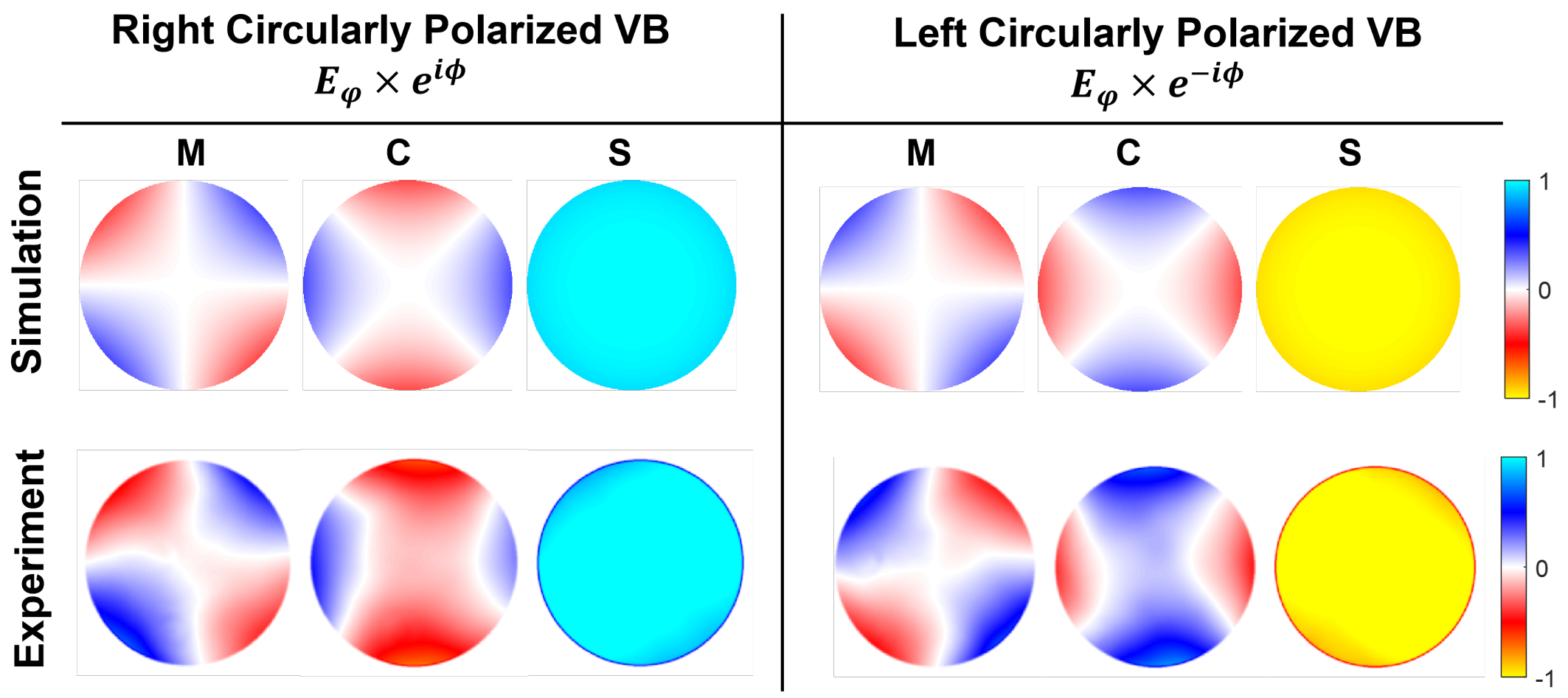

Fig. S1 2-D Stokes Polarimetry of CPVB at the focal plane.

A strong agreement is observed between the simulated and experimentally retrieved Stokes parameters. The parameter of primary interest is S, which dominates in the central high-intensity region. For a VPP with phase factor $e^{+i\phi}$, we obtain $S = +1$, corresponding to right-circular polarization $\hat{\mathbf{e}}_r$. Conversely, a VPP with phase factor $e^{-i\phi}$ yields $S = -1$, corresponding to left-circular polarization $\hat{\mathbf{e}}_l$.

4 Properties of CPVBs

Size comparison of VB and Gaussian beams at high-NA

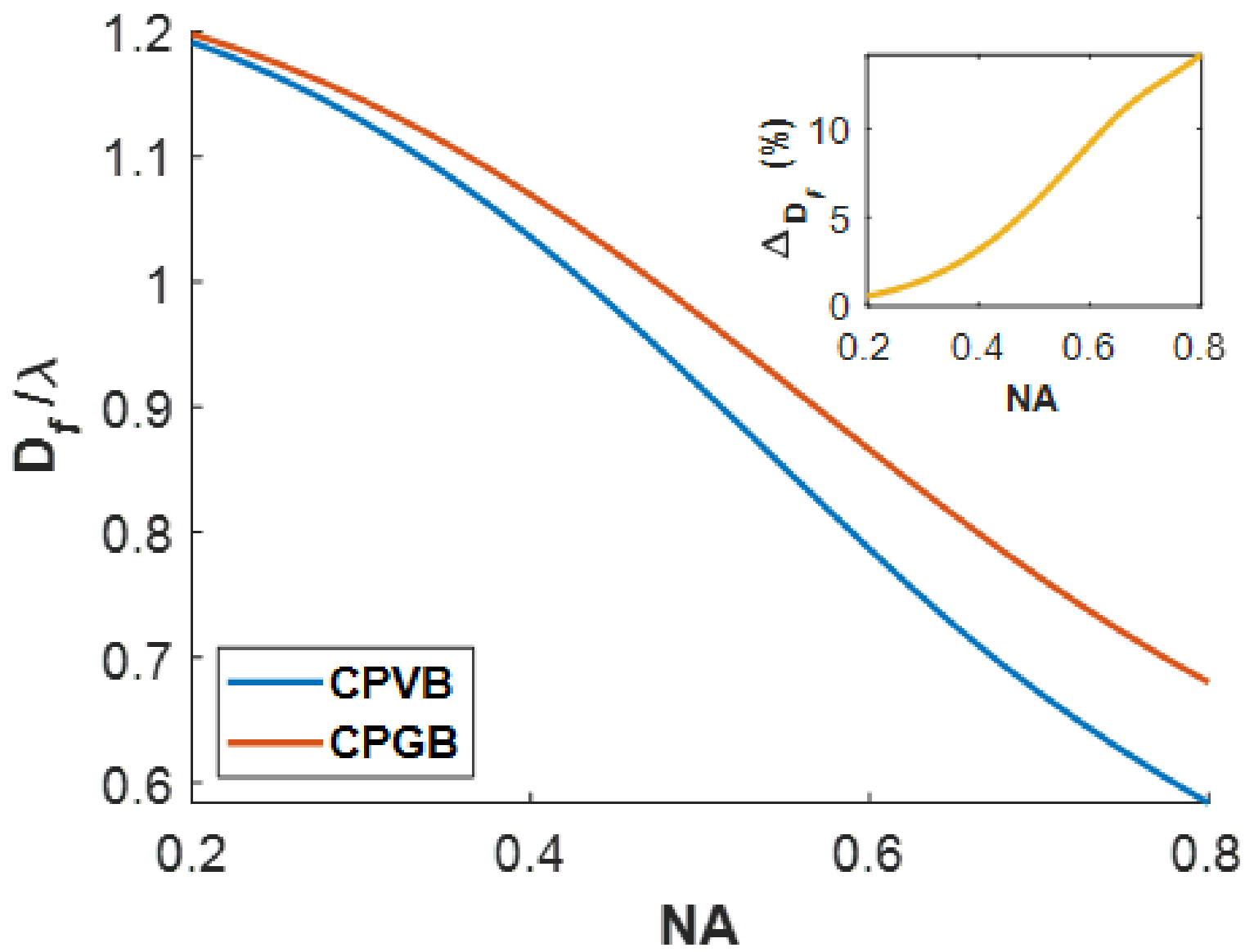

Fig. S2 Beam-width comparison between Gaussian beams and CPVBs as a function of numerical aperture (NA). The inset shows the percentage change in beam width with NA.

Axial evolution of different beams

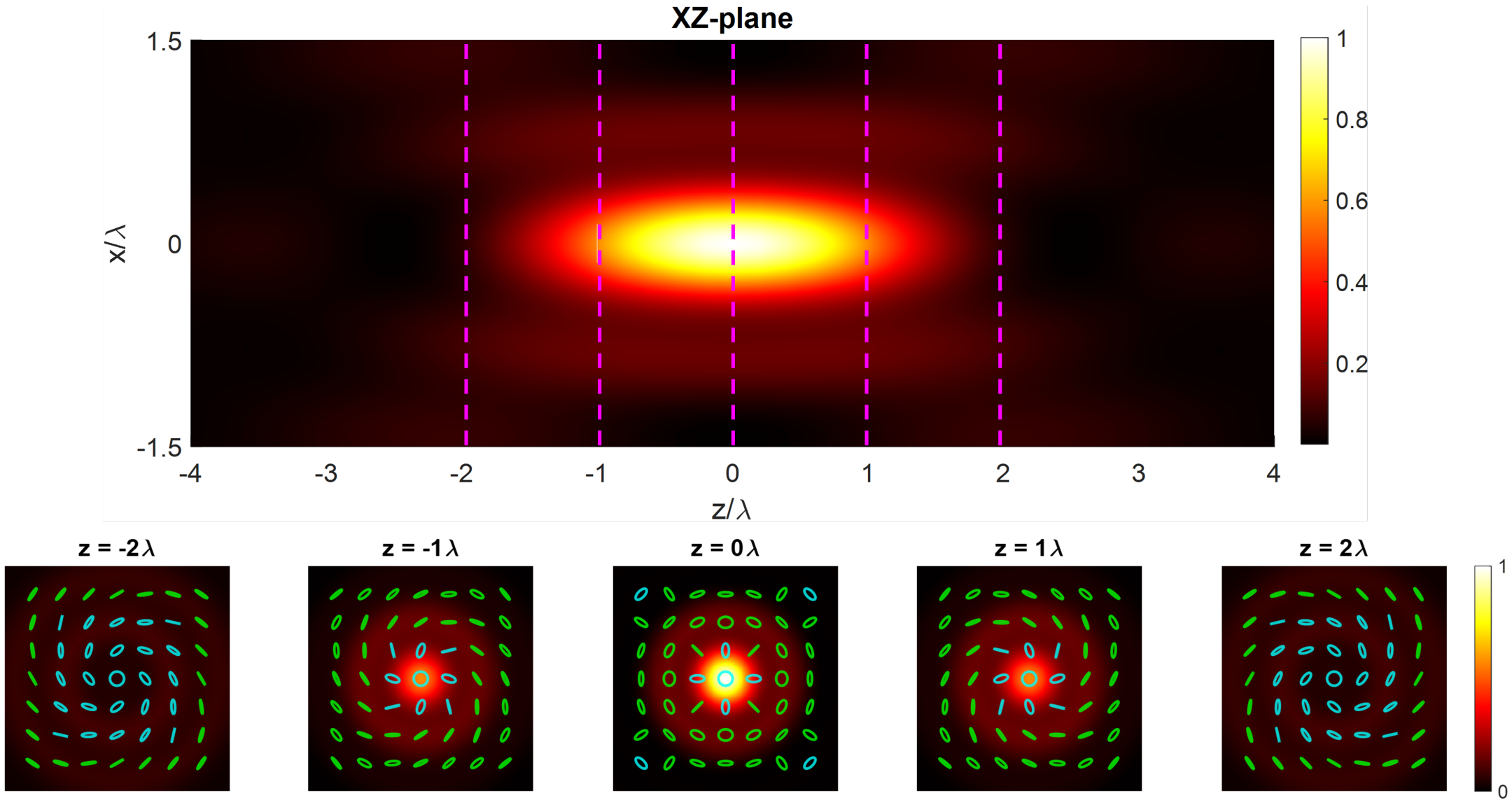

Fig. S3 Circularly polarized vector beam (CPVB) focused at NA = 0.40. Intensity and polarization ellipses show that, at the focal plane ($z = 0\lambda$), the field is nearly circular on axis and moving away from the high-intensity center into lower intensity regions, the polarization evolves from circular to elliptical. With axial displacement ($z = \pm 1\lambda$ and $z = \pm 2\lambda$), the high intensity region shifts off axis and its local polarization changes from circular to elliptical at the intensity maximum, indicating that the polarization state depends on the axial coordinate z and not solely on the transverse plane.

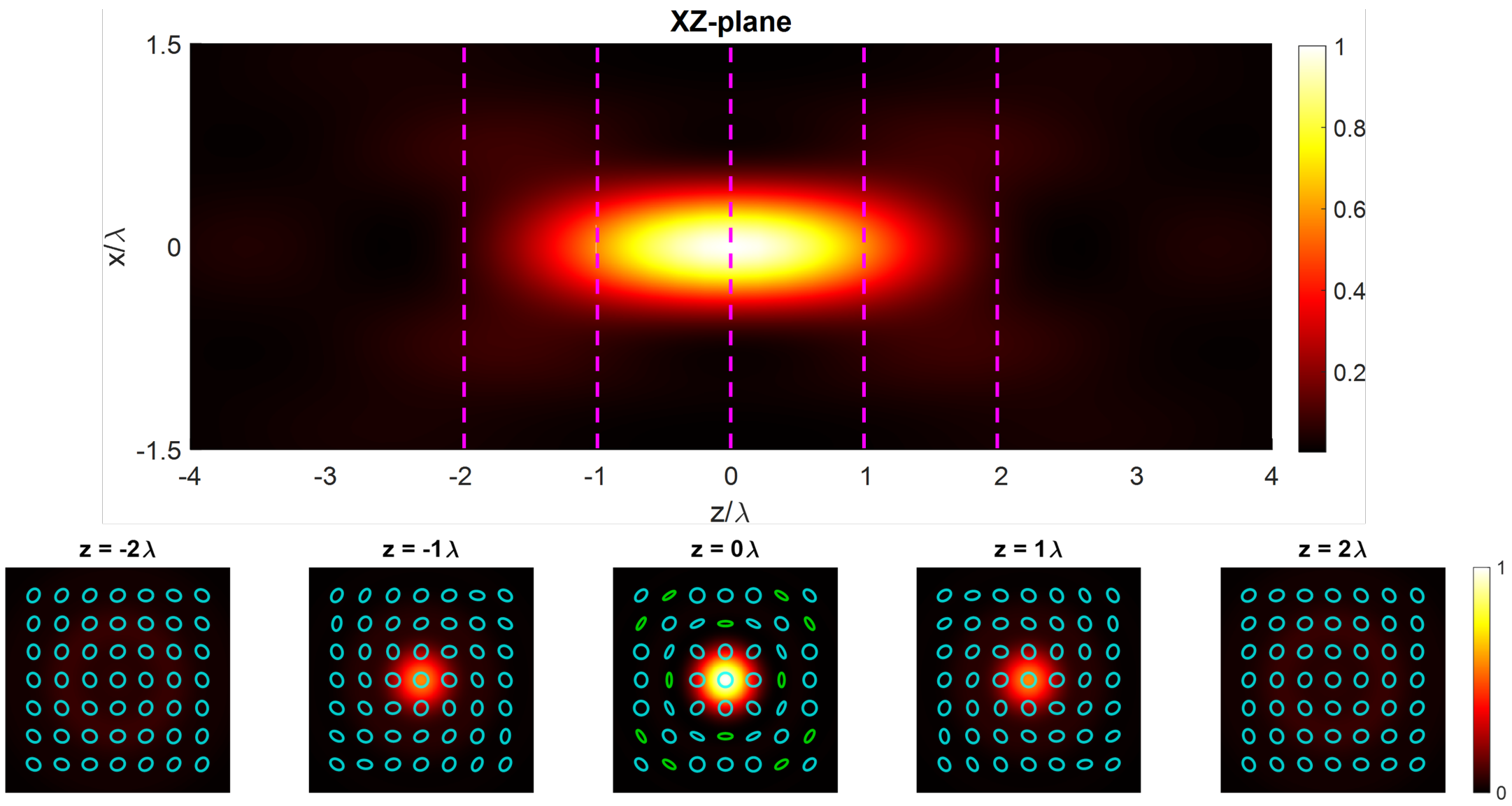

Fig. S4 Circularly polarized Gaussian beam (NA = 0.40). Intensity and polarization ellipses show that, at the focal plane ($z = 0\lambda$), the field is nearly circular on axis and across the high intensity region. Moving from the center into lower intensity regions, the polarization remains predominantly circular. With axial displacement ($z = \pm 1\lambda$ and $z = \pm 2\lambda$), the high-intensity region stays near axis and its local polarization remains close to circular at the high intensity regions.

For vector beams, when polarization is tailored at the focal plane, straying from this plane leads to decreased intensity and an undesirable distribution of spatial polarization. In comparison, a Gaussian beam deviates from the focal plane, it only experiences diminished intensity while preserving the polarization to deviations from the focus plane.

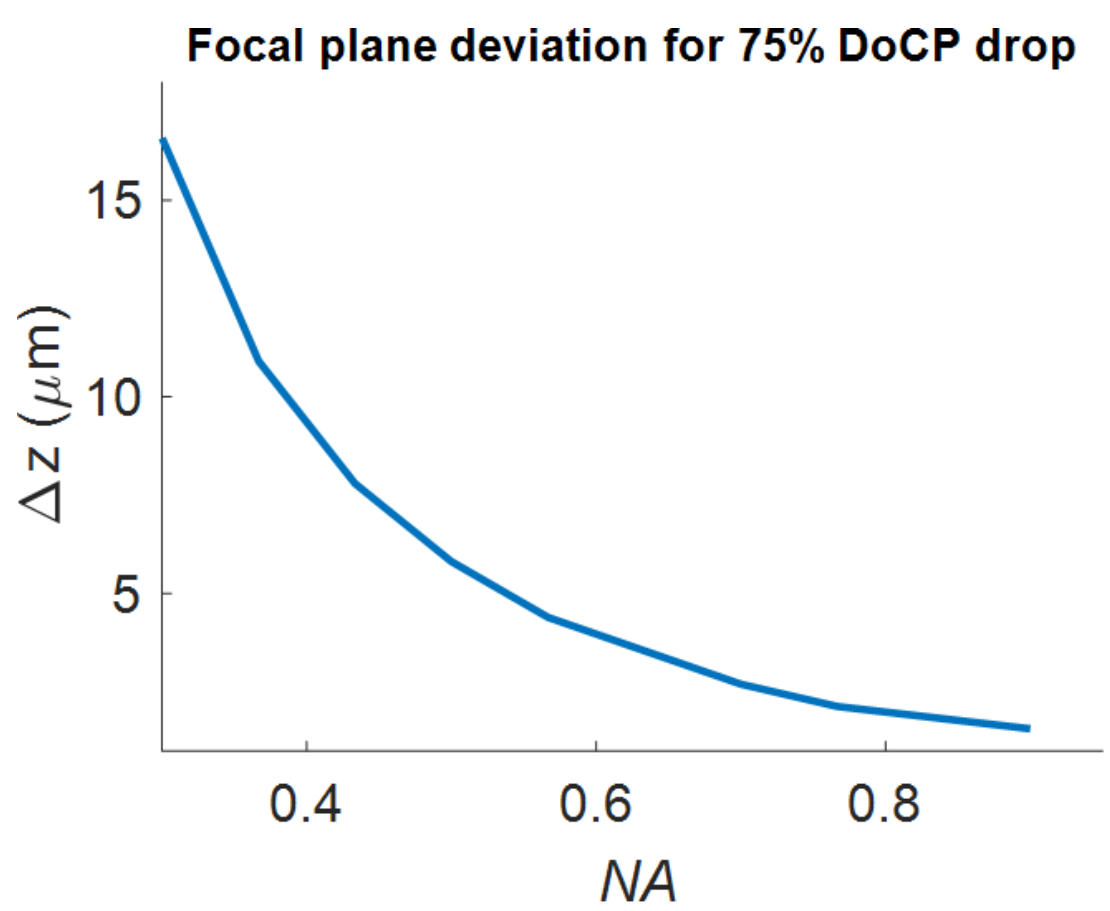

Fig. S5 Axial tolerance Δz within which the on-axis degree of circular polarization (DoCP) of a CPVB remains $\geq 75\%$ of its focal-plane value, plotted versus the objective's numerical aperture (NA). Higher NA lenses demand increasingly precise sample placement.

When the beam is precisely focused, a circularly polarized vector beam (CPVB) delivers the correct handedness and peak intensity for efficient all-optical switching (AOS). Any axial displacement $\left(z = f \pm z_d\right)$ simultaneously reduces the intensity *and* distorts the spatial polarization of a CPVB, raising the input power cannot recover the lost purity. In contrast, a Gaussian beams maintains its polarization off focus and merely loses power, so fluence can be restored, even though its inefficient, by increasing the input energy. This inherent "polarization filter" of CPVBs forces operation exactly at the imaging plane.

In Fig. S5, transforming the fields into the circular basis shows that the CPVB preserves at least 75 % of its on-axis, polarization-resolved intensity only within $|z - f| < z_d$. Beyond this, the Stokes parameter S falls below the threshold and the handedness reverses, suppressing AOS. Accurate sample placement at $z = f$ therefore guarantees minimal switching energy and the smallest achievable magnetic domain size, while avoiding the excess power tolerated by Gaussian beams.

5 Magnetic Material Analysis

Magnetic Field Along the Axis of a Uniformly Magnetized Thin Film

Consider a square thin film of side a, thickness t, and uniform out-of-plane magnetization M_s. We seek the z-component of the magnetic field, B_z, at a height z above the film's center.

Surface Magnetic Charge Approach

A uniformly magnetized film has effective surface magnetic charges $\sigma_m = \pm M_s$ at $z = \pm t/2$. The magnetic scalar potential at a height z above the film center due to a square sheet of surface charge is:

$$\phi_m(z) = \frac{\sigma_m}{2}\left(\sqrt{(a/2)^2 + (z - z_0)^2} - |z - z_0|\right)$$

where z_0 is the height of the sheet.

The total potential is the sum of contributions from the top ($z_0 = +t/2$) and bottom ($z_0 = -t/2$) surfaces:

$$\Phi(z) = \phi_{m,\text{top}}(z) + \phi_{m,\text{bottom}}(z)$$

The z-component of the magnetic field is then given by

$$B_z(z) = -\mu_0 \frac{\partial \Phi(z)}{\partial z}$$

Differentiating and combining terms yields:

$$B_z(z) = \frac{\mu_0}{2} M_s \left[\frac{z + t/2}{\sqrt{(z + t/2)^2 + (a/2)^2}} - \frac{z - t/2}{\sqrt{(z - t/2)^2 + (a/2)^2}} \right]$$

Special Case: Large, Thin Film

For $z \ll a$ and $t \ll a$, just above the film:

$$B_z(0^+) \approx \frac{\mu_0}{2} M_s$$

This formula gives the field along the symmetry axis above the film's center.

Analytical estimation of magnetic domain size

The domain size decreases systematically with increasing N, due to the increase in effective anisotropy and the resulting modification of the balance between domain wall and dipolar energies. To estimate the magnetic domain size of the investigated $[\mathrm{Co/Pt}]_N$ multilayers, we employed the analytical model developed by Kaplan Kaplan and Gehring (1993) for thin films with strong perpendicular uniaxial anisotropy. Within this framework, assuming negligible domain wall width compared to the stripe period and perpendicular magnetization within the domains, the equilibrium domain size D that minimizes the total energy is given by

$$D = t \exp\left[\frac{\pi D_0}{2t} + \ln(\pi) - 1 + \left(\frac{1}{2} - \ln 2 \right) \right] \tag{S19}$$

where

$$D_0 = \frac{\sigma}{E_d} \tag{S20}$$

and

$$\mu = 1 + \frac{E_d}{K_u}. \tag{S21}$$

Here t is the total magnetic thickness, $E_d = 2\pi M_s^2$ is the demagnetizing energy density, and $\sigma = 4\sqrt{A_{\mathrm{ex}} K_u}$ is the domain wall energy per unit area. A_{ex} is the exchange stiffness and K_u (equivalent here to K_{eff}) is the effective perpendicular anisotropy constant.

In our calculations we only consider the thickness of the Co layer since it is the magnetic material in the multilayer system. The saturation magnetization M_s was measured directly using a vibrating sample magnetometer (VSM). The effective anisotropy K_u and exchange

stiffness A_{ex} were estimated using literature values reported for comparable Co/Pt multilayer systems Eyrich et al (2012).

Using these parameters, we obtain the equilibrium domain sizes summarized in Table 1.

N	t_{Co} (nm)	A_{ex} (10^{-6} erg/cm)	M_s (emu/cm^3)	K_u (10^7 erg/cm^3)	D (μm)
1	0.6	1.2	1406	0.1392	2688
3	1.8	1.2	1566	0.9728	182.6
5	3.0	1.2	1620	1.3468	7.5
7	4.2	1.2	1592	1.3253	1.6
9	5.4	1.2	1608	1.6124	0.9

Supplementary Table 1 Calculated equilibrium magnetic domain sizes for $[Co/Pt]_N$ multilayers using the Kaplan analytical model with experimentally measured and literature-based parameters.

6 Role of laser sweeping speeds and beam size on AOS

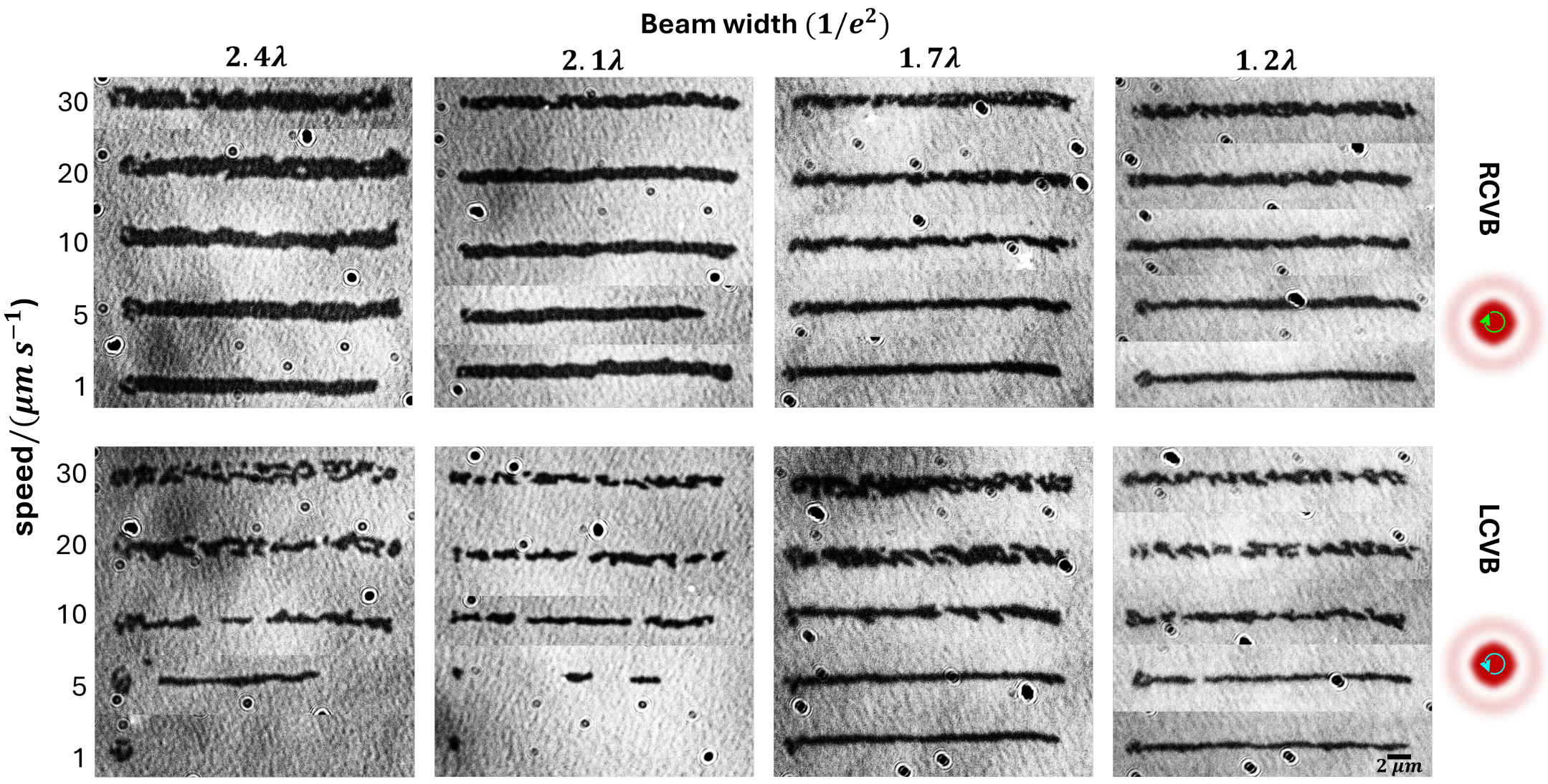

Fig. S6 AOS in Pt/Co (N = 3) thin films as a function of laser scanning speed and beam width for $\mathbf{M}^+$. **Top row:** Right Circularly Polarized Vector Beam (RCVB); **Bottom row:** Left Circularly Polarized Vector Beam (LCVB). Each column represents progressively decreasing focused beam widths from 2.4λ to 1.2λ. The cumulative number of laser pulses, governed by the scanning speed, dictates whether the system undergoes deterministic magnetization reversal. At slower speeds, pulse accumulation facilitates switching, while at higher speeds, insufficient exposure prevents it

To probe the deterministic control of AOS in Pt/Co multilayers, we systematically varied both the laser scanning speed and the focused beam width. As shown in Fig. S7, the interplay between these parameters governs the cumulative effect of laser pulses and, consequently, the switching dynamics. The figure presents a clear comparison: RCVB and LCVB switching attempts are displayed in the top and bottom rows, respectively, across beam widths 2.4λ, 2.1λ, 1.7λ and 1.2λ. The laser scanning speeds investigated were 30, 20, 10, 5, and 1 μm/s, enabling

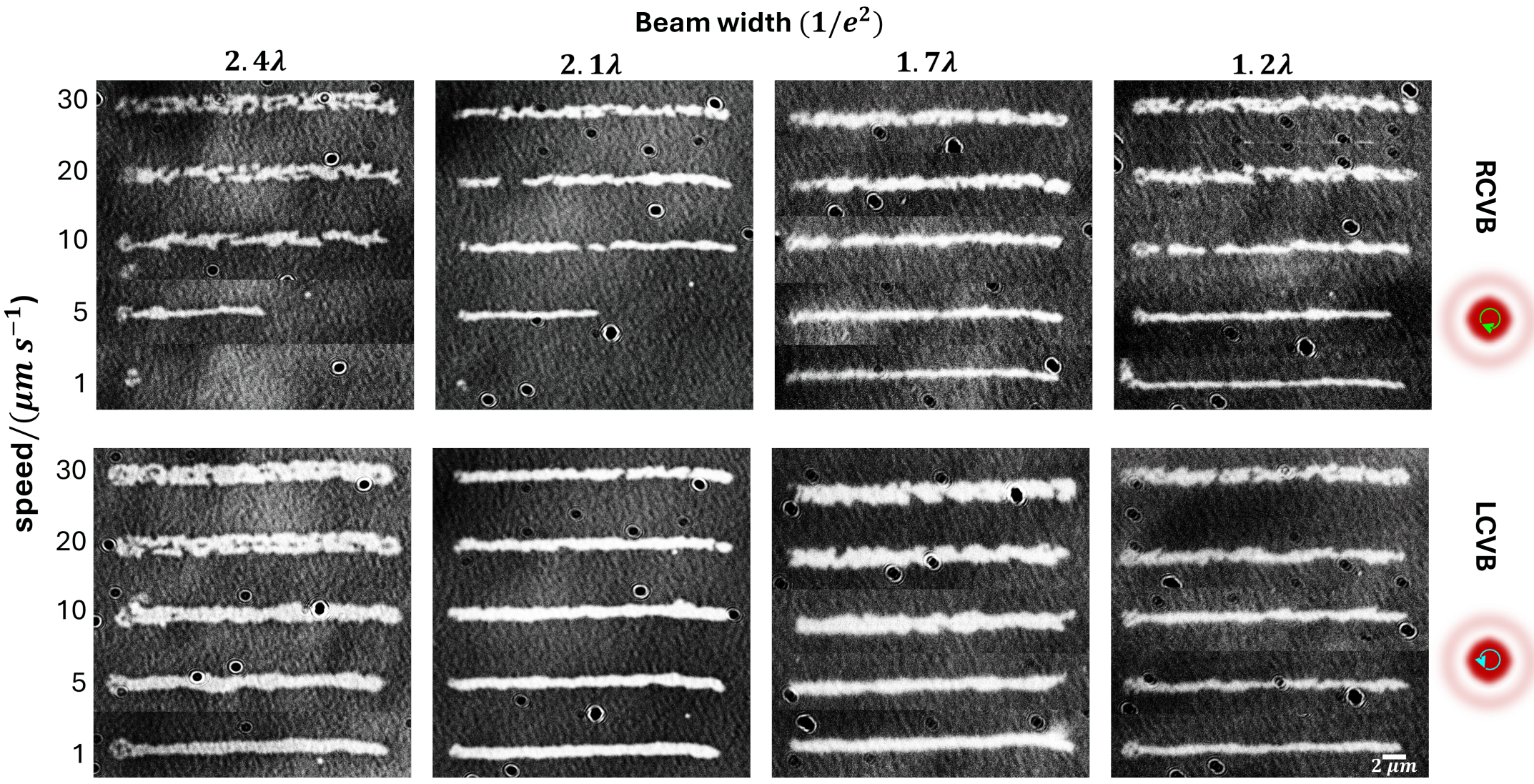

Fig. S7 AOS in Pt/Co ($N = 3$) thin films as a function of laser scanning speed and beam width for $\mathbf{M}^-$. **Top row:** Right Circular Vector Beam (RCVB); **Bottom row:** Left Circular Vector Beam (LCVB).

control over the cumulative number of pulses delivered to a given region. Since these panels are composites of images acquired at different regions, but with the same initial magnetization state, the apparent contrast may vary slightly between sub-frames.

At slower speeds (e.g., 1 μm/s), the high pulse accumulation facilitates deterministic switching, while at higher speeds (e.g., 30 μm/s), the reduced exposure hinders the switching process nad the system remain in an nucleation state. For larger beam widths (2.4λ and 2.1λ), the magnetization outcome is dictated by the beam's helicity, as it is commonly observed in $N = 1$ and $N = 2$. However, at smaller beam widths, this dependence disappears. This transition is critically linked to the multilayer thickness and we see this to start at the $N = 3$ onward $[\mathrm{Pt/Co}]_N$ bilayers. In Fig. S6 and S7, the apparent asymmetry between RCVB and LCVB at higher scanning speeds for N=3 arises from stage-limited pulse overlap combined with the transitional nature of this multilayer, which marks the crossover from helicity-dependent to helicity-independent switching. In these films, the strong magnetostatic dipolar fields from the surrounding, unheated film dominate the final state of the localized laser-affected region, effectively overriding the helicity information. This behavior highlights a unique switching regime that emerges only in thicker multilayers. In contrast, thinner stacks (e.g., $N = 1$ or $N = 2$) lack the requisite dipolar strength, and Magnetic Circular Dichroism (MCD)-driven helicity-dependent switching persists even at small spot sizes.

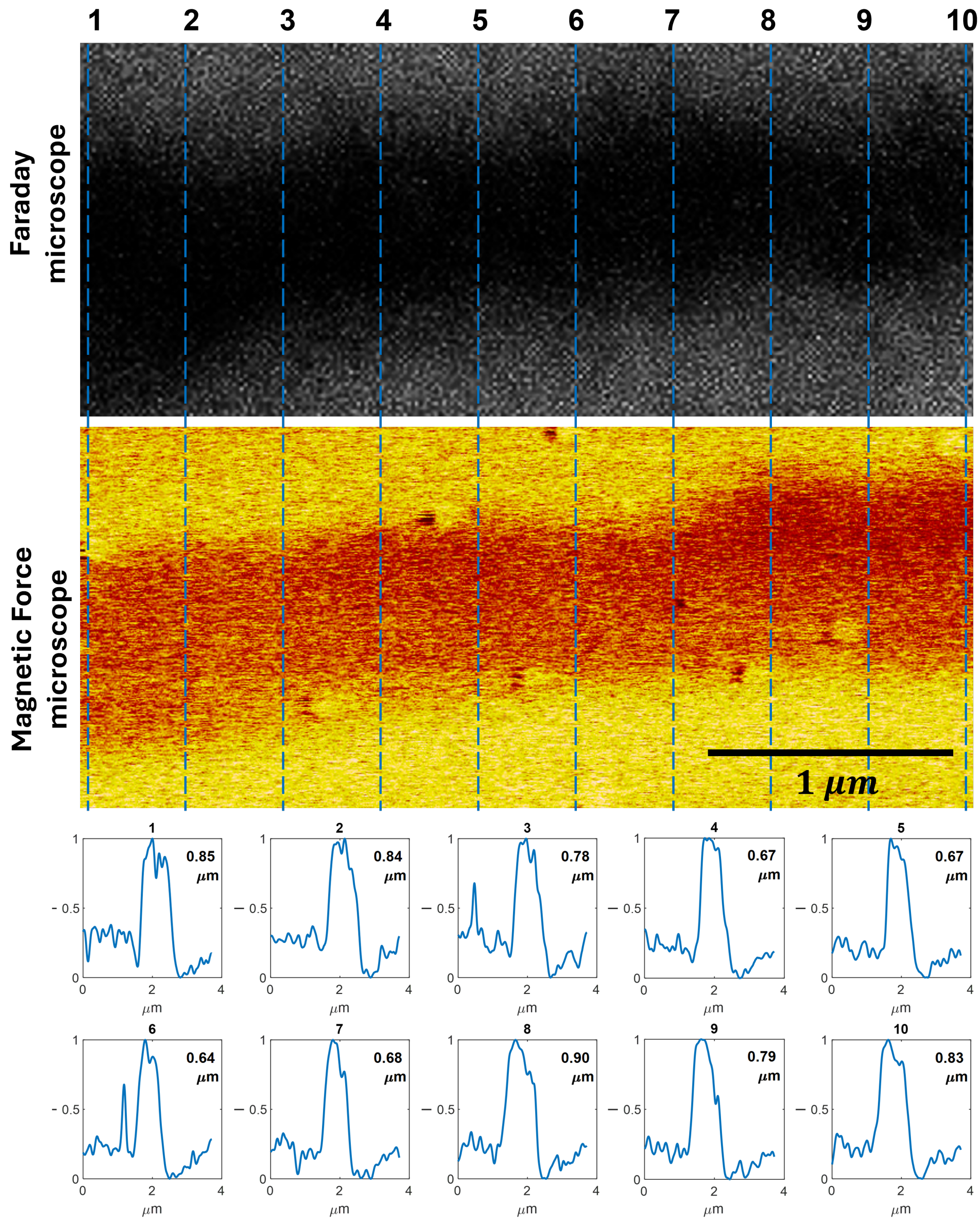

Fig. S8 Faraday microscopy and magnetic force microscopy (MFM) images of the switched region in the (N = 3) sample. The MFM image, with higher spatial resolution and magnetic contrast, is used to quantify the lateral switching width. Ten equally spaced line cuts were extracted across the switched area, and the corresponding full width at half maximum (FWHM) values are displayed in the accompanying subplot; the top right corner of each line-cut graph indicates the extracted FWHM.

7 Magnetic Force Microscopy Imaging of N = 3

Fig. S8 presents representative Faraday microscopy and magnetic force microscopy (MFM) images of the same region of the ($N = 3$) sample, enabling a direct comparison between the magnetic features observed with both techniques. While the Faraday image qualitatively confirms magnetization reversal, its limited spatial resolution and contrast prevent accurate

determination of the switching width. In contrast, the MFM image provides substantially higher spatial resolution and magnetic contrast, enabling precise quantification of the lateral size of the switched region.

In the present manuscript, the primary focus is on the development and validation of the optical switching technique and on demonstrating the switching behavior observable with Faraday imaging within the resolution limits of the microscope. Having established the effectiveness of the technique, future investigations can explore magnetic switching of nanoscale magnetic domains using tighter (smaller) optical beams.

To determine the switching width, ten equally spaced line cuts were extracted from the MFM image across the switched area, and the corresponding full width at half maximum (FWHM) values are shown in the accompanying subplot. The measured widths range from $\sim 0.67\,\mu$m to $\sim 1.04\,\mu$m, yielding an average switched width of $\sim 0.76\,\mu$m $\pm$ 0.09. These measurements confirm sub-micron switching in the $[\text{Pt/Co}]_N$ ($N = 3$) sample, resolved beyond the diffraction limit of optical microscopy.

8 Higher order stacks

$[Pt/Co]_4$

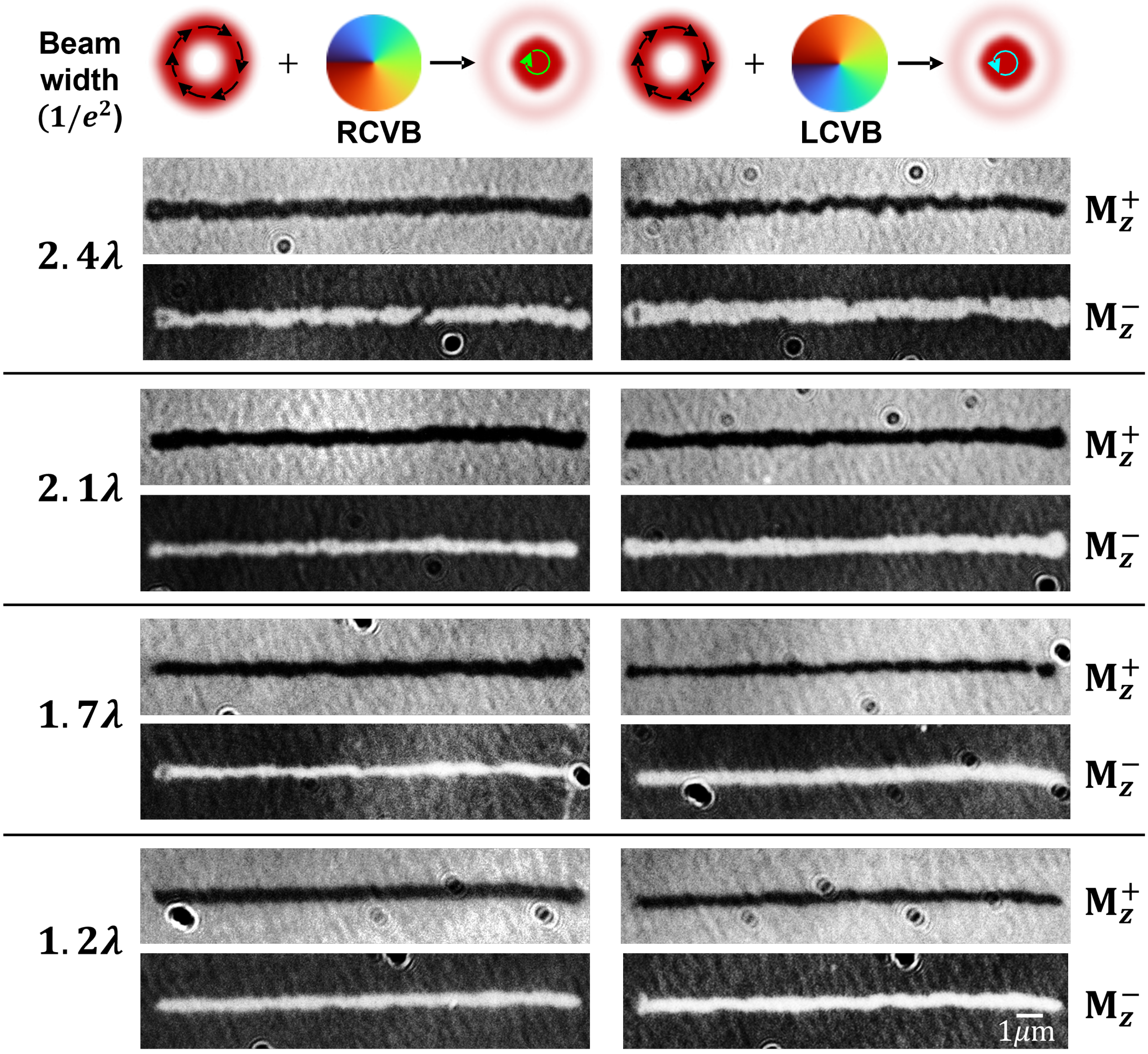

Fig. S9 $N = 4$ sweeps at different beam sizes. Switching outcomes are shown for a $[Pt/Co]_4$ multilayer with $N = 4$ under illumination by a right-circular vector beam (RCVB) and left-circular vector beam (LCVB). Results are displayed for progressively reduced effective spot sizes. Across all beam sizes tested, the reversal is helicity independent (AO–HIS), which reflects strong dipolar stabilization of the laser-affected region, overriding the magnetic circular dichroism bias.

195 **$[Pt/Co]_{10}$**

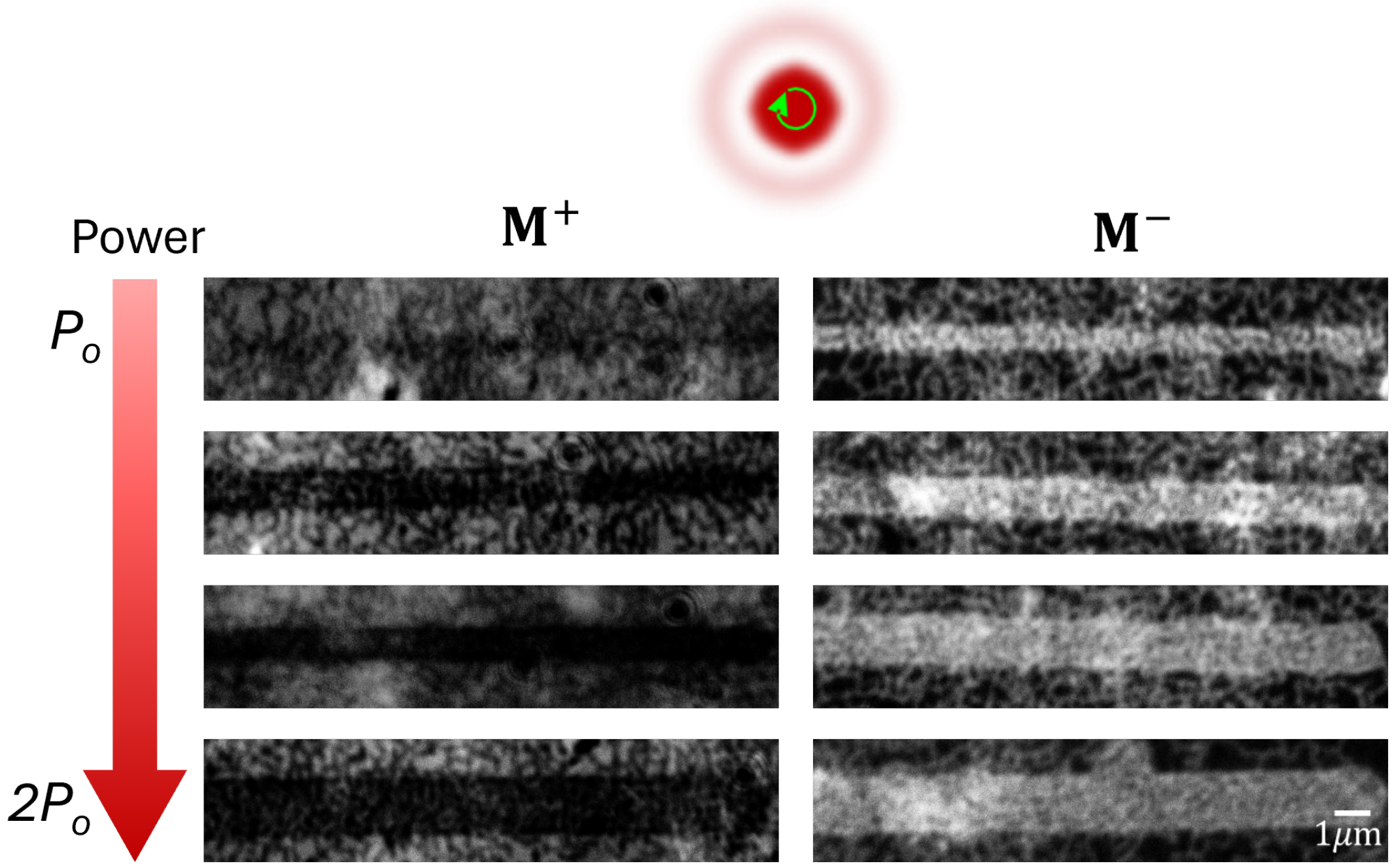

Fig. S10 $\boldsymbol{N} = 10$ sweeps at different average powers. The left column shows results with an initial magnetization state of $\mathbf{M}_z^+$, and the right column with $\mathbf{M}_z^-$. A right-circular vector beam (RCVB) is used as the incident beam. For $\boldsymbol{N} = 10$, only partial switching is observed, attributed to stripe-domain formation.

9 Single-shot laser response

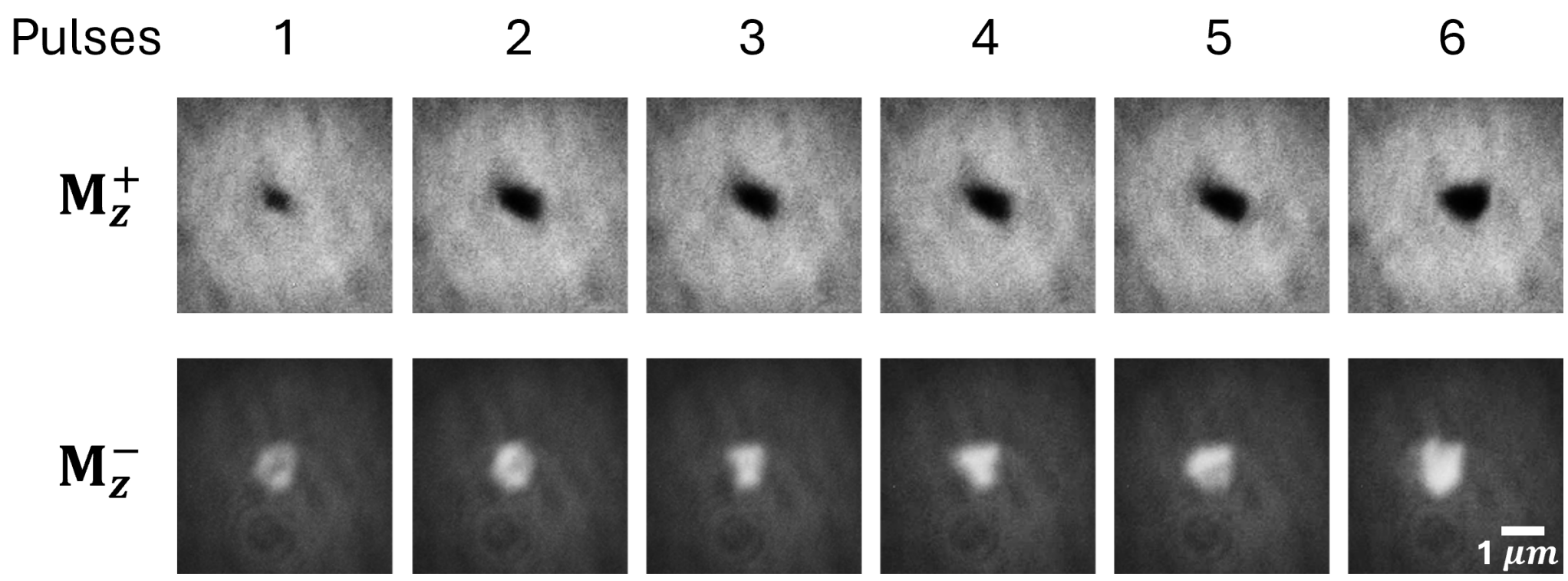

Fig. S11 For $\boldsymbol{N} = 4$, sequential single-pulse exposures of RCVBs show monotonic growth of the switched domain with no reversal of its polarity. Outside the switched region, the magnetization remains unchanged. The absence of toggle switching is consistent with dipolar fields stabilizing the reversed domain.

10 Energy Dynamics

At a scanning speed of 5 μm s^{-1}, a laser repetition rate of 1 kHz, and a spot size of 1 μm, approximately 200 laser pulses are delivered within a 1 μm^2 area, corresponding to the effective laser spot size.

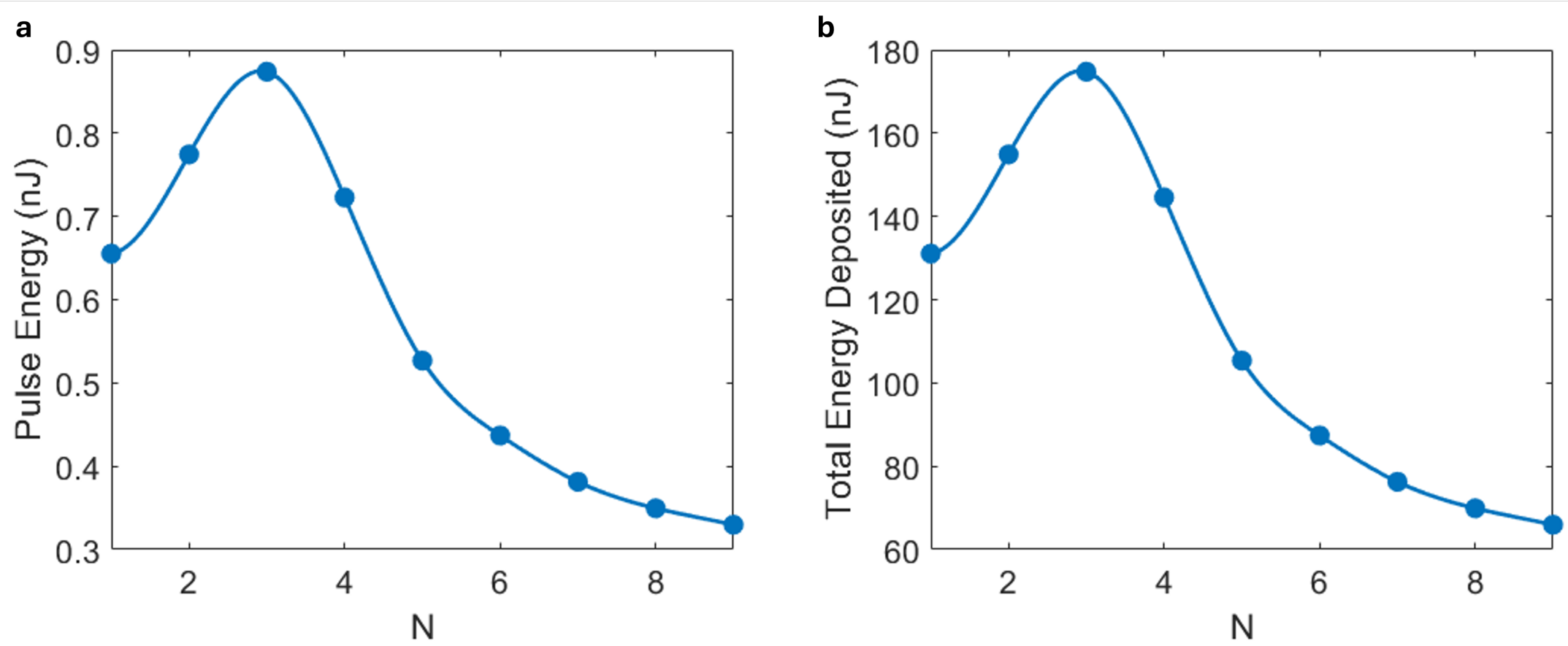

Fig. S12 (a) Single-pulse (b) Total energy required to achieve all-optical switching (AOS) for multilayers with N = 1–9)

11 Gaussian beam control experiment

The Gaussian-beam control experiment was performed by removing the vector-beam-generating optics and generating right- or left-circular polarization using a quarter-wave plate

(QWP) oriented at $+45°$ or $-45°$, respectively, with respect to the polarizer. Below is the modified setup used for the performed experiments:

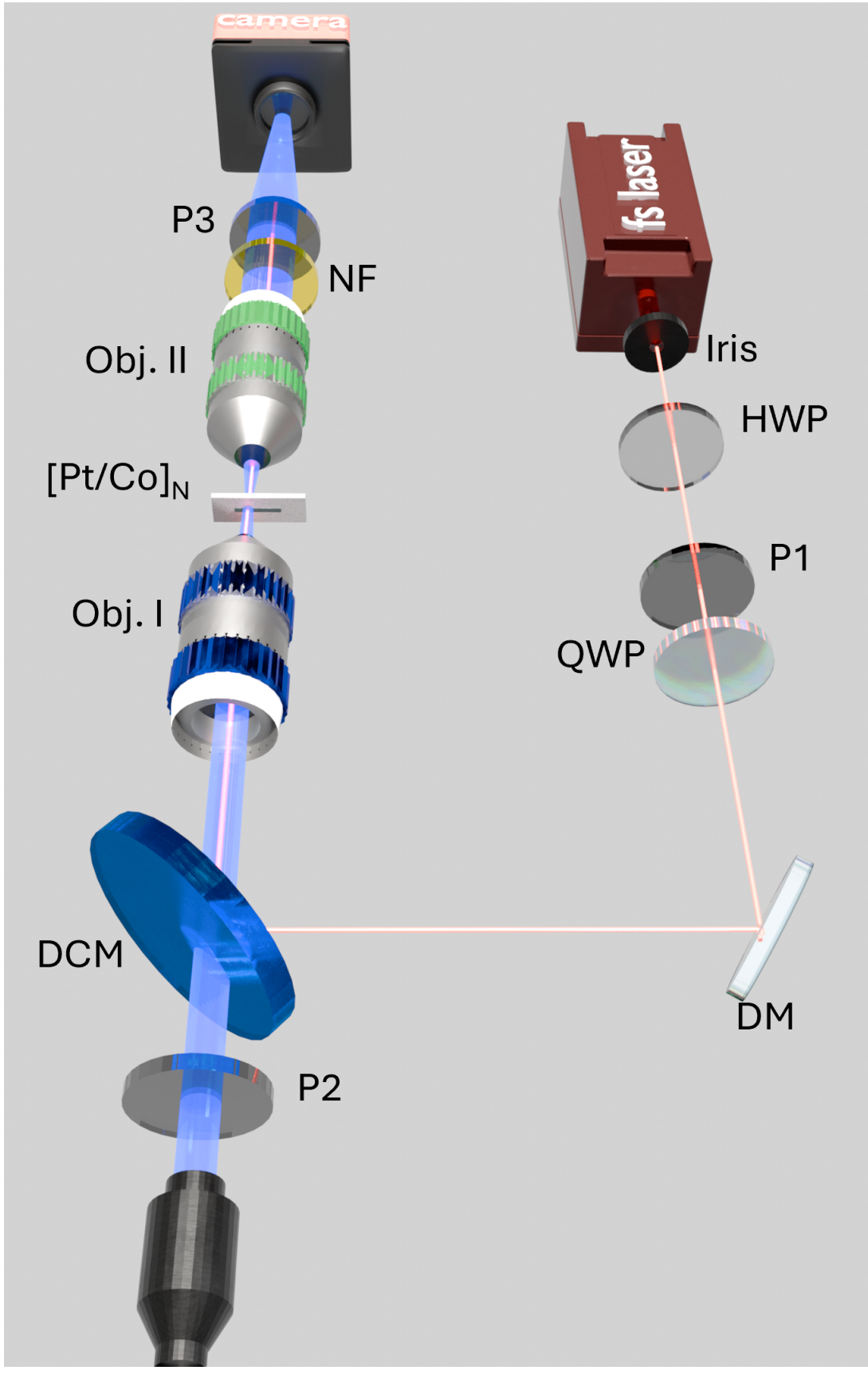

Fig. S13 Optical setup used for circularly polarized Gaussian beams. A linearly polarized femtosecond (fs) laser beam is trimmed using an *iris*. A half-wave plate (HWP) and polarizer ($P1$) are used to control the optical power and set the reference polarization. Circularly polarized Gaussian beams are generated using a quarter-wave plate (QWP). The fs pulses are reflected by a dielectric mirror (DM) and combined with LED illumination at a dichroic mirror (DCM). The LED light and fs pulses are focused onto the sample using *Obj I*, while the sample image is relayed to the camera through *Obj II* and an 800 nm notch filter (NF), which blocks the fs pulses. A Faraday microscope is formed using polarizer *P2* and analyzer *P3*, providing magnetization contrast.

We also performed a direct comparison using a conventional circularly polarized Gaussian beam under experimental conditions comparable to those used for the vector-beam measurements, including similar focusing, optical power, and polarization state, as shown in Figs. S14 and S15. Under these conditions, the Gaussian beam did not reproduce the deterministic switching observed with the vector beam. For N = 3-5, Gaussian-beam exposure resulted in permanent sample damage. For N = 6-9, it produced non-deterministic magnetic domain formation, characterized by multiple randomly nucleated domains rather than a well-defined switched region. The evolution of these domains with increasing N is consistent with the larger

magnetic volume and the lower optical energy required to induce magnetic changes in thicker multilayers.

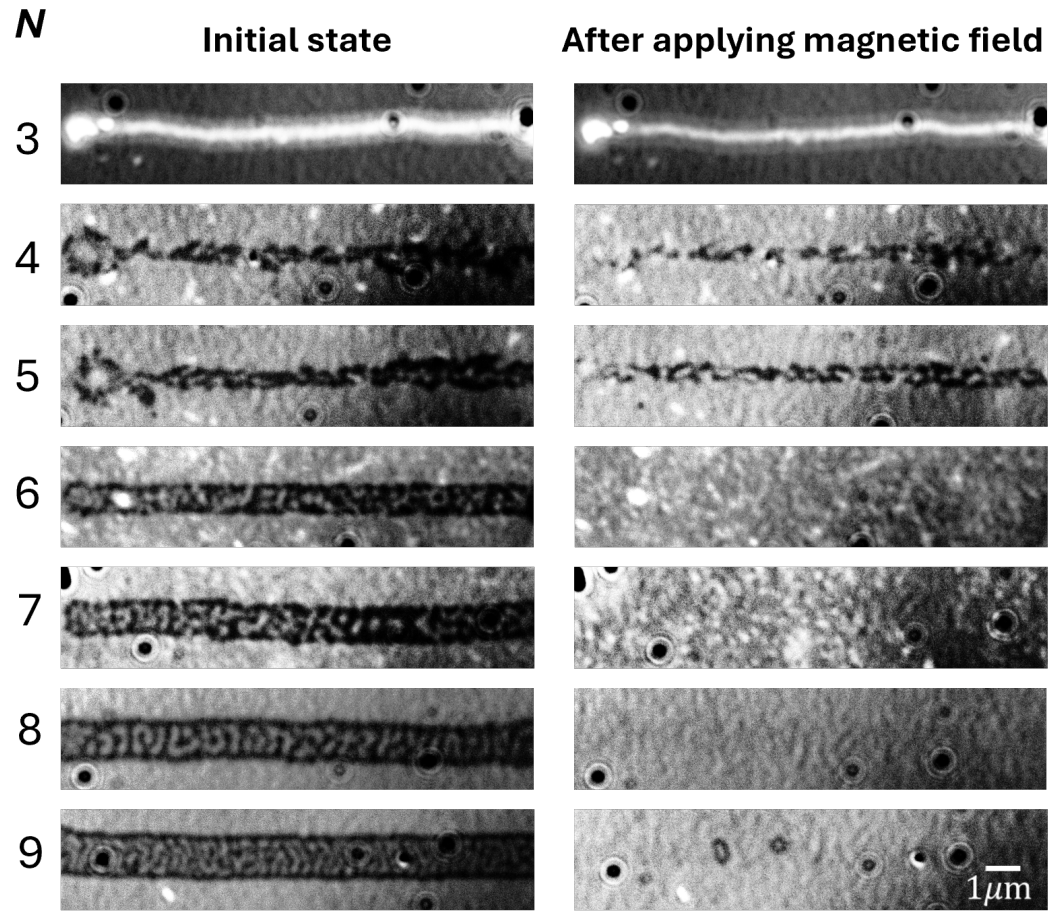

Fig. S14 Conventional circularly polarized Gaussian-beam exposure for an initial magnetization state of M^+ and incident RCP polarization. For $N = 3$, 4, and 5, the exposure caused permanent sample damage. For $N = 6$, 7, 8, and 9, the laser pulses produced non-deterministic magnetic domain formation, with multiple randomly nucleated domains instead of a well-defined switched region. The first column shows the magnetic state after exposure to femtosecond laser pulses, while the second column shows the same regions after applying an external magnetic field strong enough to saturate the thin film back to its pre-switched magnetization direction.

To distinguish reversible magnetic switching from permanent optical modification, we further performed an external magnetic-field erasure test, which is essential for confirming all-optical switching in these thin films. After optical exposure, a permanent magnet was brought near the sample to reset the magnetization to its initial state. The regions exposed to the Gaussian beam under comparable conditions did not exhibit the same reversible, well-defined response observed for the vector beam. In particular, for N = 3-5, the optically modified regions could not be erased by the external magnetic field, confirming that the observed contrast arose from permanent sample damage rather than reversible magnetization switching.

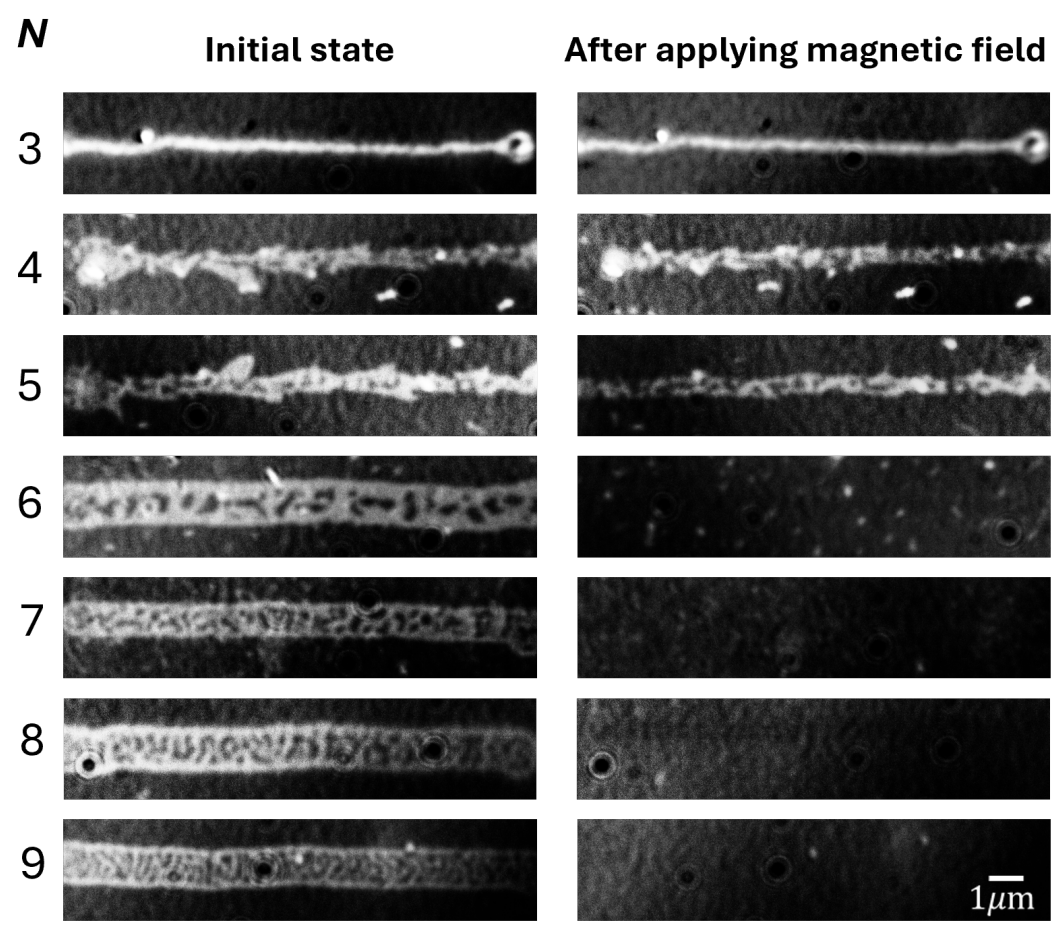

Fig. S15 Conventional circularly polarized Gaussian-beam exposure for an initial magnetization state of M^- and incident RCP polarization. For $N = 3$, 4, and 5, the exposure caused permanent sample damage. For $N = 6$, 7, 8, and 9, the laser pulses produced non-deterministic magnetic domain formation, with multiple randomly nucleated domains instead of a well-defined switched region. The first column shows the magnetic state after exposure to femtosecond laser pulses, while the second column shows the same regions after applying an external magnetic field strong enough to saturate the thin film back to its pre-switched magnetization direction.

References

Eyrich C, Huttema W, Arora M, et al (2012) Exchange stiffness in thin film co alloys. Journal of Applied Physics 111(7)

Hu Y, Wang Z, Wang X, et al (2020) Efficient full-path optical calculation of scalar and vector diffraction using the bluestein method. Light: Science & Applications 9(1):119

Kaplan B, Gehring G (1993) The domain structure in ultrathin magnetic films. Journal of Magnetism and Magnetic Materials 128(1-2):111–116

Richards B, Wolf E (1959) Electromagnetic diffraction in optical systems, ii. structure of the image field in an aplanatic system. Proceedings of the Royal Society of London Series A Mathematical and Physical Sciences 253(1274):358–379
