# Supplementary material for: Optical beam shaping induced reconfigurable magnetic domains
Source: Nat Commun. 2026 Sep 15;17:10843. doi: 10.1038/s41467-026-77572-3 (PMC13672297; doi:10.1038/s41467-026-77572-3)
Supplement: Supplementary file 2 [file 41467_2026_77572_MOESM2_ESM.pdf]

nature portfolio

Peer Review File

Optical beam shaping induced reconfigurable magnetic domains

Corresponding Author: Professor Abdoulaye Ndao

This file contains all reviewer reports in order by version, followed by all author rebuttals in order by version.

Attachments originally included by the reviewers as part of their assessment can be found at the end of this file.

Version 0:

Reviewer comments:

Reviewer #1

(Remarks to the Author)

Reviewer #2

(Remarks to the Author)
The manuscript presents an interesting experimental study of all-optical switching in Pt/Co multilayers using circularly polarized vector beams under high-NA focusing. The data are extensive and internally consistent. The work addresses an important question in ultrafast magnetism and spintronics, and thus has a potential to be considered for Nature Communications. However, while the results are promising and largely convincing, I have some major conceptual questions which I hope the authors can address.

Major Questions

1. The manuscript argues that vector beams outperform Gaussian beams due to reduced longitudinal field components and slightly tighter confinement at fixed NA. However, the presented results primarily correlate the transition to helicity-independent switching with reaching wavelength-scale spot sizes. This is not new in fact. As a consequence, it remains unclear whether beam shaping introduces qualitatively new physics or mainly provides an efficient route to this confinement regime. Can the vector field help if the size of the laser spot is larger?

2. Suppression of the longitudinal electric-field component is highlighted as a key advantage of vector beams at high NA. It would be useful to clarify whether non-negligible longitudinal fields are expected to directly hinder deterministic switching in Pt/Co multilayers, or whether lateral energy confinement and thermal gradients alone are sufficient to explain the observed behaviour.

3. The authors state that vector beams reduce the power required for deterministic switching, but the effect on the intrinsic fluence threshold is not clearly established. Given that the switching is multi-pulse and thermally driven, it would interesting to know whether beam shaping modifies the threshold physics or primarily rescales the local energy density.

4. The authors refer to nanoscale a lot but I see that switching still occurs on the micron scale. This can be misleading.

Minor Comments

-To avoid confusion with single-pulse reports the multi-pulse nature of the presented helicity-independent switching should be emphasized earlier .
-The definition of effective spot size should be stated explicitly.
-I suggest providing spot sizes in Figures 3 and 4.

Reviewer #3

(Remarks to the Author)
attached

Version 1:

Reviewer comments:

Reviewer #2

(Remarks to the Author)
I thank the authors for their detailed responses and for the improvements made to the manuscript.

The authors provided an extensive explanation of the differences in switching behavior between tightly focused vector beams and Gaussian beams. However, the central conclusions are currently supported only by simulations which they also include in the manuscript. Since this is an experimental work, I believe the key claim can and should be verified experimentally in a straightforward manner.

In particular, the experiment proposed schematically in Fig. 3a can serve as a direct “smoking-gun” test. The authors should first demonstrate switching using the tightly focused vector beam generated with the SVV. Then, under otherwise identical experimental conditions (same laser power, focusing conditions, and polarization state), they should simply remove the SVV element and compare the switching behavior for a conventional but tighly focused circularly polarized Gaussian beam.

Such a comparison would directly demonstrate whether the vectorial nature of the beam is indeed essential for the observed switching. For example, the Gaussian beam may fail to switch, require substantially different conditions, or produce qualitatively different behavior. Any of these outcomes would help to clarify role the vectrorial fields.

Without this experimental verification, I unfortunately cannot recommend publication at this stage, because simulations alone are not sufficient to substantiate such a central and interesting claim. However, if these measurements are performed and the results clearly support the proposed mechanism, I would have no objection to publication of this interesting work in Nature Communications.

I also encourage the authors to include these measurements either in the main text or in the Supplementary Information.

Reviewer #3

(Remarks to the Author)
I am satisfied with the author's response and the revised manuscript. I recommend the publication of the modified version in Nature Communications.

Version 2:

Reviewer comments:

Reviewer #2

(Remarks to the Author)
I thank the authors for performing the additional measurements. The new data clearly demonstrate that heating induced by OAM pulses (N=3-9) is reduced compared to conventional circularly polarized excitation. This is an important result that strengthens the manuscript.

It would nevertheless be interesting to investigate the helicity-dependent switching response under OAM excitation in order to clarify the role of orbital angular momentum in the optical torque responsible for switching. Unfortunately, such measurements have not been performed on samples 1 and 2 (N=1-2), and therefore this question remains open. I hope the authors will be able to address this intriguing aspect in future studies.

Nevertheless, I believe that the authors have satisfactorily addressed the concerns raised during the review process, and I support publication of the manuscript in Nature Communications without further delay.

Open Access This Peer Review File is licensed under a Creative Commons Attribution 4.0 International License, which permits use, sharing, adaptation, distribution and reproduction in any medium or format, as long as you give appropriate credit to the original author(s) and the source, provide a link to the Creative Commons license, and indicate if changes were made.
In cases where reviewers are anonymous, credit should be given to 'Anonymous Referee' and the source.
The images or other third party material in this Peer Review File are included in the article's Creative Commons license, unless indicated otherwise in a credit line to the material. If material is not included in the article's Creative Commons license and your intended use is not permitted by statutory regulation or exceeds the permitted use, you will need to obtain permission directly from the copyright holder.
To view a copy of this license, visit https://creativecommons.org/licenses/by/4.0/

In the manuscript titled ***"*Beam shaping induced reconfigurable magnetic domains,"** authored by Muhammad Waleed Khalid et al., the authors presented their work on deterministic control of all-optical switching through optical engineering. They have obtained both helicity-dependent and helicity-independent magnetization switching $[Co/Pt]_N$ multilayer using polarization manipulation of the optical pulse, without material modification or external bias. The work is novel in the present context of spintronics research, and I recommend publication of this manuscript in Nature Communication after the following modifications.

1. The authors discussed deterministic switching with respect to the domain size in $[Co/Pt]_N$ multilayers. What is the typical domain size of the $[Co/Pt]_N$ multilayer system under investigation, and how does the domain size change with N?

2. The authors discussed the effect of E_z in Gaussian circularly polarized beams and circularly polarized vector beams in detail. I would like to see the temperature profiles (laser-induced heating) of the samples under investigation for both Gaussian and Vector beam excitation.

3. AO-HIS in these samples still requires a large number of pulses. Roughly, how many pulses are required to observe AO-HIS? Apart from mentioning the beam widths, the authors should also report the pulse energy and compare it with the switching energy for single-shot AO-HIS observed in typical systems. Can we deliver such pulse energy using a fibre laser compatible with on-chip photonics?

4. Why don't the $[Co/Pt]_N$ sample show single-shot toggle-type AO-HIS? (like in GdFeCo alloys and exchange-coupled $[Co/Pt]_N$ multilayers?). Authors should include a discussion in the revised manuscript.

5. The authors start from M+ state, use RCVB to show HI-AOS. Then, if they use LCVB in the same switched region (at an optimal laser-scanning speed), will they recover the M+ state?

6. In Fig. 4c, the authors observed that at N=3, both RCVB and LCVB show identical switching. However, in Fig. S6, the switching appears asymmetric between RCVB and LCVB, especially at a faster scanning speed. The authors should add a discussion about this in the revised manuscript.

7. The authors required multiple pulses to observe AO-HIS; the scanning speed has to be lower for an identical switching scenario for RCVB and LCVB. How does this affect the application of this idea in high-speed, energy-efficient magnetic memory and logic devices?

Based on these observations, I recommend the publication of this manuscript in Nature Communications after revision.

<u>Response to Reviewer 2</u>

Comment | Response

Comment text …………… Reviewer | **Response text …………… Authors**

Reviewer #2 (Remarks to the Authors):

The manuscript presents an interesting experimental study of all-optical switching in Pt/Co multilayers using circularly polarized vector beams under high-NA focusing. The data are extensive and internally consistent. The work addresses an important question in ultrafast magnetism and spintronics, and thus has a potential to be considered for Nature Communications. However, while the results are promising and largely convincing, I have some major conceptual questions which I hope the authors can address.

Response of authors

We would like to thank the reviewer for kindly dedicating the time and effort to reviewing our paper. We greatly appreciate the highly constructive comments from the reviewer *"The data are extensive and internally consistent. The work addresses an important question in ultrafast magnetism and spintronics, and thus has a potential to be considered for Nature Communications."*

In what follows, we address the comments of the reviewer and update the manuscript and supplementary information accordingly. The new updates of the manuscript are marked in blue.

Comments

The manuscript argues that vector beams outperform Gaussian beams due to reduced longitudinal field components and slightly tighter confinement at fixed NA. However, the presented results primarily correlate the transition to helicity-independent switching with reaching wavelength-scale spot sizes. This is not new in fact. As a consequence, it remains unclear whether beam shaping introduces qualitatively new physics or mainly provides an efficient route to this confinement regime. Can the vector field help if the size of the laser spot is larger?

Response of authors

We thank the reviewer for this thoughtful and important comment. At larger spot sizes, corresponding to lower NA, the difference in confinement between Gaussian beams and CPVBs is relatively small (Supplementary Fig. 2), and polarization mixing in Gaussian beams is not very pronounced (Fig. 2E,F). In this regime, Gaussian beams largely preserve the incident polarization at the focal plane and therefore the advantage of vector beams is limited.

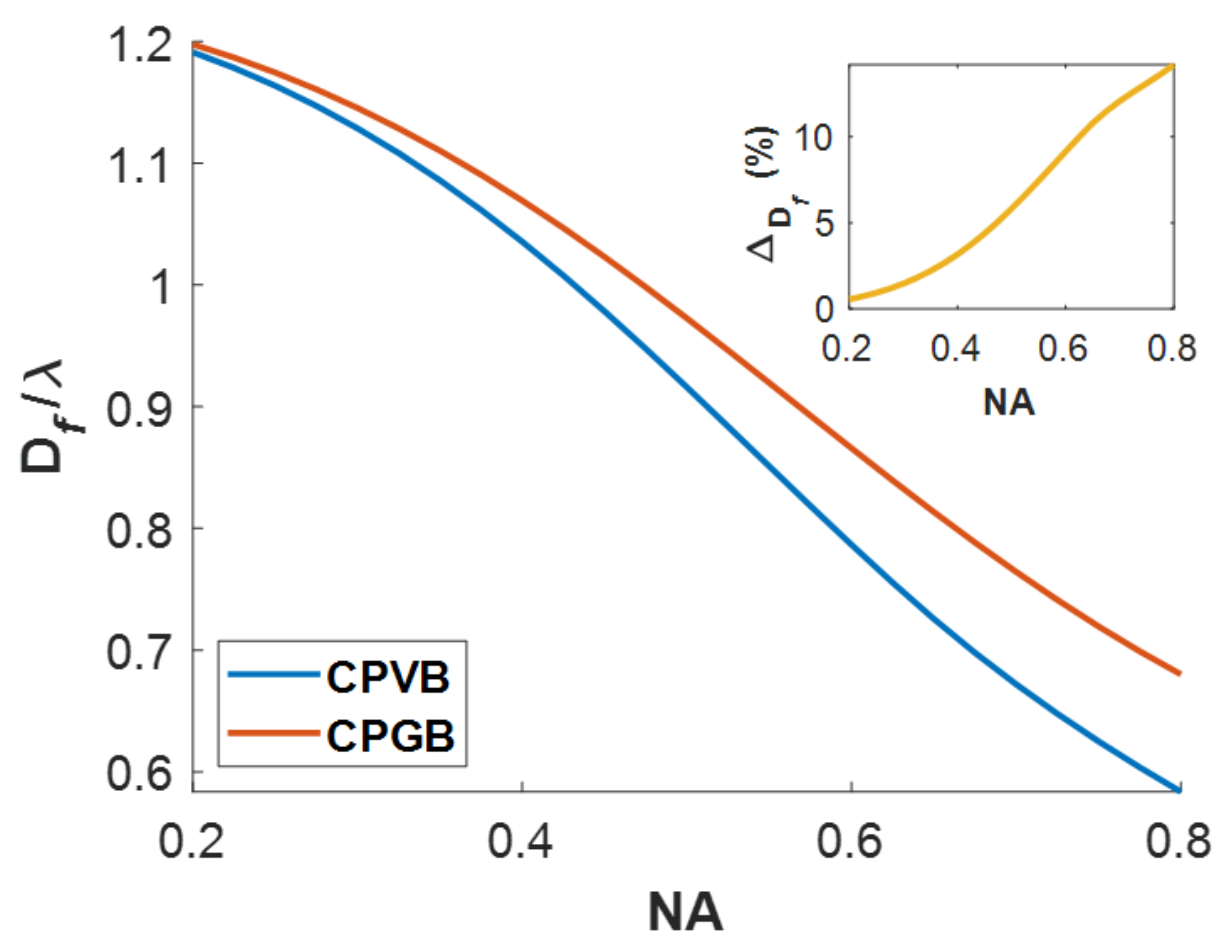

Fig. S2

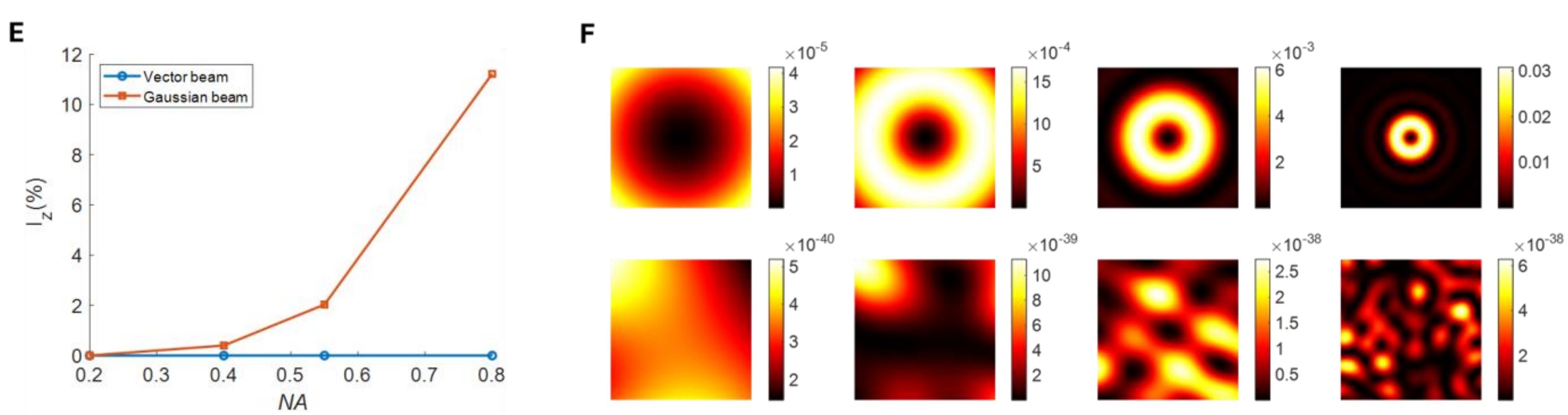

Fig. 2 E, F

In contrast, at smaller spot sizes (i.e., high NA), the difference in confinement becomes more substantial. As seen in Fig 2. E, tight focusing of a Gaussian beam in this regime introduces significant longitudinal field components (up to 12% at N = 0.80) and hence causes polarization mixing, which perturb the incident polarization state at the focal plane. This effect is considerably reduced in the vector beams employed here, which maintain desired transverse polarization while simultaneously achieving stronger confinement. Hence, the advantage of vector beams becomes most significant in the high-NA, tightly confined regime rather than at larger spot sizes.

While the performance difference becomes less pronounced at larger spot sizes, vector beams remain a powerful and versatile tool. They offer a highly effective pathway for achieving enhanced confinement at smaller scales, enabling access to new physical regimes and nanoscale light–matter interactions.

Comments

Suppression of the longitudinal electric-field component is highlighted as a key advantage of vector beams at high NA. It would be useful to clarify whether non-negligible longitudinal fields are expected to directly hinder deterministic switching in Pt/Co multilayers, or whether lateral energy confinement and thermal gradients alone are sufficient to explain the observed behaviour.

Response of authors

We thank the reviewer for this important question and apologize for any confusion caused by our previous wording. We have revised the manuscript to clarify this point.

In the present Pt/Co multilayer system, which exhibits multi-pulse, thermally driven deterministic switching, our results show that lateral energy confinement and the resulting thermal gradients are sufficient to explain the observed switching behavior. Suppressing the longitudinal field component does not introduce a different switching mechanism; rather, it improves control over the polarization at the focal plane to understand the mechanism better.

When a Gaussian beam is tightly focused at high numerical aperture, it generates non-negligible longitudinal field components (up to ~12% at NA = 0.80), leading to polarization mixing at the focal spot. As a result, the local polarization state becomes distorted and harder to define. In contrast, vector beams maintain an almost purely transverse and well-defined polarization at the focus, with negligible longitudinal contribution.

Although these longitudinal fields do not prevent switching in a thermally driven regime, they complicate the interpretation of helicity-dependent versus helicity-independent effects. Vector beams therefore offer two simultaneous advantages: tight lateral confinement, which governs the local energy density and thermal gradients responsible for switching, and suppression of longitudinal components, which ensures that the polarization at the focus faithfully represents the incident state (as shown in Fig. 2E and F). This polarization preservation allows us to disentangle thermal confinement effects from polarization-related field distortions, enabling a clearer assessment of the underlying physics.

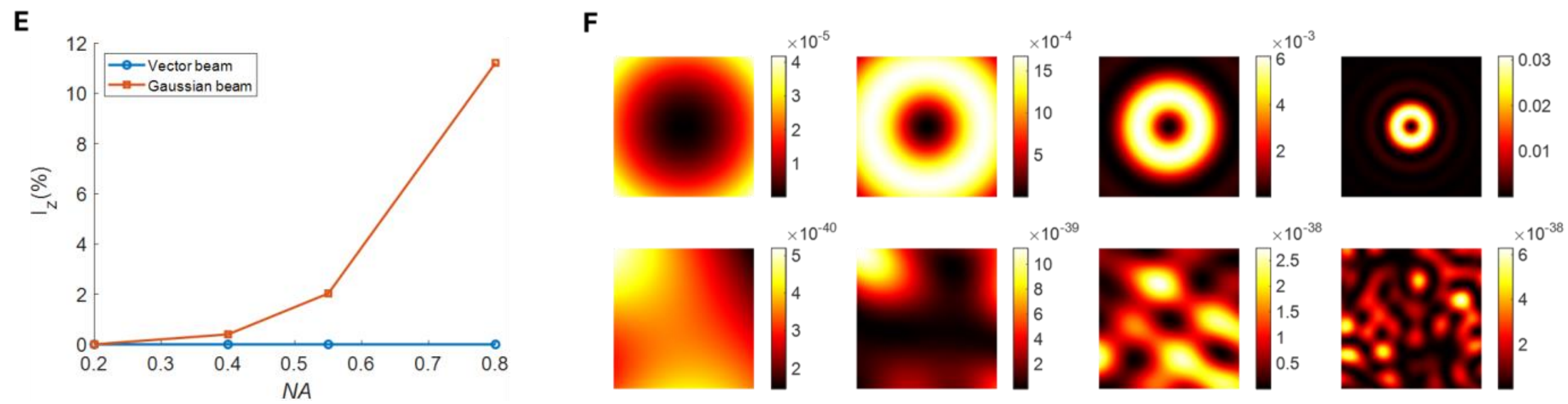

Fig. 2 E, F

While deterministic switching in the Pt/Co multilayers is governed by lateral energy confinement and thermally driven processes, the use of vector beams suppresses longitudinal field components at high NA, ensuring desired polarization at the focal plane at focus and enabling a clearer separation of thermal and polarization-related effects.

Comments

The authors state that vector beams reduce the power required for deterministic switching, but the effect on the intrinsic fluence threshold is not clearly established. Given that the switching is multi-pulse and thermally driven, it would interesting to know whether beam shaping modifies the threshold physics or primarily rescales the local energy density.

Response of authors

We thank the reviewer for this important observation. In the present multi-pulse, thermally driven regime, beam shaping mainly improves spatial confinement and increases local energy density. The reduced switching power therefore results from more efficient energy redistribution, not from a change in the intrinsic fluence threshold.

In accordance with this comment and suggestion, we have removed the statement implying a reduction in the intrinsic switching threshold and revised the manuscript to clarify that vector beam shaping leads to more efficient energy delivery to the switched region. The updated version of the manuscript reflects this distinction more accurately:

A smaller spot size concentrates the light into a tighter area. This makes the light interact more strongly with the material and increases the energy per unit area delivered to the switched region.

We believe this revision improves the physical clarity of the results and aligns the discussion with the underlying thermal switching mechanism.

Comments

The authors refer to nanoscale a lot but I see that switching still occurs on the micron scale. This can be misleading.

Response of authors

We thank the reviewer for raising this important point. We agree that optical imaging alone may give the impression that the switching occurs on the micron scale due to its limited spatial resolution. To address this and avoid any potential ambiguity, we have performed magnetic force microscopy (MFM) measurements, which provide substantially higher spatial resolution and nanoscale magnetic contrast.

An example MFM image is shown here and has now been included in the Supplementary Information for the $[Pt/Co]_N$ (N = 3) sample. For the quantitative analysis, we extracted 10 equally spaced line cuts across the switched region. The corresponding subplot presents the full width at half maximum (FWHM) obtained from each line cut. The measured switched widths range from approximately 0.64 µm to 0.9 µm, yielding an average width of ~0.76 µm with a standard deviation of ~0.09 µm, confirming that the switched region is sub-micron in size.

Based on the reviewer's suggestion, we have added this MFM figure, including the line-cut analysis, to the Supplementary Information and explicitly referenced it in the main text. We believe this addition clarifies the spatial scale of the switching and resolves any potential confusion arising from the limited resolution of optical imaging. We thank the reviewer for this constructive suggestion, which has helped improve the clarity of the manuscript.

Fig.S8 presents representative Faraday microscopy and magnetic force microscopy (MFM) images of the same region of the (N = 3) sample, enabling a direct comparison between the magnetic features observed with both techniques. While the Faraday image qualitatively confirms magnetization reversal, its limited spatial resolution and contrast prevent accurate determination of the switching width. In contrast, the MFM image provides substantially higher spatial resolution and magnetic contrast, enabling precise quantification of the lateral size of the switched region. In the present manuscript, the primary focus is on the development and validation of the optical switching technique and on demonstrating the switching behavior observable with Faraday imaging within the resolution limits of the microscope. Having established the effectiveness of the technique, future investigations can explore magnetic switching of nanoscale magnetic domains using tighter (smaller) optical beams.

To determine the switching width, ten equally spaced line cuts were extracted from the MFM image across the switched area, and the corresponding full width at half maximum (FWHM) values are shown in the accompanying subplot. The measured widths range from ~0.67 µm to ~1.04 µm, yielding an average switched width of ~0.76 µm with a standard deviation of ~0.09 m.

These measurements confirm sub-micron switching in the $[Pt/Co]_N$ (N = 3) sample, resolved beyond the diffraction limit of optical microscopy.

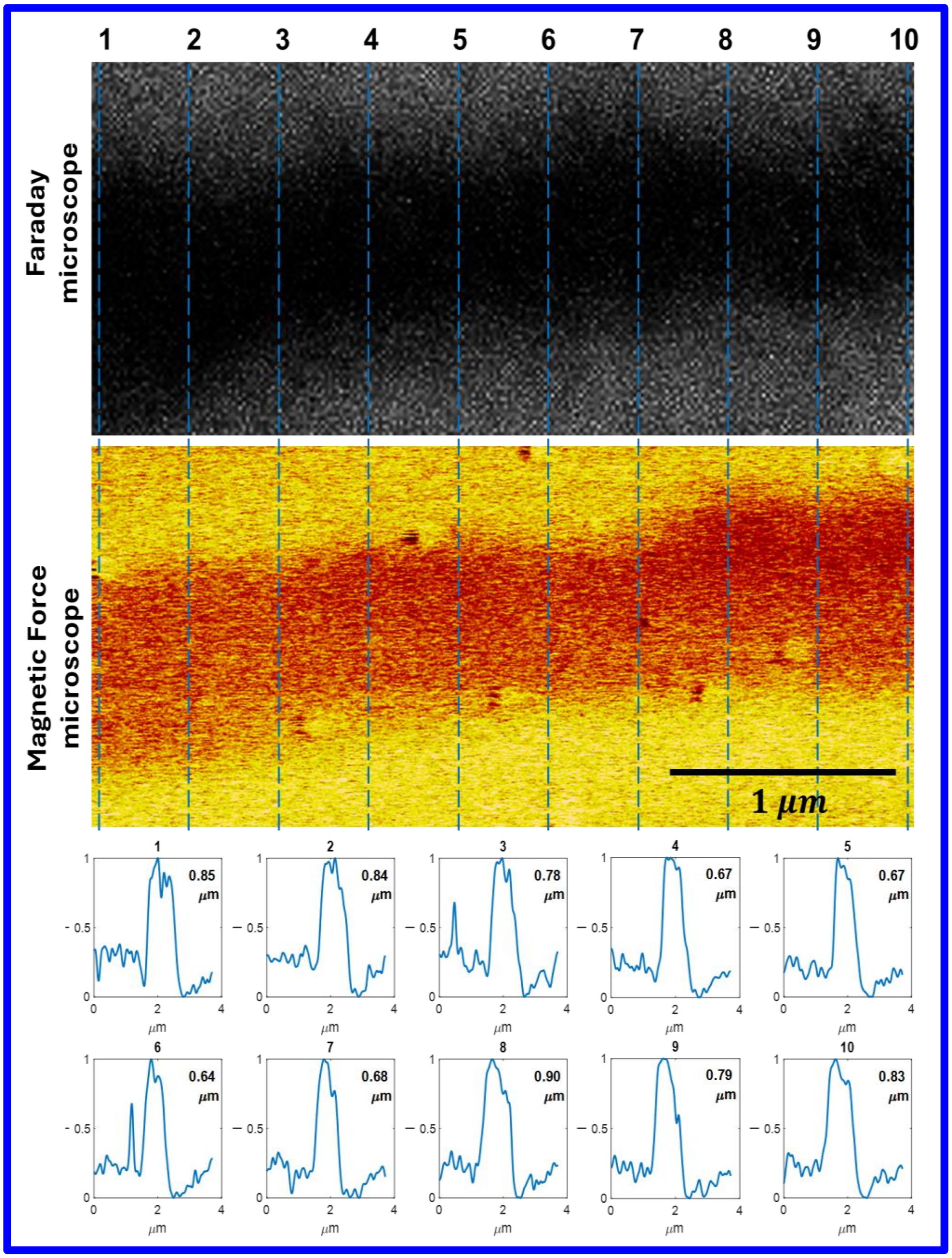

Supplementary Figure S8: *Faraday microscopy and magnetic force microscopy (MFM) images of the switched region in the (N = 3) sample. The MFM image, with higher spatial resolution and magnetic contrast, is used to quantify the lateral switching width. Ten equally spaced line cuts were extracted across the switched area, and the corresponding full width at half maximum (FWHM) values are displayed in the accompanying subplot; the top right corner of each line-cut graph indicates the extracted FWHM.*

Comments

To avoid confusion with single-pulse reports the multi-pulse nature of the presented helicity-independent switching should be emphasized earlier .

Response of authors

We thank the reviewer for this suggestion. This is now corrected in the main manuscript, and we added the following sentence to highlight the multi-pulse nature of helicity-independent switching in our work:

All helicity-independent switching reported in this work occurs in a multi-pulse, thermally driven regime, which is fundamentally different from single-pulse toggle switching mechanisms reported previously.

Comments

The definition of effective spot size should be stated explicitly.

Response of authors

We thank the reviewer for highlighting the need for an explicit definition of the effective spot size. In the updated manuscript, we now explicitly state that the optical beam width is defined according to ISO 11146 as the $1/e^2$ intensity diameter, i.e., the diameter at which the irradiance drops to $1/e^2$ of its peak value. We have added the following line the methods section for clarification:

The optical beam width is defined as the intensity diameter at which the irradiance drops to $1/e^2$ of its peak value.

Comments

I suggest providing spot sizes in Figures 3 and 4.

Response of authors

We thank the reviewer for this helpful suggestion. Following reviewer comments, we have now explicitly provided the spot sizes in Figures 3 and 4, replacing the previously used normalized

notation (e.g., *1.2 λ*, etc.) with the actual beam sizes. This change has been made in the text as well to improve clarity and to allow for a more direct and unambiguous interpretation of the experimental conditions.

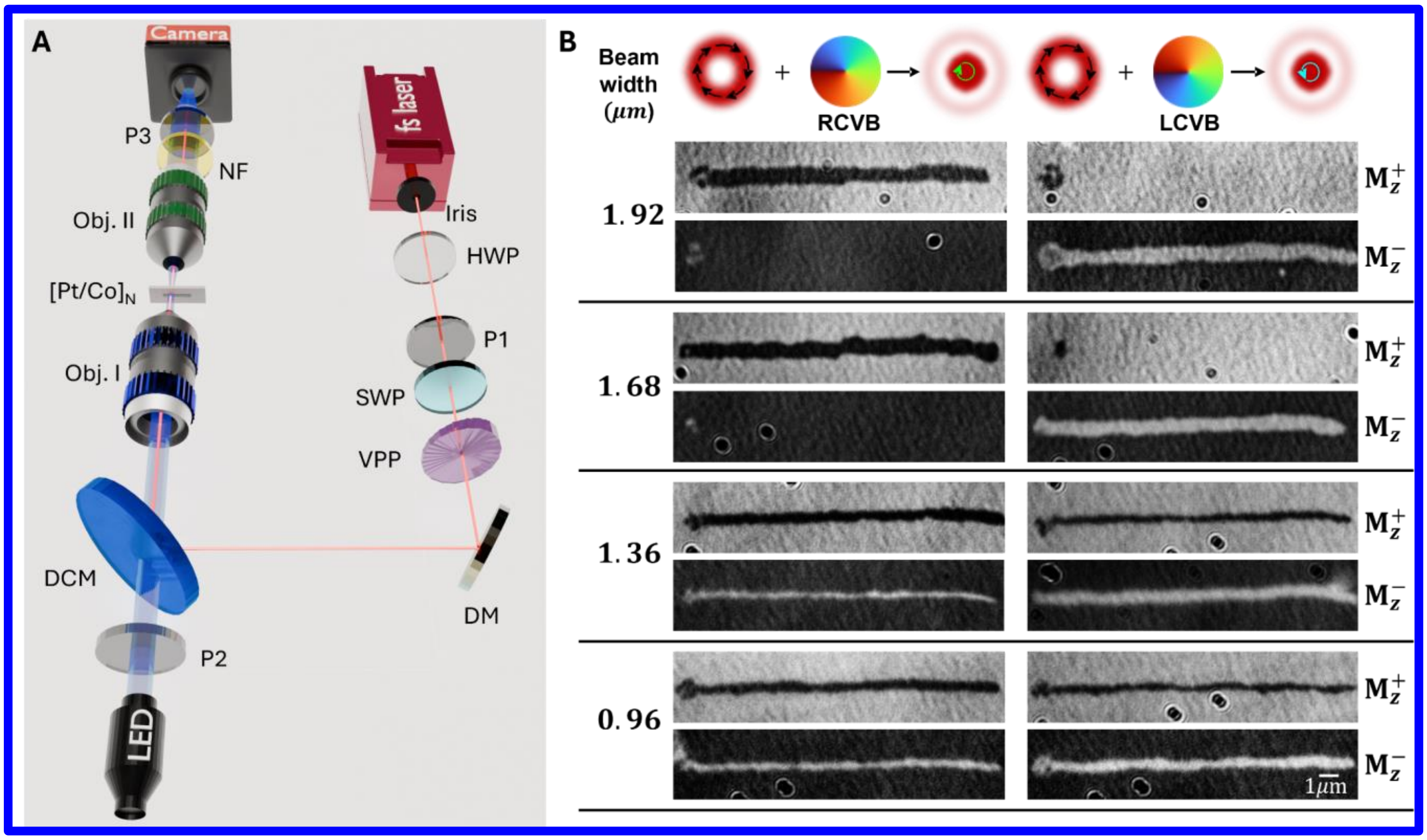

Concluding remarks of authors

We would like to thank the reviewer again for the exceptional quality of their questions. We hope our responses have fully addressed all expressed concerns. The referee's comments have helped us significantly to improve the quality of the paper and its presentation. We are happy to address any further questions and implement any modifications the reviewers suggest.

Response to Reviewer 3

Comment **Response**

Comment text …………… Reviewer **Response text …………… Authors**

Reviewer #3 (Remarks to the Authors):

In the manuscript titled *"*Beam shaping induced reconfigurable magnetic domains," authored by Muhammad Waleed Khalid et al., the authors presented their work on deterministic control of all-optical switching through optical engineering. They have obtained both helicity-dependent and helicity-independent magnetization switching $[Co/Pt]_N$ multilayer using polarization manipulation of the optical pulse, without material modification or external bias. The work is novel in the present context of spintronics research, and I recommend publication of this manuscript in Nature Communication after the following modifications.

Response of authors

We would like to thank the reviewer for kindly dedicating the time and effort to reviewing our paper. We greatly appreciate the highly positive and constructive comments from the reviewer *"The work is novel in the present context of spintronics research, and I recommend publication of this manuscript in Nature Communication after the following modifications."*

In what follows, we address the comments of the reviewer and update the manuscript and supplementary information accordingly. The new updates of the manuscript are marked in blue.

Comments

The authors discussed deterministic switching with respect to the domain size in $[Co/Pt]_N$ multilayers. What is the typical domain size of the $[Co/Pt]_N$ multilayer system under investigation, and how does the domain size change with N?

Response of authors

We thank the reviewer for this insightful question. In response, we have added a detailed discussion on the typical magnetic domain size of the investigated $[Pt/Co]_N$ multilayer system and its dependence on (N). We note that the domain size decreases with increasing N). This discussion has now been included in the Supplementary Information Section 5, where we describe the experimentally observed domain sizes and their evolution with increasing multilayer repetitions. Here is the updated text added in the supplementary:

The domain size decreases systematically with increasing N, due to the increase in effective anisotropy and the resulting modification of the balance between domain wall and dipolar energies. To estimate the magnetic domain size of the investigated [Co/Pt]⍰ multilayers, we employed the analytical model of Kaplan for thin films with strong perpendicular uniaxial anisotropy [1]. Within this framework, assuming negligible domain wall width compared to the stripe period and perpendicular magnetization within the domains, the equilibrium domain size *D* that minimizes the total energy is given by:

$$D = t \times exp[(\pi D_o/2t) + ln(\pi) - 1 + (1/2 - ln(2))]$$

where

$$D_o = \sigma/E_d$$

$$\mu = 1 + (E_d/K_u)$$

where, *t* is the total magnetic thickness, $E_d = 2\pi M_s^2$ is the demagnetizing energy density, $\sigma = 4\sqrt{(A_{ex} K_u)}$ is the domain wall energy per unit area, A_{ex} is the exchange stiffness, and K_u (equivalent here to K_{eff}) is the effective perpendicular anisotropy constant. We only take the thickness of the Co layer since it's the magnetic material in the system.

The saturation magnetization M_s was measured directly by a vibrating sample magnetometer (VSM). The effective anisotropy K_u and exchange stiffness A_{ex} were estimated using literature values reported for comparable Co/Pt multilayer systems [2].

Using these parameters, we obtain equilibrium domain sizes of approximately:

N	t_{co} (nm)	A_{ex} (erg/cm^3) x 10^{-6}	M_s(emu/cm^3)	K_u(erg/cm^3) x 10^7	D (μm)
1	0.6	1.2	1406	0.1392	2688
3	1.8	1.2	1566	0.9728	182.6
5	3.0	1.2	1620	1.3468	7.5
7	4.2	1.2	1592	1.3253	1.6
9	5.4	1.2	1608	1.6124	0.9

Table 1: *Table 1 Calculated equilibrium magnetic domain sizes for [Co/Pt]N multilayers using the Kaplan analytical model with experimentally measured and literature-based parameters.*

1. Kaplan, B., & Gehring, G. A. (1993). The domain structure in ultrathin magnetic films. *Journal of Magnetism and Magnetic Materials*, *128*(1-2), 111-116.

2. Eyrich, Charles, et al. "Exchange stiffness in thin film Co alloys." *Journal of Applied Physics* 111.7 (2012).

Comments

The authors discussed the effect of E_z in Gaussian circularly polarized beams and circularly polarized vector beams in detail. I would like to see the temperature profiles (laser-induced heating) of the samples under investigation for both Gaussian and Vector beam excitation.

Response of authors

We sincerely thank the reviewer for this valuable and constructive suggestion. In response, we will include the COMSOL simulation setup, the electric field profile inside the Pt/Co multilayers for N = 3, a comparison of the electric field and lateral temperature profiles for vector and Gaussian beams, and the temperature comparison profiles for both beams across different N values. These additions will provide a clear and direct visualization of the laser-induced heating and enable a comprehensive comparison of the thermal response under both excitation conditions.

COMSOL simulation setup

To analyze the temperature distribution inside the slab, we performed a time-domain 2D coupled electromagnetic (EM) and heat transfer (HT) simulation using COMSOL Multiphysics® [1]. The center wavelength was set to be at 800 nm.

For the EM simulation, scattering boundary conditions were applied on all outer boundaries of the slab. At the top boundary, a Gaussian beam pulse was introduced as the incident electric field. The amplitude of the electric field was scaled to achieve the desired pulse energy. The Gaussian beam and the Vector beam pulse had a Gaussian-shaped temporal width of 100 fs. The spatial widths of Vector beams and Gaussian beams were 0.96 µm and 1.13 µm respectively, matching the characteristics of the experimental laser beam.

In the heat transfer model, all boundaries were set to surface-to-ambient radiation with an emissivity of 0.9. The thermophysical properties of Platinum (Pt), Tantalum (Ta), and Cobalt (Co) were obtained from [2], while their optical properties were taken from [3].

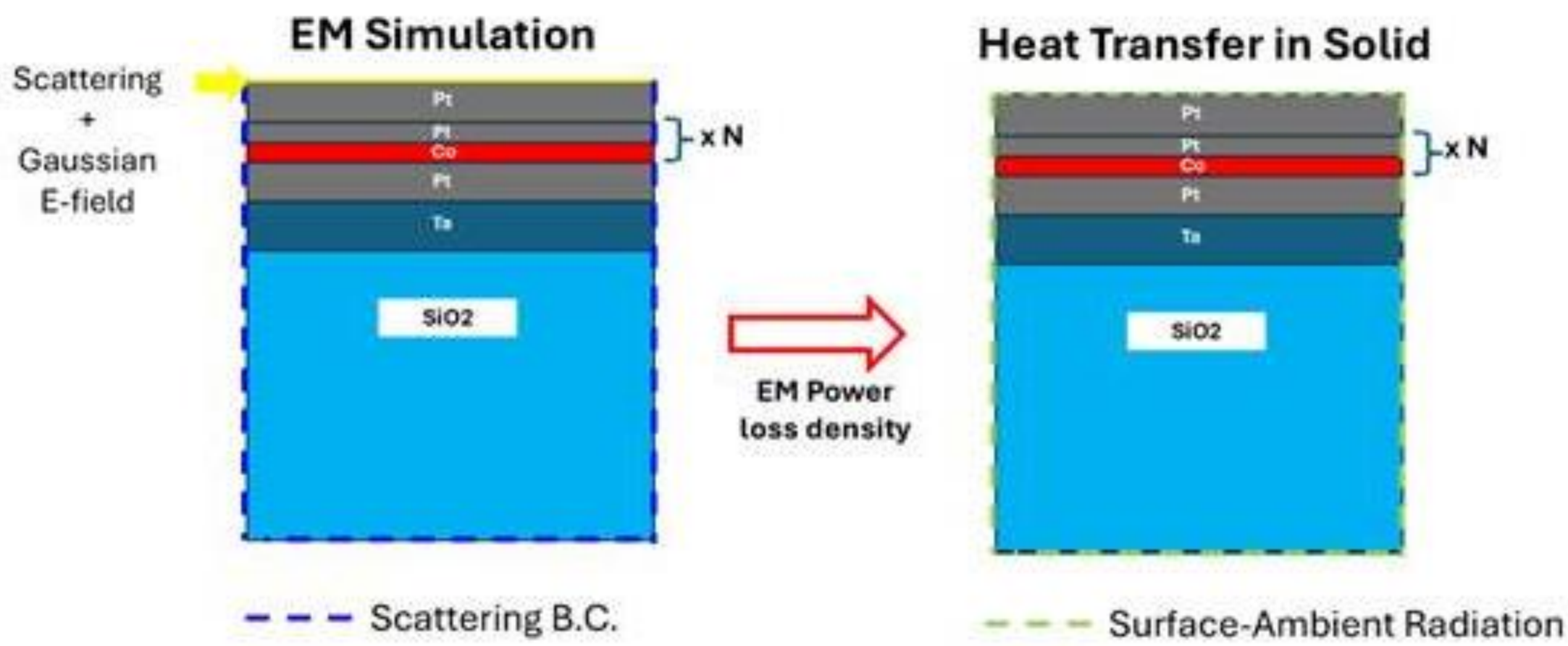

Figure 1: *The electromagnetic dissipated power density was obtained from the EM simulation, which was then used as the heat source in the HT simulation*

Electric Field Profile in Pt/Co multilayers for N = 3

Figure 3 shows the magnitude of the electric field at the peak power for N = 3. The spatial distribution of the E-field inside the structure is shown at the time corresponding to the maximum pulse intensity. The field exhibits diffraction as it propagates away from the source. As shown in the inset on the right, within the Pt/Co multilayer stack, the electric field amplitude appears nearly constant across the layer thickness. This is because the individual layer thicknesses are on the order of a few nanometers, whereas the beam depth of focus is on the micron scale. As a result, the field does not vary significantly over the multilayer thickness, leading to an approximately uniform E-field amplitude inside the multilayer region.

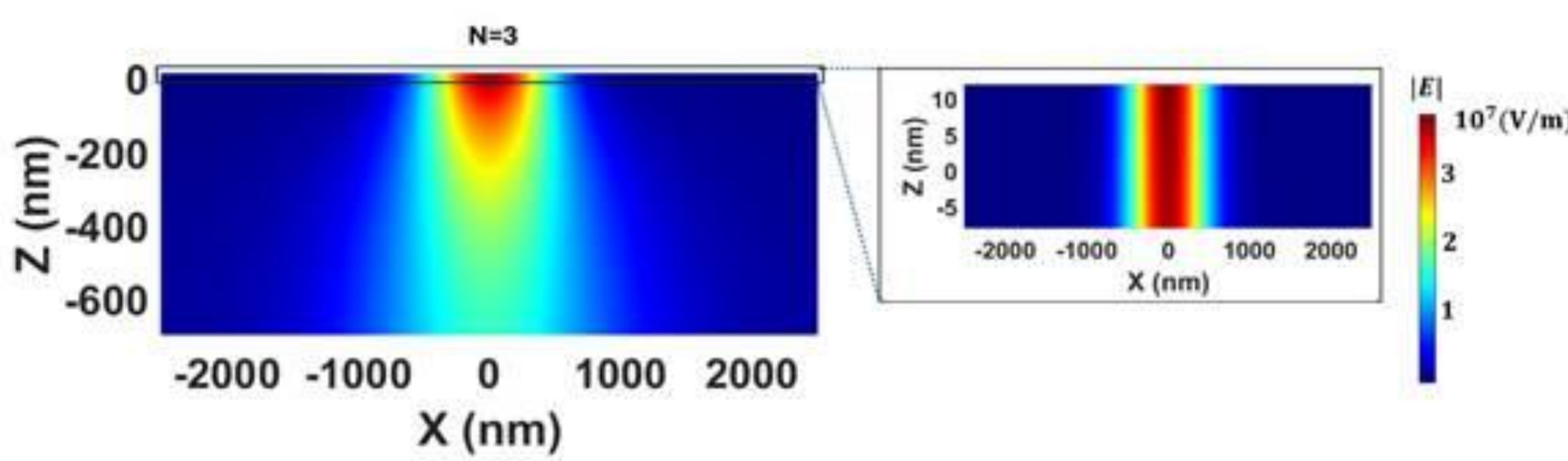

Figure 2: *Magnitude of the electric field at peak power for N = 3, showing diffraction during propagation and an approximately uniform field amplitude within the Pt/Co multilayer due to its nanometer-scale thickness compared to the micron-scale depth of focus.*

Electric Field and lateral temperature profile comparison for Vector beams vs Gaussian beams

Figure 4 shows the comparison of the magnitude of the electric field and the resulting temperature distribution inside the Pt/Co multilayer immediately after the pulse for N = 3. The results are shown for both the vector beam (VB) and the Gaussian beam (GB). While the electric field distributions reflect the different spatial confinement of the two beams, the corresponding temperature profiles illustrate how this difference in energy localization translates into heating within the multilayer stack.

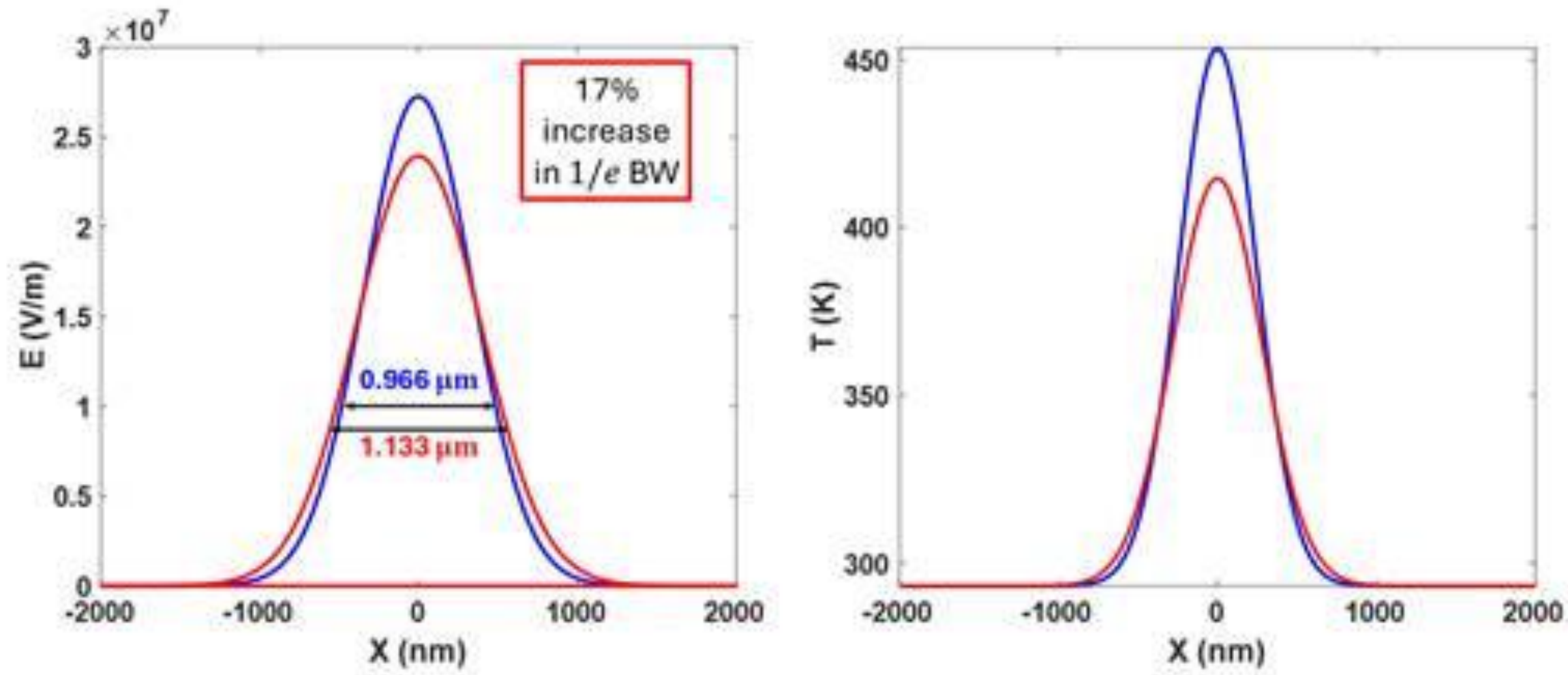

Figure 3*: Comparison of electric field magnitude and resulting temperature distribution in the Pt/Co multilayer for N = 3 under vector beam (VB) and Gaussian beam (GB) excitation.*

Temperature Comparison Profile for Vector beams vs Gaussian beams

The figure below shows the temperature comparison profile for vector beams (left column) and Gaussian beams (right column). The results are shown for multilayer numbers ranging from N = 1 to N = 9. A common color scale (290–460 K) is used across all panels to enable direct comparison of the temperature distribution and its evolution with increasing N. This consistent scale highlights the relative changes in peak temperature and spatial heating profile between the vector and Gaussian beam cases.

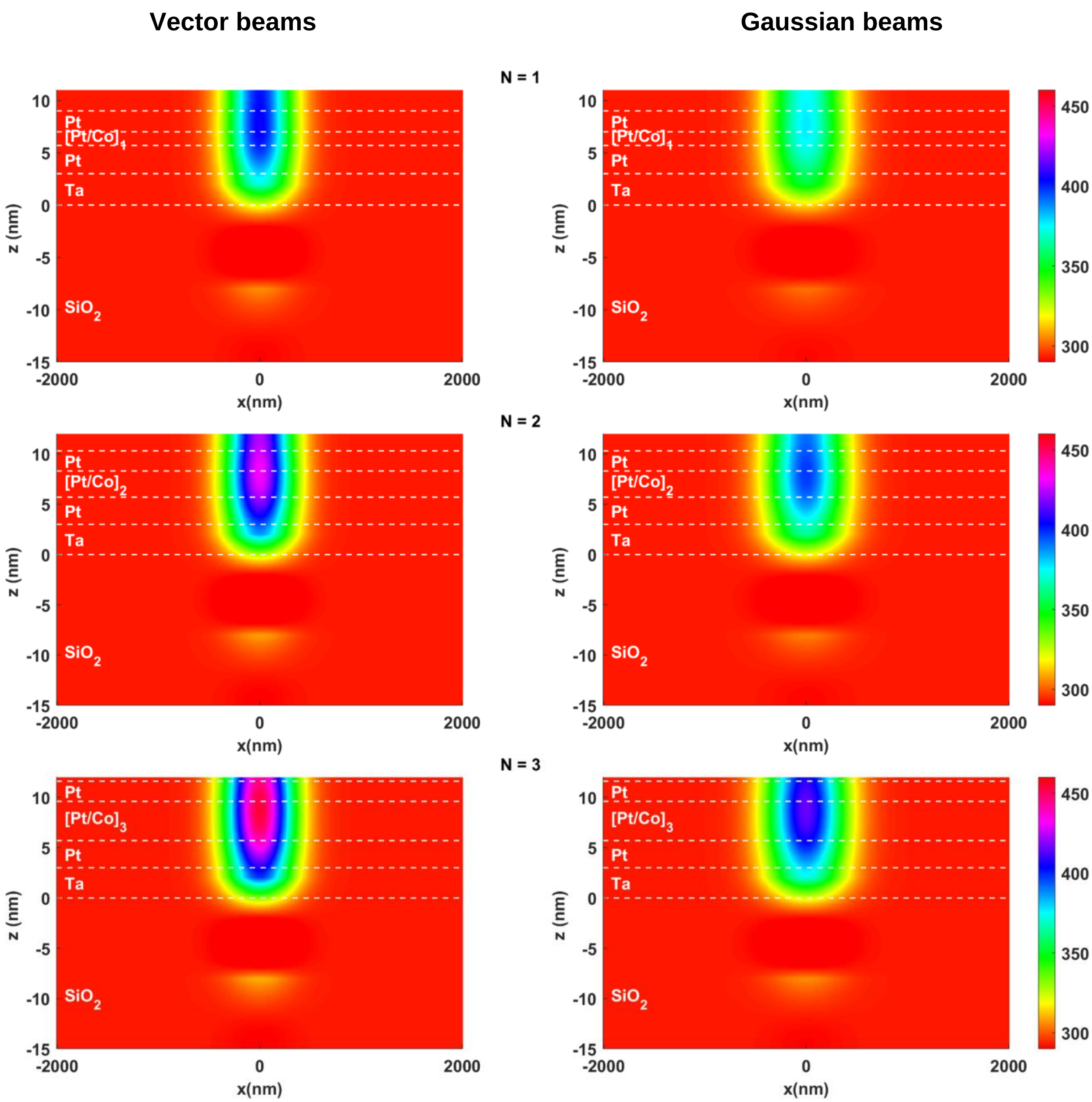

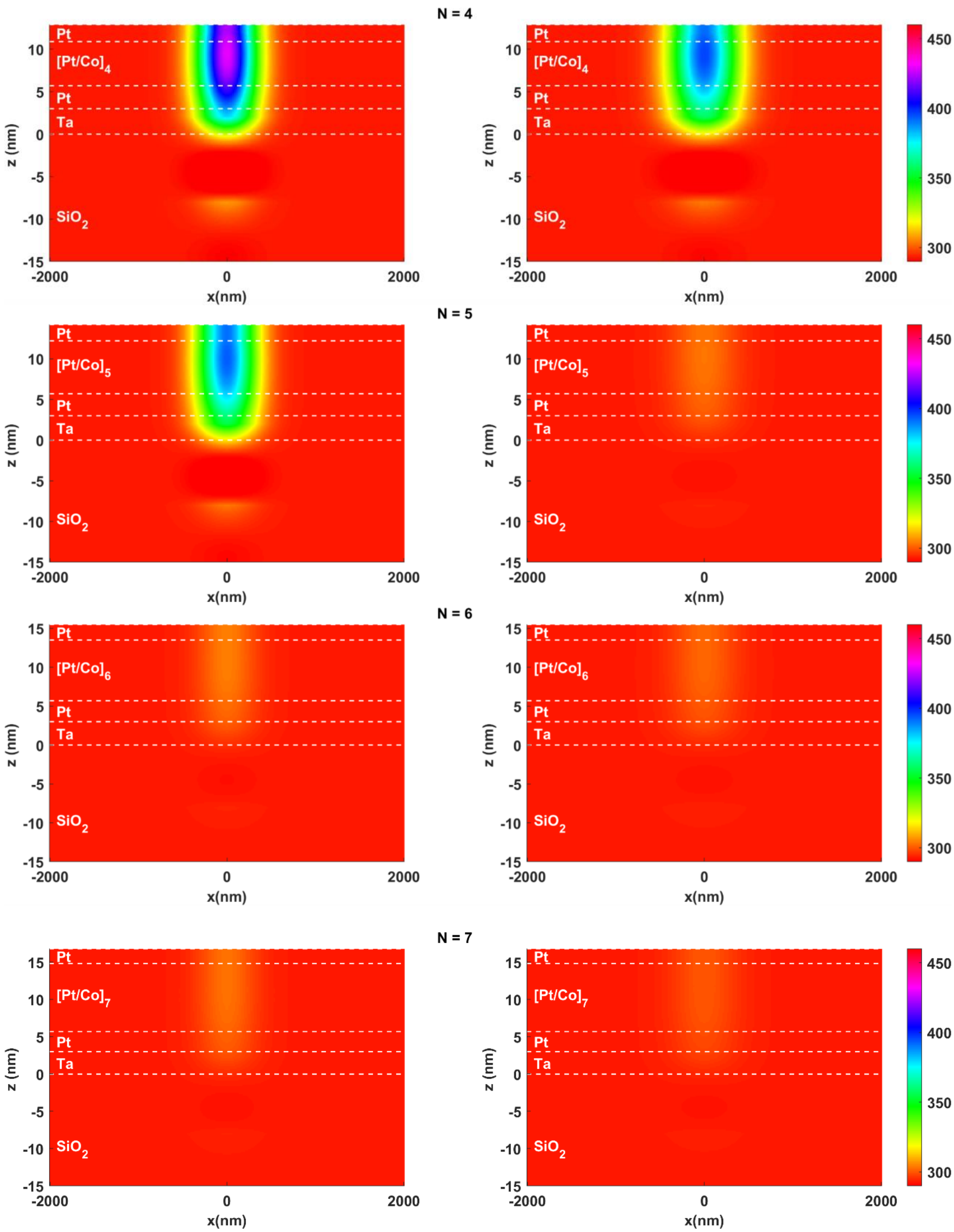
N = 4
N = 5
N = 6
N = 7
Pt
[Pt/Co]4
[Pt/Co]5
[Pt/Co]6
[Pt/Co]7
Ta
SiO2
z (nm)
x(nm)
-2000
0
2000
-15
-10
-5
0
5
10
15
300
350
400
450

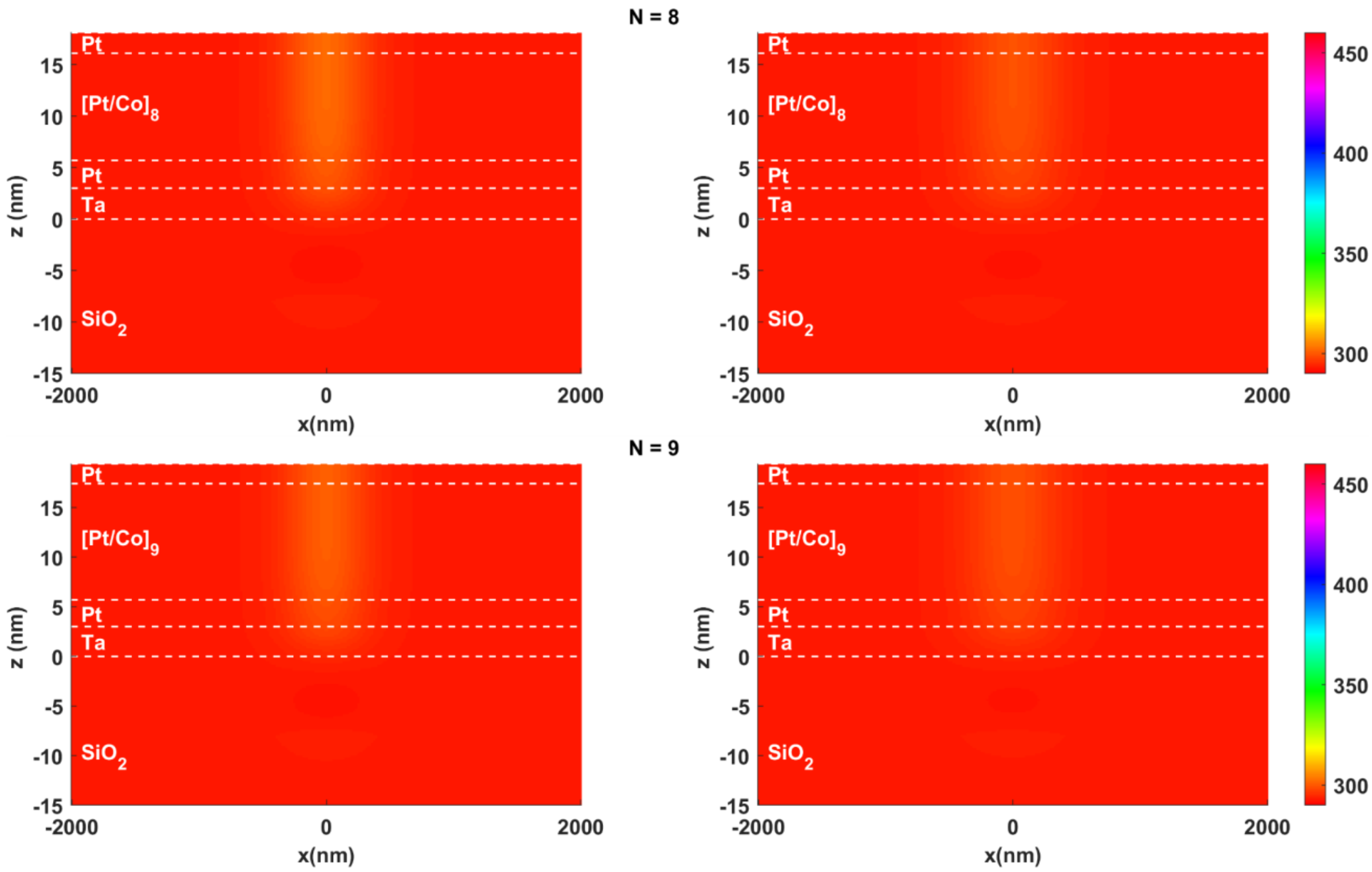

Figure 4: *Temperature distribution comparison for vector beams (left) and Gaussian beams (right) from N = 1 to N = 9, shown with a common 290–460 K color scale to directly highlight differences in peak heating and spatial profiles.*

Average temperature in Pt/Co multilayer for Vector and Gaussian beams across different N

The figure below shows the average temperature within the Pt/Co multilayer stack as a function of N. The values are calculated by averaging the temperature across the Pt/Co region.

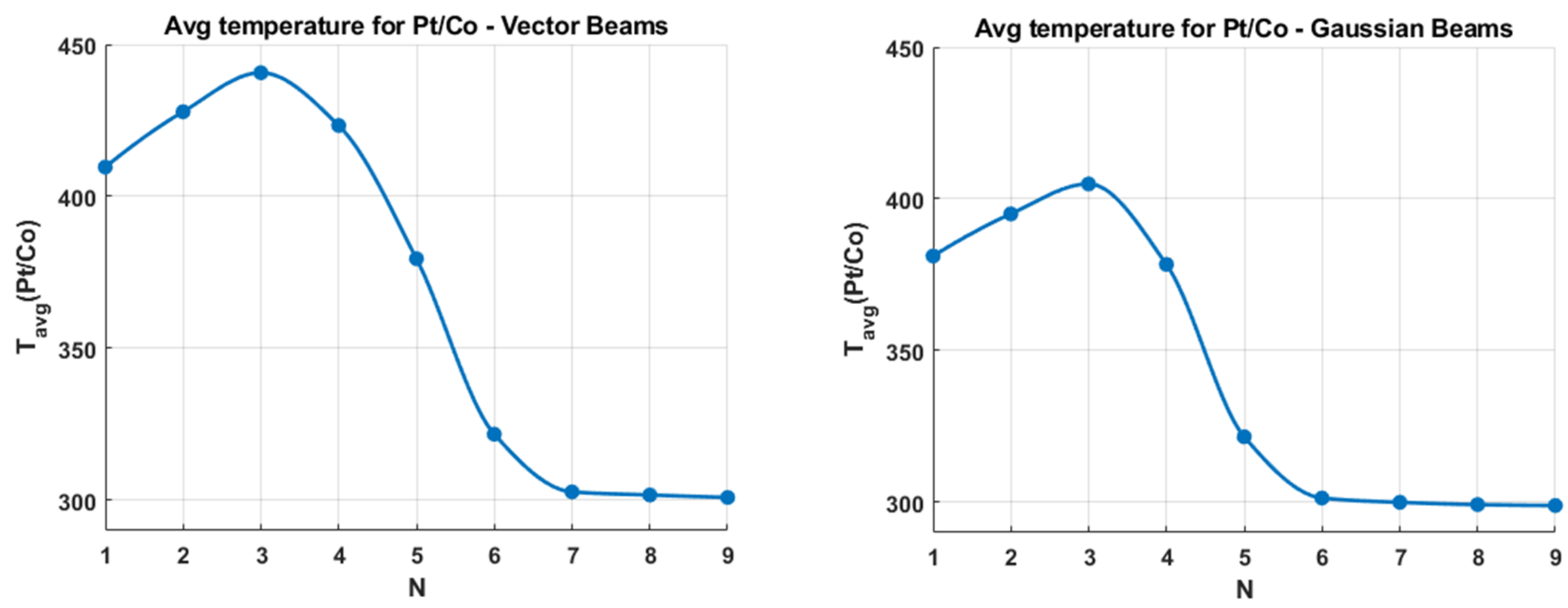

Figure 5: *Average temperature within the Pt/Co multilayer stack as a function of N for Vector Beams (left) and Gaussian Beams (right).*

References:

1. COMSOL Multiphysics® v. 6.2. *www.comsol.com*. COMSOL AB, Stockholm, Sweden.
2. TPSX Materials Database, https://tpsx.arc.nasa.gov/MaterialsDatabase (accessed 2 Feb 3026).
3. Werner, W. S. M., Glantschnig, K. & Ambrosch-Draxl, C. Optical constants and inelastic electron-scattering data for 17 elemental metals. *J. Phys. Chem*. Ref. Data 38, 1013–1092 (2009).

Comments

AO-HIS in these samples still requires a large number of pulses. Roughly, how many pulses are required to observe AO-HIS? Apart from mentioning the beam widths, the authors should also report the pulse energy and compare it with the switching energy for single-shot AO-HIS observed in typical systems. Can we deliver such pulse energy using a fibre laser compatible with on-chip photonics?

Response of authors

We thank the reviewer for this important comment. In response, we have now included the pulse energy, the estimated total deposited energy, and a comparison with representative single-shot AO-HIS switching energies reported in the literature. This information has been added to the Supplementary Information, where we also discuss the approximate number of pulses required under our experimental conditions and place our results in the context of previously reported systems.

At a scanning speed of 5 µm s^{-1}, a laser repetition rate of 1 kHz and spot size of 1 µm, approximately *200* laser pulses are delivered within a 1 $µm^2$ area, corresponding to the effective laser spot size.

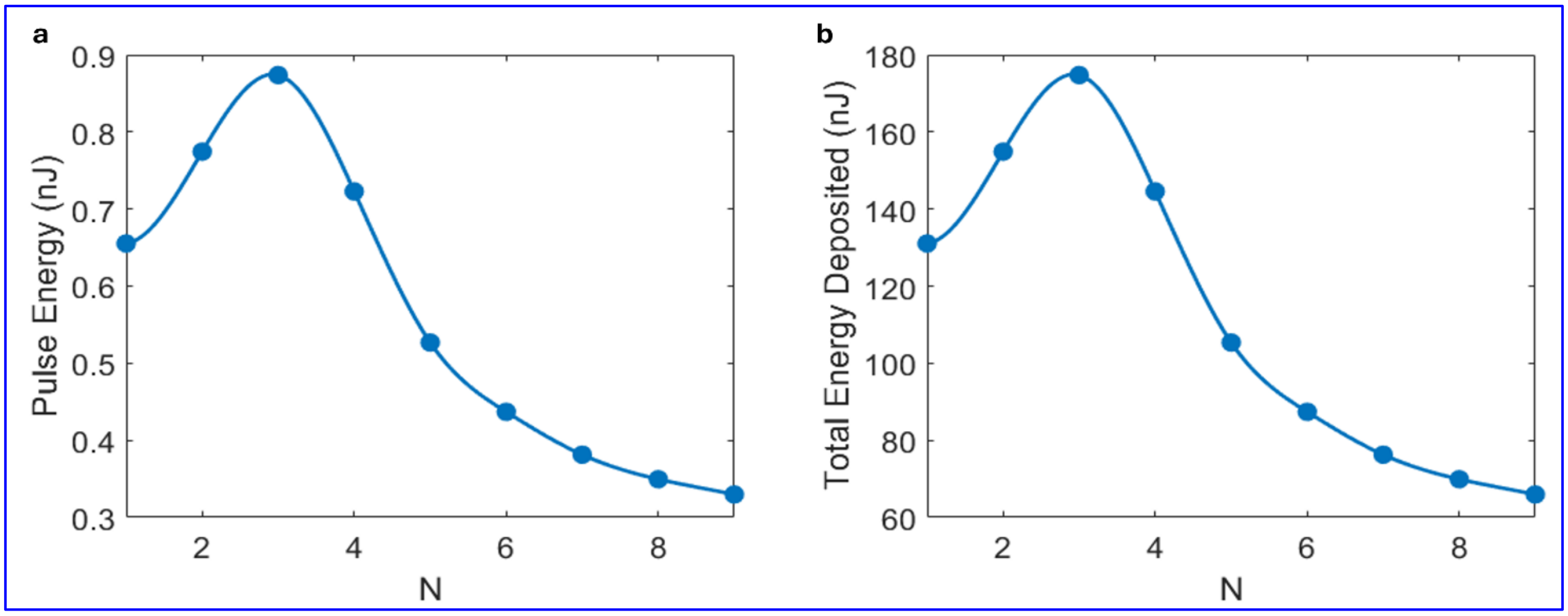

Figure S12: *(a) Single-pulse (b) Total energy required to achieve all-optical switching (AOS) for multilayers with N = 1–9).*

**Material**	**Pulse Energy (nJ)**	**Pulses Needed**	**Total Energy (nJ)**	**Link**
GdFeCo	540	1	540	https://journals.aps.org/prb/abstract/10.1103/PhysRevB.94.064412
Co/Pt/GdFeCo	∼ 1000	1	1000	https://pubs.aip.org/aip/apl/article/111/4/042401/34968/Single-shot-ultrafast-all-optical-magnetization
Pt/Co/Gd	180	1	180	https://journals.aps.org/prb/abstract/10.1103/PhysRevB.96.220411
Tb/Co	9.1	1	9.1	https://doi.org/10.1103/PhysRevResearch.5.023163
**Our Work**	**< 0.9**	**200**	**65 - 175**	

Table 2: *Summary of materials, corresponding pulse energy, number of pulses required, and total accumulated energy for switching; the reported multi-pulse total energy remains lower than several comparable single-shot helicity-independent AOS demonstrati*

Laser sources which can do this

Yes, we can deliver ∼nJ-level pulse energies using a femtosecond fiber laser in a way that's compatible with on-chip photonics.

As an example, below two femtosecond fiber-laser sources that can be used for this purpose:

- ELMO-780-HIGH-POWER by Thorlabs
- C-Fiber 780 by Menlo Systems

Comments

Why don't the $[Co/Pt]_N$ sample show single-shot toggle-type AO-HIS? (like in GdFeCo alloys and exchange-coupled $[Co/Pt]_N$ multilayers?). Authors should include a discussion in the revised manuscript.

Response of authors

We thank the reviewer for this important and insightful question. The absence of single-shot toggle-type AO-HIS in the present $[Co/Pt]_N$ multilayers can be understood from a combination of intrinsic material properties, stack architecture, and lateral domain stabilization, all of which place the system in a fundamentally different switching regime from ferrimagnetic alloys and exchange-coupled hybrid structures.

The investigated stack, $Pt(2.7)/[Pt(0.7)/Co(0.6)]_N/Pt(2.0)/Ta(3.0)$, is a purely ferromagnetic multilayer. Unlike ferrimagnetic systems such as GdFeCo, which exhibit single-shot toggle-type AO-HIS driven by ultrafast angular-momentum exchange between antiferromagnetically coupled sublattices [1–3], the present Co/Pt multilayers consist of a single ferromagnetic sublattice and therefore lack the compensation physics and transient ferromagnetic-like states that underpin deterministic single-pulse reversal. In such ferromagnetic systems, single-pulse excitation predominantly induces ultrafast demagnetization followed by stochastic domain nucleation and recovery, rather than deterministic toggle switching [4].

Reports of single-shot switching in Co/Pt-based systems generally rely on exchange coupling to an adjacent ferrimagnetic layer (for example, Co/Pt multilayers grown on or coupled to GdFeCo) [5]. In these hybrid structures, the ferrimagnetic layer undergoes single-shot toggle AO-HIS and subsequently transfers a deterministic reversal tendency to the ferromagnetic Co/Pt multilayer through interlayer exchange coupling. This exchange-mediated pathway effectively introduces an internal bias and additional angular-momentum transfer channels that are absent in standalone Co/Pt multilayers. In the present samples, no ferrimagnetic underlayer or exchange-coupled compensation physics is present; the Pt seed and capping layers primarily enhance perpendicular magnetic anisotropy and spin–orbit effects, while the Ta layer acts as a structural and thermal sink.

In addition to these intrinsic considerations, lateral domain stabilization plays a critical role in suppressing toggle-type behavior in our samples. The laser-excited region remains embedded within a surrounding ferromagnetic domain configuration that energetically favors the pre-

existing magnetization orientation. As a result, even when transient demagnetization occurs following single-pulse excitation, the magnetization preferentially relaxes back toward the locally stabilized state imposed by neighboring domains, rather than undergoing deterministic toggle reversal. This domain-holding effect further inhibits single-shot switching and favors cumulative, multi-pulse behavior.

Taken together, the absence of ferrimagnetic sublattice dynamics, the lack of exchange coupling to a ferrimagnet, and strong lateral stabilization by neighboring domains naturally place the present Co/Pt multilayers in a multi-pulse, thermally driven switching regime, consistent with prior studies of standalone ferromagnetic Co/Pt multilayers. We have added the following discussion in the revised manuscript to explicitly clarify this distinction and to position our results relative to single-shot toggle AO-HIS observed in ferrimagnetic and exchange-coupled systems:

Single-shot toggle-type AO-HIS is not expected in the present Co/Pt multilayers, which are purely ferromagnetic and lack the ferrimagnetic sublattice dynamics responsible for deterministic single-pulse switching observed in ferrimagnets, such as GdFeCo[1,2,3]. In standalone Co/Pt multilayers, single-pulse excitation leads to demagnetization and domain nucleation, with deterministic reversal emerging only through multi-pulse thermal accumulation or exchange coupling to a ferrimagnet, while lateral stabilization by surrounding domains further suppresses toggle-type reversal [4,5,6,7]**.** In our experiments, multi-pulse measurements show no deterministic single pulse reversal, but instead, switching proceeds cumulatively via nucleation and growth under repeated pulses. The dipolar field dominates the switching criterion when the wavelength-scale focusing condition is met.

References

1. Mangin, S. *et al.* Engineered materials for all-optical helicity-dependent magnetic switching. *Nat. Mater.* **13**, 286–292 (2014).
2. Ostler, T. A. *et al.* Ultrafast heating as a sufficient stimulus for magnetization reversal in a ferrimagnet. *Nat. Commun.* **3**, 666 (2012).
3. Gorchon, J. *et al.* Role of electron and phonon temperatures in the helicity-independent all-optical switching of GdFeCo. *Phys. Rev. B* **94**, 184406 (2016).
4. El Hadri, M. S. *et al.* Two types of all-optical magnetization switching mechanisms using femtosecond laser pulses. *Phys. Rev. B* **94**, 064412 (2016).
5. Gorchon, J. *et al.* Single shot ultrafast all optical magnetization switching of ferromagnetic Co/Pt multilayers. *Appl. Phys. Lett.* **111**, 042401 (2017).

6. Medapalli, R. *et al.* Multiscale dynamics of helicity-dependent all-optical magnetization reversal in ferromagnetic Co/Pt multilayers. *Phys. Rev. B* **96**, 224421 (2017).

7. Parlak, U., Adam, R., Bürgler, D. E., Gang, S. & Schneider, C. M. Optically induced magnetization reversal in [Co / Pt] N multilayers: Role of domain wall dynamics. *Phys. Rev. B* **98**, 214443 (2018).

Comments

The authors start from M+ state, use RCVB to show HI-AOS. Then, if they use LCVB in the same switched region (at an optimal laser-scanning speed), will they recover the M+ state?

Response of authors

We thank the reviewer for this important question. We note that the switching behavior depends on the multilayer repeat number and corresponding spot size. For N=1 and N=2, since the dominant mechanism is helicity-dependent switching, we can reverse the magnetization back to its original state using the opposite circular vector beam polarization.

For N=3, where the effective spot sizes are larger (∼1.92 μm and ∼1.68 μm) and the dominant mechanism is helicity-dependent switching, we can reverse the magnetization back to its original state using the opposite circular vector beam polarization. For N=3, where the effective spot sizes are smaller (∼1.36 μm and ∼0.96 μm) and the dominant mechanism is helicity-independent switching, we cannot reverse the magnetization back to its original state using the opposite circular vector beam polarization.

Further, for N=4 and higher repetitions, where the switching becomes fully helicity-independent, such optical reversal using opposite vector beam polarization is also no longer observed. In this regime, we explored a broad range of laser energies, from below the switching threshold through the deterministic switching regime and up to the onset of sample damage and did not observe optical reversal back to the original state. Reversal was only observed when an external magnetic field was applied, in which case the magnetization within the laser-excited region aligned with the direction of the applied field. This behavior is consistent with the multi-pulse, thermally driven nature of the helicity-independent all-optical switching (HI-AOS) reported here.

Comments

In Fig. 4c, the authors observed that at N=3, both RCVB and LCVB show identical switching. However, in Fig. S6, the switching appears asymmetric between RCVB and LCVB, especially at a faster scanning speed. The authors should add a discussion about this in the revised manuscript.

Response of authors

We thank the reviewer for pointing out this apparent discrepancy. While Fig. 4c shows identical switching behavior for RCVB and LCVB at N = 3, Fig. S6 exhibits an apparent asymmetry, particularly at higher scanning speeds. We have now added a discussion in the revised manuscript to clarify this behavior.

In our experiments, the maximum scanning speed accessible with the motorized translation stage was ~30 μm s^{-1}, while the laser repetition rate was 1 kHz. Under these conditions, the spatial overlap of successive pulses is governed by the mechanical motion of the stage rather than by the intrinsic ultrafast magnetization dynamics of the Pt/Co multilayers. As a result, at faster scanning speeds (~30 μm s^{-1}) the cumulative heating becomes sensitive to discretization and timing limitations of the stage, which can manifest as apparent asymmetries between RCVB and LCVB in the supplementary measurements.

In addition, N=3 represents a transitional regime in our system, marking the crossover from helicity-dependent to helicity-independent switching. In such a crossover regime, small variations in energy deposition or pulse overlap, like those introduced by stage-limited scanning at higher speeds, can reasonably lead to partial or asymmetric switching responses. The observation of such asymmetry in Fig. S6 is therefore expected and does not contradict the helicity-independent behavior observed under optimized conditions in Fig. 4c.

We emphasize that these effects arise from experimental limitations and the transitional nature of the N=3 system, rather than from a fundamental asymmetry in the switching mechanism. We therefore expect that, with scanning speeds comparable to the intrinsic material dynamics, the behavior for RCVB and LCVB would converge, consistent with helicity-independent switching. We thank the reviewer for this constructive comment, which has helped us clarify both the experimental constraints and the role of the transitional regime in the observed behavior. Based on this observation, we have added the following text in the supplementary:

In figure S6, the apparent asymmetry between RCVB and LCVB at higher scanning speeds for N=3 arises from stage-limited pulse overlap combined with the transitional nature of this multilayer, which marks the crossover from helicity-dependent to helicity-independent switching.

Comments

The authors required multiple pulses to observe AO-HIS; the scanning speed has to be lower for an identical switching scenario for RCVB and LCVB. How does this affect the application of this idea in high-speed, energy-efficient magnetic memory and logic devices?

Response of authors

In summary, we demonstrate a versatile beam-engineering approach that enables deterministic control of all-optical magnetization switching in technologically relevant ferromagnetic multilayer ferromagnetic multilayers. By co-optimizing the multilayer geometry and optical excitation conditions, robust polarization-independent switching is achieved at sub-wavelength length scales, overcoming the helicity constraints, which can limit the device integration. Future studies investigating material-dependent switching thresholds and the dynamic evolution of magnetic domains will provide further insight into the underlying mechanisms and guide the rational design of next-generation opto-magnetic platforms capable of ultrafast, all optical data storage.

We believe this revision improves the focus of the paper and ensures that the discussion remains aligned with the experimental regime demonstrated and its primary objective of elucidating the underlying physics.

Concluding remarks of authors

We would like to thank the reviewer again for the exceptional quality of their questions. We hope our responses have fully addressed all expressed concerns. The referee's comments have helped us significantly to improve the quality of the paper and its presentation. We are happy to address any further questions and implement any modifications the reviewers suggest.

Response to Reviewer #2

Comment   Response

Comment text ……………. Reviewer   **Response text ……………. Authors**

Reviewer #3 (Remarks to the Authors):

I thank the authors for their detailed responses and for the improvements made to the manuscript.

The authors provided an extensive explanation of the differences in switching behavior between tightly focused vector beams and Gaussian beams. However, the central conclusions are currently supported only by simulations which they also include in the manuscript. Since this is an experimental work, I believe the key claim can and should be verified experimentally in a straightforward manner.

Response of authors

We sincerely thank the reviewer for the positive assessment of our detailed responses and for recognizing the improvements made to the manuscript. We also appreciate the reviewer's acknowledgement that our explanation of the differences between tightly focused vector beams and Gaussian beams is extensive and that the proposed comparison provides a clear and direct experimental route to test the central mechanism.

In what follows, we address the comments of the reviewer and update the manuscript and supplementary information accordingly. The new updates of the manuscript are marked in blue.

Comments

In particular, the experiment proposed schematically in Fig. 3a can serve as a direct "smoking-gun" test. The authors should first demonstrate switching using the tightly focused vector beam generated with the SVV. Then, under otherwise identical experimental conditions (same laser power, focusing conditions, and polarization state), they should simply remove the SVV element and compare the switching behavior for a conventional but tighly focused circularly polarized Gaussian beam.

Such a comparison would directly demonstrate whether the vectorial nature of the beam is indeed essential for the observed switching. For example, the Gaussian beam may fail to switch, require substantially different conditions, or produce qualitatively different behavior. Any of these outcomes would help to clarify role the vectrorial fields.

Without this experimental verification, I unfortunately cannot recommend publication at this stage, because simulations alone are not sufficient to substantiate such a central and interesting claim. However, if these measurements are performed and the results clearly support the proposed mechanism, I would have no objection to publication of this interesting work in Nature Communications.

I also encourage the authors to include these measurements either in the main text or in the Supplementary Information.

Response of authors

We thank the reviewer for this constructive suggestion, which helped us strengthen the manuscript by adding a direct experimental control comparing vector-beam and Gaussian-beam switching behavior. We found the reviewer's *"smoking-gun"* experiment suggestion highly constructive, and we agree that performing this control measurement is the most direct way to clarify the role of vectorial fields.

In response, new experimental results have been added to the revised manuscript and Supplementary Information. The updated Supplementary figures now show the quarter waveplate based helicity control used for the Gaussian beam. The revised Supplementary Information also includes the direct Gaussian-beam control measurement performed under the same conditions as the vector-beam experiment.

The Gaussian-beam control experiment was performed by removing the vector-beam-generating optics and generating right- or left-circular polarization using a quarter-wave plate oriented at +45° or -45° respectively, with respect to the polarizer. Below is the modified setup for the performed experiments:

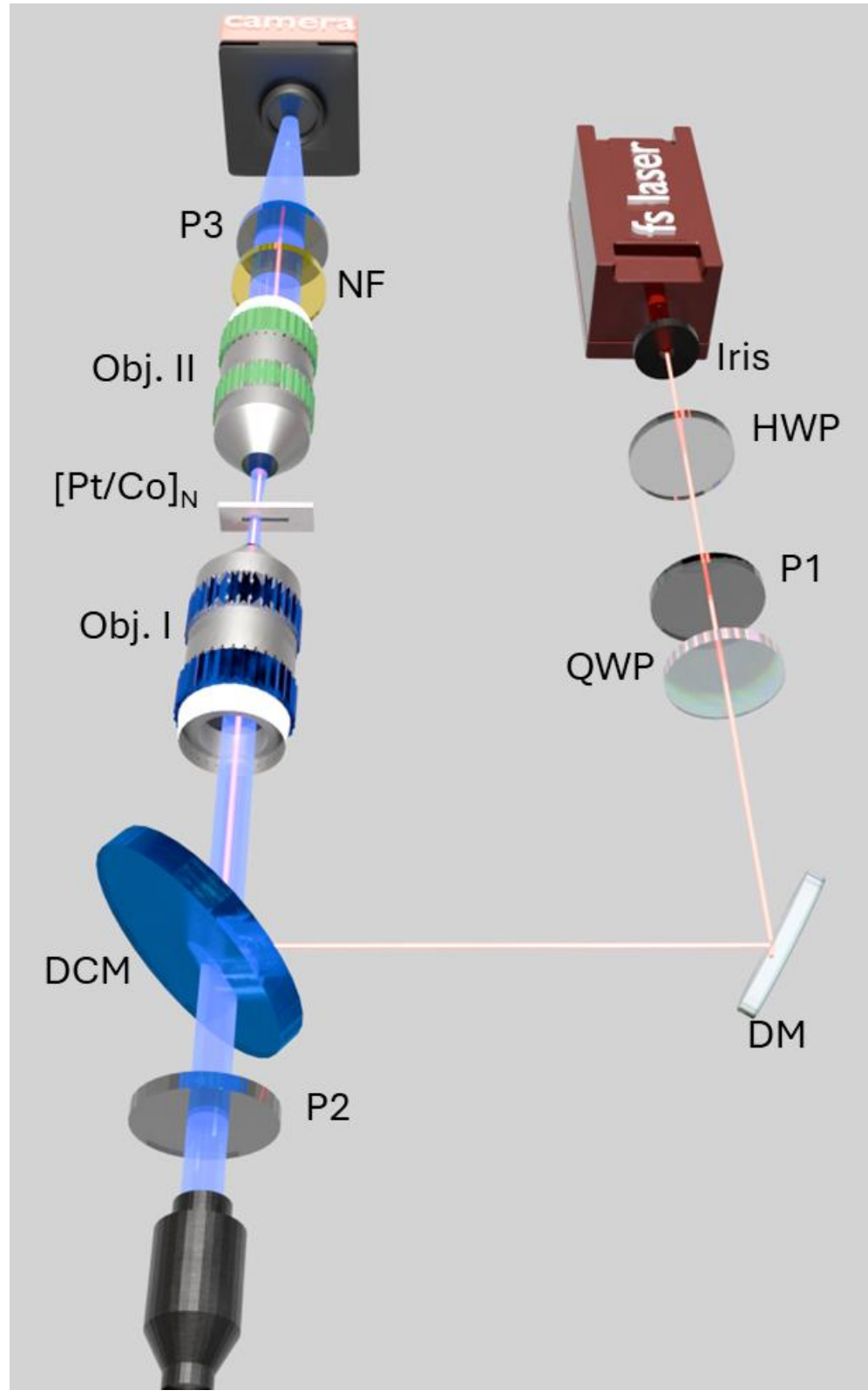

Fig. S13 Optical setup used for circularly polarized Gaussian beams. A linearly polarized femtosecond (fs) laser beam is trimmed using an iris. A half-wave plate (HWP) and polarizer (P1) are used to control the optical power and set the reference polarization. Circularly polarized Gaussian beams are generated using a quarter-wave plate (QWP). The fs pulses are reflected by a dielectric mirror (DM) and combined with LED illumination at a dichroic mirror (DCM). The LED light and fs pulses are focused onto the sample using Obj I, while the sample image is relayed to the camera through Obj II and an 800 nm notch filter (NF), which blocks the fs pulses. A Faraday microscope is formed using polarizer P2 and analyzer P3, providing magnetization contrast.

We performed a direct comparison using a conventional circularly polarized Gaussian beam under experimental conditions as for the vector-beam measurements, including similar focusing, optical power, and polarization state, as shown in Figs. S14 and S15. Under these conditions, the Gaussian beam no switching is observed. For N = 3-5, Gaussian-beam exposure resulted in permanent sample damage. For N = 6-9, it produced non-deterministic magnetic domain formation, characterized by multiple randomly nucleated domains rather than a well-defined switched region as we see with the vector beam. The evolution of these domains with increasing N is consistent with the larger magnetic volume and the lower optical energy required to induce magnetic changes in thicker multilayers.

To distinguish reversible magnetic switching from permanent optical modification, we further performed an external magnetic-field erasure test, which is essential for confirming all-optical

switching in these thin films. After optical exposure, a permanent magnet was brought near the sample to reset the magnetization to its initial state. The regions exposed to the Gaussian beam under comparable conditions did not exhibit the same reversible, well-defined response observed for the vector beam. In particular, for N = 3-5, the optically modified regions could not be erased by the external magnetic field, confirming that the observed contrast arise from permanent sample damage rather than reversible magnetization switching.

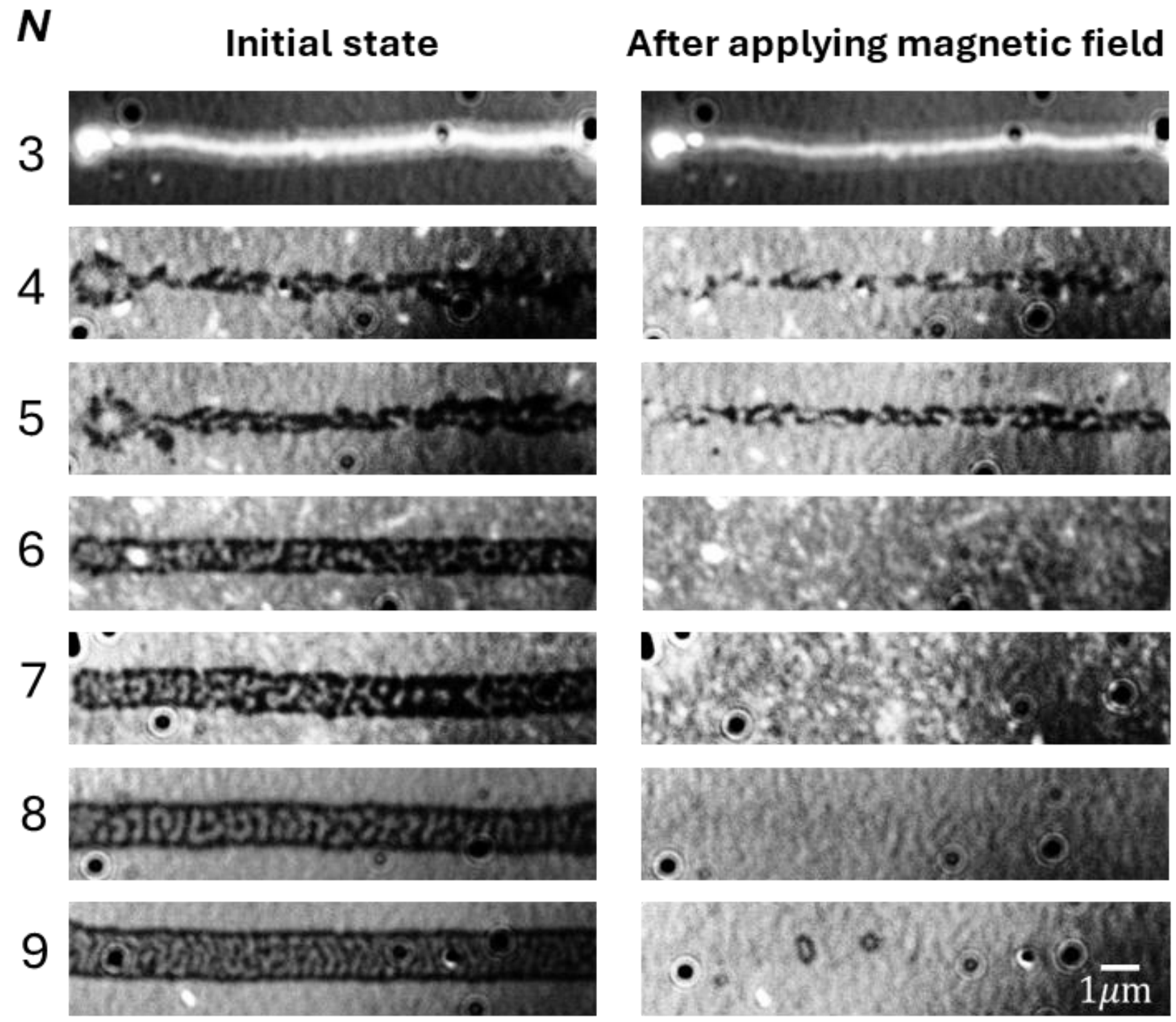

Fig. S14 Conventional circularly polarized Gaussian-beam exposure for an initial magnetization state of M^+ and incident RCP polarization. For N = 3, 4 and 5, the exposure caused permanent sample damage. For N = 6, 7, 8 and 9, laser pulses produced non-deterministic magnetic domain formation, with multiple randomly nucleated domains instead of a well-defined switched region. The first column shows the magnetic state after exposure to femtosecond laser pulses, while the second column shows the same regions after applying an external magnetic field strong enough to saturate the thin film back to its pre-switched magnetization direction.

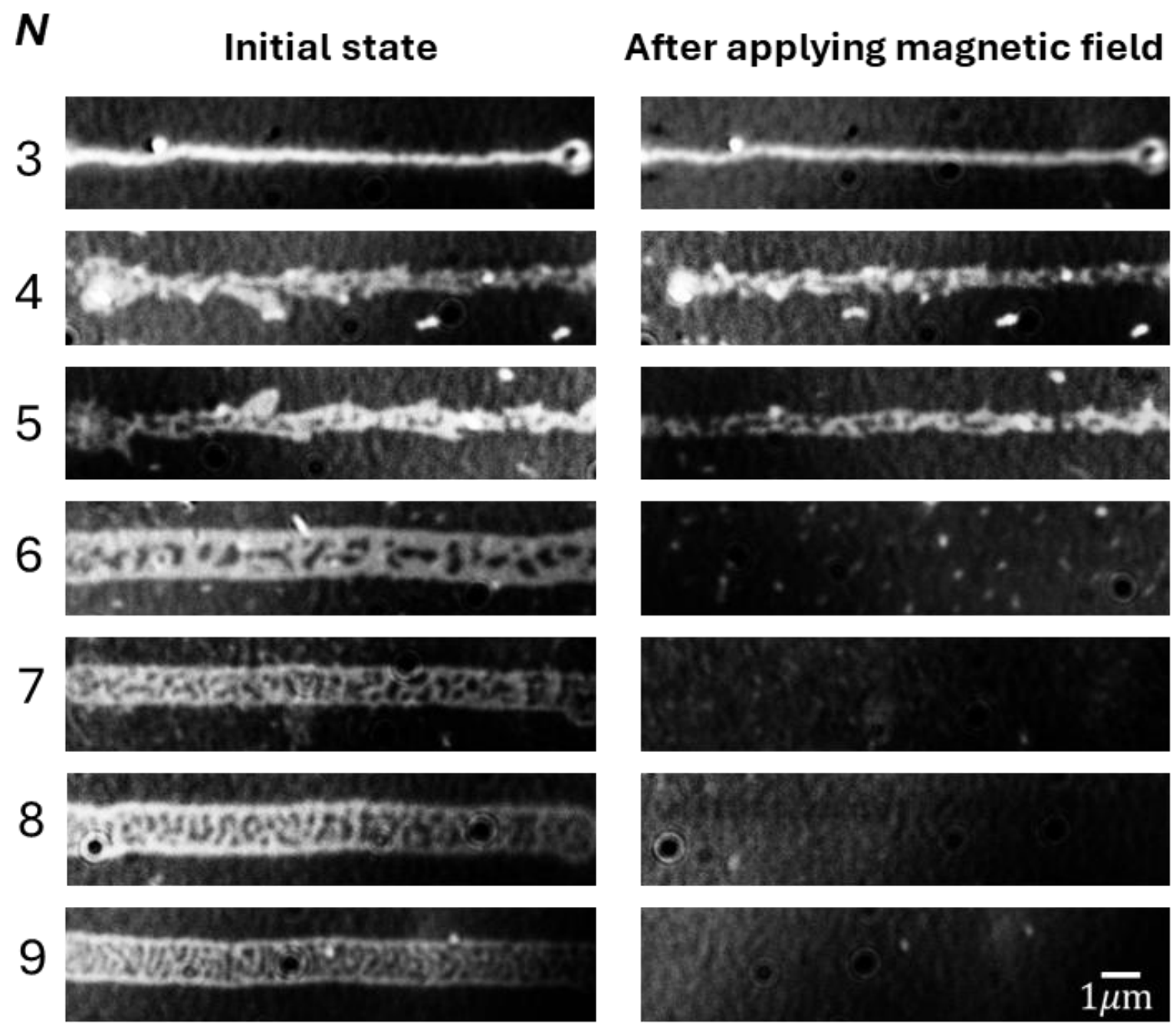

Fig. S15 Conventional circularly polarized Gaussian-beam exposure for an initial magnetization state of M^- and incident RCP polarization. For N=3, 4 and 5, the exposure caused permanent sample damage. For N = 6, 7, 8 and 9, it produced non-deterministic magnetic domain formation, with multiple randomly nucleated domains instead of a well-defined switched region. The first column shows the magnetic state after exposure to femtosecond laser pulses, while the second column shows the same regions after applying an external magnetic field strong enough to saturate the thin film back to its pre-switched magnetization direction

These additional experimental results directly support the proposed mechanism: wavelength-scale confinement of the vector beam enables deterministic and spatially uniform switching, which is in contrast to the Gaussian beam. As a result, for the same conditions the Gaussian beam fails to switch. This behavior reflects a fundamental limitation identified in Fig. 1 of the main text and Supplementary Figs. S2–S4 of the referenced work [1], a limitation that subsequent studies also continued to encounter. In the present work, we overcome this constraint through optical beam engineering, enabling spatially confined and deterministic optical switching that is not achievable under conventional Gaussian-beam excitation. Importantly, we also observe this transition clearly for N = 3, where only the tightly confined vector beam produces helicity-independent switching, indicating that local optical confinement together with the surrounding dipolar-field landscape is essential for deterministic switching.

Overall, these measurements directly address the reviewer's proposed comparison and clarify the role of vectorial fields in the observed switching. Under the experimental conditions studied here, the conventional circularly polarized Gaussian beam produced qualitatively different

outcomes, including irreversible modification or non-deterministic domain formation depending on the multilayer thickness, rather than the deterministic switching obtained with the vector beam. The present control experiment supports our conclusion that the vector-beam configuration is essential for achieving the deterministic switching response reported in this work.

Reference

1. Lambert, Charles-Henri, Stéphane Mangin, BSD Ch S. Varaprasad, Y. K. Takahashi, Michel Hehn, M. Cinchetti, Grégory Malinowski et al. "All-optical control of ferromagnetic thin films and nanostructures." *Science* 345, no. 6202 (2014): 1337-1340.

Response to Reviewer #3

Comment **Response**

Comment text …………… Reviewer **Response text …………… Authors**

Reviewer #3 (Remarks to the Authors):

I am satisfied with the author's response and the revised manuscript. I recommend the publication of the modified version in Nature Communications.

Response of authors

We thank the reviewer for her/his positive assessment of our revised manuscript and for recommending publication of the modified version in *Nature Communications*. We sincerely appreciate the reviewer's careful reading of the manuscript and her/his constructive comments during the review process. The feedback helped us clarify the central claims of the work, strengthen the presentation of the experimental results, and improve the overall readability of the manuscript. We are pleased that the revisions have addressed the reviewer's concerns, and we are grateful for the support of the manuscript's publication.
